# Supplementary material for: Phosphorylation of DGCR8 Increases Its Intracellular Stability and Induces a Progrowth miRNA Profile
Source: Cell Rep. Author manuscript; Available in PMC 2014 Nov 27. (PMC3892995; doi:10.1016/j.celrep.2013.10.017)
Supplement: 1 [file NIHMS542053-supplement-1.pdf]

Phosphorylation of DGCR8 Increases Its Intracellular Stability and Induces a Pro-growth miRNA Profile

Kristina M. Herbert,[1] Genaro Pimienta,[1] Suzanne J. DeGregorio,[1] Andrei Alexandrov,[1] and Joan A. Steitz[1,]*

[1] Department of Molecular Biophysics and Biochemistry, Howard Hughes Medical Institute, Yale University School of Medicine, New Haven, CT 06536, USA

*Correspondence: joan.steitz@yale.edu

SUPPLEMENTAL INFORMATION:

Figures S1- S5

Tables S1, S2, and S3

Captions for Tables S1-S6 and Data S1

Supplemental Experimental Procedures

Supplemental References

INVENTORY OF SUPPLEMENTAL INFORMATION

Figures:

Figure S1. Sequence Coverage; Related to Figure 1C. This figure displays the DGCR8 amino acid sequences that were covered in our mass spectrometry data.

Figure S2. DGCR8 Is Targeted by MAPKs; Related to Figure 2. This figure shows the identified MAPK docking motifs in DGCR8 (A), in vitro kinase assays for JNK /MAPK (B), confirmation that we were able to isolate activated ERK (C), and p-JNK immunoblot showing that JNK was not activated upon serum addition to HeLa cells.

Figure S3. DGCR8 Decay Is Responsible for Differences in DGCR8 Levels; Related to Figure 3. This figure shows decay data for DGCR8 mutants in HeLa strain 1.

Figure S4. Microprocessor Complexes Containing Phosphomimetic DGCR8 Do Not Exhibit Altered Specific Pri-miRNA Processing Activity *In Vitro*; Related to Figure 5. This figure shows in vitro processing assays for MC isolated from HEK 293T cells for several pri-miRNAs.

Figure S5. MiRNA Expression Profiles; Related to Figure 5. This figure presents controls for the RNA-seq data presented in Figure 5.

Tables:

Table S1. Phosphosites Mapped with Poor Statistics in DGCR8 Expressed in HEK293 Cells; Related to Table 1. This table presents data on additional phosphosites that were mapped but were not deemed statistically significant.

Table S2. Mutant and Mimetic DGCR8 Constructs; Related to Figure 1F. This table presents the sequences of all of the mutant DGCR8 constructs created for this manuscript.

Table S3. Potential Kinases; Related to Figure 2. This table presents kinases predicted to phosphorylate each of the identified phosphosites.

Table S4. 75 MiRNAs that Show a Greater than 2-fold Up-Regulation in the Mim23-Expressing Cells Relative to Both WT- and Mut23-Expressing Cells; Related to Figure 5.

Table S5. 7 MiRNAs that Show a Greater than 2-fold Up-Regulation in the Mim23-Expressing Cells Relative to Both WT- and Mut23-Expressing Cells; Related to Figure 5.

Table S6. 534 MiRNAs that Show Less than a 2-fold Change in the Mim23-Expressing Cells Relative to Either WT- and/or Mut23-Expressing Cells; Related to Figure 5.

Data Files:

Data S1. Spectral Evidence for All DGCR8 Phosphosites Identified; Related to Figure 1D.

Additional Information:

Supplemental Experimental Procedures

Supplemental References

SUPPLEMENTAL INFORMATION

Figure S1. Sequence Coverage; Related to Figure 1C. The DGCR8 amino acid sequence is shown in gray with the peptide sequences that were identified in each mass-spectrometry experiment shown in bold. Identified phosphosites from each experiment are highlighted in green.

Insect Cell Samples

TiO_2 Elute Sequence coverage = 49.2 %

```
        10         20         30         40         50         60         70         80         90        100
METDESPSPL PCGPAGEAVM ESRARPFQAL PREQSPPPPL QTSSGAEVMD VGSGGDGQSE LPAEDPFNFY GASLLSKGSF SKGRLLIDPN CSGHSPRTAR
HAPAVRKFSP DLKLLKDVKI SVSFTESCRS KDRKVLYTGA ERDVRAECGL LLSPVSGDVH ACPFGGSVGD GVGIGGESAD KKDEENELDQ EKRVEYAVLD
ELEDFTDNLE LDEEGAGGFT AKAIVQRDRV DEEALNFPYE DDFDNDVDAL LEEGLCAPKK RRTEEKYGGD SDHPSDGETS VQPMMTKIKT VLKSRGRPPT
EPLPDGWIMT FHNSGVPVYL HRESRVVTWS RPYFLGTGSI RKHDPPLSSI PCLHYKKMKD NEEREQSSDL TPSGDVSPVK PLSRSAELEF PLDEPDSMGA
DPGPPDEKDP LGAEAAPGAL GQVKAKVEVC KDESVDLEEF RSYLEKRFDF EQVTVKKFRT WAERRQFNRE MKRKQAESER PILPANQKLI TLSVQDAPTK
KEFVINPNGK SEVCILHEYM QRVLKVRPVY NFFECENPSE PFGASVTIDG VTYGSGTASS KKLAKNKAAR ATLEILIPDF VKQTSEEKPK DSEELEYFNH
ISIEDSRVYE LTSKAGLLSP YQILHECLKR NHGMGDTSIK FEVVPGKNQK SEYVMACGKH TVRGWCKNKR VGKQLASQKI LQLLHPHVKN WGSLLRMYGR
ESSKMVKQET SDKSVIELQQ YAKKNKPNLH ILSKLQEEMK RLAEEREETR KKPKMSIVAS AQPGGEPLCT VDV
```

IMAC Elute Sequence coverage = 18.1 %

```
        10         20         30         40         50         60         70         80         90        100
METDESPSPL PCGPAGEAVM ESRARPFQAL PREQSPPPPL QTSSGAEVMD VGSGGDGQSE LPAEDPFNFY GASLLSKGSF SKGRLLIDPN CSGHSPRTAR
HAPAVRKFSP DLKLLKDVKI SVSFTESCRS KDRKVLYTGA ERDVRAECGL LLSPVSGDVH ACPFGGSVGD GVGIGGESAD KKDEENELDQ EKRVEYAVLD
ELEDFTDNLE LDEEGAGGFT AKAIVQRDRV DEEALNFPYE DDFDNDVDAL LEEGLCAPKK RRTEEKYGGD SDHPSDGETS VQPMMTKIKT VLKSRGRPPT
EPLPDGWIMT FHNSGVPVYL HRESRVVTWS RPYFLGTGSI RKHDPPLSSI PCLHYKKMKD NEEREQSSDL TPSGDVSPVK PLSRSAELEF PLDEPDSMGA
DPGPPDEKDP LGAEAAPGAL GQVKAKVEVC KDESVDLEEF RSYLEKRFDF EQVTVKKFRT WAERRQFNRE MKRKQAESER PILPANQKLI TLSVQDAPTK
KEFVINPNGK SEVCILHEYM QRVLKVRPVY NFFECENPSE PFGASVTIDG VTYGSGTASS KKLAKNKAAR ATLEILIPDF VKQTSEEKPK DSEELEYFNH
ISIEDSRVYE LTSKAGLLSP YQILHECLKR NHGMGDTSIK FEVVPGKNQK SEYVMACGKH TVRGWCKNKR VGKQLASQKI LQLLHPHVKN WGSLLRMYGR
ESSKMVKQET SDKSVIELQQ YAKKNKPNLH ILSKLQEEMK RLAEEREETR KKPKMSIVAS AQPGGEPLCT VDV
```

IMAC FT Sequence coverage = 67.0 %

```
        10         20         30         40         50         60         70         80         90        100
METDESPSPL PCGPAGEAVM ESRARPFQAL PREQSPPPPL QTSSGAEVMD VGSGGDGQSE LPAEDPFNFY GASLLSKGSF SKGRLLIDPN CSGHSPRTAR
HAPAVRKFSP DLKLLKDVKI SVSFTESCRS KDRKVLYTGA ERDVRAECGL LLSPVSGDVH ACPFGGSVGD GVGIGGESAD KKDEENELDQ EKRVEYAVLD
ELEDFTDNLE LDEEGAGGFT AKAIVQRDRV DEEALNFPYE DDFDNDVDAL LEEGLCAPKK RRTEEKYGGD SDHPSDGETS VQPMMTKIKT VLKSRGRPPT
EPLPDGWIMT FHNSGVPVYL HRESRVVTWS RPYFLGTGSI RKHDPPLSSI PCLHYKKMKD NEEREQSSDL TPSGDVSPVK PLSRSAELEF PLDEPDSMGA
DPGPPDEKDP LGAEAAPGAL GQVKAKVEVC KDESVDLEEF RSYLEKRFDF EQVTVKKFRT WAERRQFNRE MKRKQAESER PILPANQKLI TLSVQDAPTK
KEFVINPNGK SEVCILHEYM QRVLKVRPVY NFFECENPSE PFGASVTIDG VTYGSGTASS KKLAKNKAAR ATLEILIPDF VKQTSEEKPK DSEELEYFNH
ISIEDSRVYE LTSKAGLLSP YQILHECLKR NHGMGDTSIK FEVVPGKNQK SEYVMACGKH TVRGWCKNKR VGKQLASQKI LQLLHPHVKN WGSLLRMYGR
ESSKMVKQET SDKSVIELQQ YAKKNKPNLH ILSKLQEEMK RLAEEREETR KKPKMSIVAS AQPGGEPLCT VDV
```

Total Sequence coverage = 73.5 %

```
        10         20         30         40         50         60         70         80         90        100
METDESPSPL PCGPAGEAVM ESRARPFQAL PREQSPPPPL QTSSGAEVMD VGSGGDGQSE LPAEDPFNFY GASLLSKGSF SKGRLLIDPN CSGHSPRTAR
HAPAVRKFSP DLKLLKDVKI SVSFTESCRS KDRKVLYTGA ERDVRAECGL LLSPVSGDVH ACPFGGSVGD GVGIGGESAD KKDEENELDQ EKRVEYAVLD
ELEDFTDNLE LDEEGAGGFT AKAIVQRDRV DEEALNFPYE DDFDNDVDAL LEEGLCAPKK RRTEEKYGGD SDHPSDGETS VQPMMTKIKT VLKSRGRPPT
EPLPDGWIMT FHNSGVPVYL HRESRVVTWS RPYFLGTGSI RKHDPPLSSI PCLHYKKMKD NEEREQSSDL TPSGDVSPVK PLSRSAELEF PLDEPDSMGA
DPGPPDEKDP LGAEAAPGAL GQVKAKVEVC KDESVDLEEF RSYLEKRFDF EQVTVKKFRT WAERRQFNRE MKRKQAESER PILPANQKLI TLSVQDAPTK
KEFVINPNGK SEVCILHEYM QRVLKVRPVY NFFECENPSE PFGASVTIDG VTYGSGTASS KKLAKNKAAR ATLEILIPDF VKQTSEEKPK DSEELEYFNH
ISIEDSRVYE LTSKAGLLSP YQILHECLKR NHGMGDTSIK FEVVPGKNQK SEYVMACGKH TVRGWCKNKR VGKQLASQKI LQLLHPHVKN WGSLLRMYGR
ESSKMVKQET SDKSVIELQQ YAKKNKPNLH ILSKLQEEMK RLAEEREETR KKPKMSIVAS AQPGGEPLCT VDV
```

Mammalian Cell Samples

Experiment 1 Sequence coverage = 52.9 %

```
        10         20         30         40         50         60         70         80         90        100
METDESPSPL PCGPAGEAVM ESRARPFQAL PREQSPPPPL QTSSGAEVMD VGSGGDGQSE LPAEDPFNFY GASLLSKGSF SKGRLLIDPN CSGHSPRTAR
HAPAVRKFSP DLKLLKDVKI SVSFTESCRS KDRKVLYTGA ERDVRAECGL LLSPVSGDVH ACPFGGSVGD GVGIGGESAD KKDEENELDQ EKRVEYAVLD
ELEDFTDNLE LDEEGAGGFT AKAIVQRDRV DEEALNFPYE DDFDNDVDAL LEEGLCAPKK RRTEEKYGGD SDHPSDGETS VQPMMTKIKT VLKSRGRPPT
EPLPDGWIMT FHNSGVPVYL HRESRVVTWS RPYFLGTGSI RKHDPPLSSI PCLHYKKMKD NEEREQSSDL TPSGDVSPVK PLSRSAELEF PLDEPDSMGA
DPGPPDEKDP LGAEAAPGAL GQVKAKVEVC KDESVDLEEF RSYLEKRFDF EQVTVKKFRT WAERRQFNRE MKRKQAESER PILPANQKLI TLSVQDAPTK
KEFVINPNGK SEVCILHEYM QRVLKVRPVY NFFECENPSE PFGASVTIDG VTYGSGTASS KKLAKNKAAR ATLEILIPDF VKQTSEEKPK DSEELEYFNH
ISIEDSRVYE LTSKAGLLSP YQILHECLKR NHGMGDTSIK FEVVPGKNQK SEYVMACGKH TVRGWCKNKR VGKQLASQKI LQLLHPHVKN WGSLLRMYGR
ESSKMVKQET SDKSVIELQQ YAKKNKPNLH ILSKLQEEMK RLAEEREETR KKPKMSIVAS AQPGGEPLCT VDV
```

Experiment 2 Sequence coverage = 60.0 %

```
        10         20         30         40         50         60         70         80         90        100
METDESPSPL PCGPAGEAVM ESRARPFQAL PREQSPPPPL QTSSGAEVMD VGSGGDGQSE LPAEDPFNFY GASLLSKGSF SKGRLLIDPN CSGHSPRTAR
HAPAVRKFSP DLKLLKDVKI SVSFTESCRS KDRKVLYTGA ERDVRAECGL LLSPVSGDVH ACPFGGSVGD GVGIGGESAD KKDEENELDQ EKRVEYAVLD
ELEDFTDNLE LDEEGAGGFT AKAIVQRDRV DEEALNFPYE DDFDNDVDAL LEEGLCAPKK RRTEEKYGGD SDHPSDGETS VQPMMTKIKT VLKSRGRPPT
EPLPDGWIMT FHNSGVPVYL HRESRVVTWS RPYFLGTGSI RKHDPPLSSI PCLHYKKMKD NEEREQSSDL TPSGDVSPVK PLSRSAELEF PLDEPDSMGA
DPGPPDEKDP LGAEAAPGAL GQVKAKVEVC KDESVDLEEF RSYLEKRFDF EQVTVKKFRT WAERRQFNRE MKRKQAESER PILPANQKLI TLSVQDAPTK
KEFVINPNGK SEVCILHEYM QRVLKVRPVY NFFECENPSE PFGASVTIDG VTYGSGTASS KKLAKNKAAR ATLEILIPDF VKQTSEEKPK DSEELEYFNH
ISIEDSRVYE LTSKAGLLSP YQILHECLKR NHGMGDTSIK FEVVPGKNQK SEYVMACGKH TVRGWCKNKR VGKQLASQKI LQLLHPHVKN WGSLLRMYGR
ESSKMVKQET SDKSVIELQQ YAKKNKPNLH ILSKLQEEMK RLAEEREETR KKPKMSIVAS AQPGGEPLCT VDV
```

Table S1. Phosphosites Mapped with Poor Statistics in DGCR8 Expressed in HEK293 Cells; Related to Table 1. The amino acid number and residue of each mapped phosphosite is given. The posterior error probability (PEP), MaxQuant score, as well as the number of phosphopeptides identified in each experimental replicate (Experiment 1 or 2) or condition (with or without calyculin A treatment) are shown.

	**HEK293 cells Experiment 1**					**HEK293 cells Experiment 2**				
	Coverage 52.9%		Number of peptides phosphorylated			Coverage 60.0%		Number of peptides phosphorylated		
aa sites	PEP	score	total	no treat-ment	calyculin	PEP	score	total	no treat-ment	calyculin
S127	NA	NA	0/0	0	0	6.38E-02	39.39	1/37	0	1
T138	1.47E+02	28.869	1/44	0	1	NA	NA	0/28	0	0
T454	5.16E-01	9.1985	1/54	0	1	NA	NA	0/74	0	0
S478	3.55E-01	25.94	1/39	0	1	NA	NA	0/33	0	0
T710	NA	NA	0/15	0	0	2.62E-01	22.777	1/2	0	1
S714	9.56E-02	58.918	3/59	0	3	NA	NA	0/19	0	0

Data S1. Spectral Evidence for All DGCR8 Phosphosites Identified; Related to Figure 1D. See supplemental pdf file.

Shown are representative fragmentation spectra of phosphopeptides for each of the 23 phosphosites investigated in this work (Figure 1E), as well as those for which the statistical significance was not as great (Supplementary Table 1). Labels indicate the phosphosite identified in each fragmentation pattern. Spectra are shown as displayed by the MaxQuant-Andromeda output. The header in each spectrum includes the different ProteinKnowledgeBase (UniProt-KB) entries of the protein identified (Protein), which in this case is always DGCR8. Next, the identification score is shown (Score), followed by the raw data file from which the shown spectrum was retrieved (Source), Scan number (Scannumber), and the instrument setting (Method). In our experiments, the Method corresponds to ion trap (IT) mass spectrometry (MS) followed by collision-induced dissociation (CID) tandem mass spectrometry (MS2; or 2). The “b” and “y” fragmentation ions are indicated in blue and red, respectively, along the peptide sequence assigned to the spectrum and in the fragmentation spectrum itself. Phosphorylated peptides have a superscript “ph” tag. The other modification shown is “ox”, referring to methionine oxidation. The meaning of superscript labels in the spectra is as follows: -H20 (water loss); -NH3 (ammonium loss); * (oxidation); and 2+ or 3+ (charge). A large fraction of the phosphopeptides identified has more than 1 phosphorylated residue and/or are longer than 20 amino acids. MaxQuant-Andromeda, the search engine used in our studies, was developed to identify peptides with such characteristics (Cox et al., 2011).

Table S2. Mutant and Mimetic DGCR8 Constructs; Related to Figure 1F. DNA sequences and amino acids (aa) of the WT, Mutant (Mut23 and Mut14), and Mimetic (Mim23 and Mim11) DGCR8 constructs are provided for each identified phosphosite. • indicates that a specific aa residue is mutated to either prevent or mimic phosphorylation, while an empty cell/box indicates that the sequence is WT. Mutation sites for Mim11 and Mut14 were selected to be scattered uniformly along the protein primary sequence.

	WT		Mutant				Mimetic			
aa sites	DNA seq	aa	DNA seq	aa	Mut23	Mut14	DNA seq	aa	Mim23	Mim11
35	TCT	Ser	GTT	Val	•	•	GAT	Asp	•	•
42	ACG	Thr	GCG	Ala	•	•	GAC	Asp	•	
59	TCC	Ser	GTC	Val	•		GAC	Asp	•	
92	AGT	Ser	GAT	Val	•	•	GTT	Asp	•	
95	AGC	Ser	GTC	Val	•	•	GAC	Asp	•	•
109	TCC	Ser	GTC	Val	•	•	GAC	Asp	•	•
123	AGC	Ser	GTC	Val	•		GAC	Asp	•	
125	TGG	Thr	GCC	Ala	•		GAC	Asp	•	
153	AGC	Ser	GTC	Val	•	•	GAC	Asp	•	•
156	AGT	Ser	GAT	Val	•	•	GTT	Asp	•	•
267	TAT	Tyr	TTT	Phe	•		GAG	Glu	•	
271	AGC	Ser	GTC	Val	•		GAC	Asp	•	•
275	TCC	Ser	GTC	Val	•	•	GAC	Asp	•	•
279	ACA	Thr	GCA	Ala	•		GAC	Asp	•	•
280	TCA	Ser	GTT	Val	•		GAT	Asp	•	
371	ACC	Thr	GCC	Ala	•	•	GAC	Asp	•	
373	AGT	Ser	GAT	Val	•	•	GTT	Asp	•	•
377	TCC	Ser	GTC	Val	•	•	GAC	Asp	•	
383	AGC	Ser	GTC	Val	•	•	GAC	Asp	•	•
385	TCT	Ser	GTT	Val	•		GAT	Asp	•	•
397	TCT	Ser	GTT	Val	•	•	GAT	Asp	•	
434	TCC	Ser	GTC	Val	•	•	GAC	Asp	•	
493	TCA	Ser	GTA	Val	•		GAC	Asp	•	

Table S3. Potential Kinases; Related to Figure 2. Two programs that match input protein sequences with post-translational modification motifs were used to predict potential kinases for each identified phosphosite. PHOSIDA motif matcher (Gnad et al., 2011; Gnad et al., 2007) identifies a class of candidate kinases, while NetworKin beta (Linding et al., 2007) reveals the gene name of a putative kinase.

aa sites	PHOSIDA	NetworKin beta
35	CAMK2, PKD, CHK1/2, CHK1	CDK2, CDK3
42	no prediction	STK11, NEK2
59	no prediction	CSNK2A2,CK2A1
92	no prediction	PRKAA1, PRKAA2 NEK2,
95	CK1, CDK1	CDK2, CDK3
109	PKA, CAMK2	MAPK14, MAPK11, MAPK13, MAPK12, CAMK2G, CDK2, CDK3
123	CK2, GSK3	CSNK2A2,CK2A1
125	no prediction	TGFBR2, ACVR2B, PRKAA1, PRKAA2, ADRBK1, GRK1, GRK5, ADRBK2
153	NEK6	MAPK14, MAPK11, MAPK13, MAPK12, MAPK9, MAPK10, MAPK8, DMK, CDC42BPA
156	CK1	PRKAA1, PRKAA2, MOK, ICK, CSNK2A2,CK2A1
267	SRC	IGF1R, INSR, LYN, SRC, LCK, FYN, FGR, HCK, BLK, PTK6, YES1, MAP2K6, MAP2K4, MAP2K3
271	GSK3	CSNK2A2,CK2A1
275	CK1, CK2	CSNK2A2,CK2A1
279	no prediction	TGFBR2, ACVR2B, TLK1, PRKDC,
280	PLK1	TLK1
371	CK1	CDK2, CDK3
373	GSK3, NEK6	GSK3B, GSK3A
377	CK1, CDK2, CDK1	CDK2, CDK3, MAPK14, MAPK11, MAPK13, MAPK12, MAPK9, MAPK10, MAPK8,
383	no prediction	MAPKAPK5, MAPKAPK2, MAPKAPK3, MAP2K6, MAP2K4, MAP2K3, DMK, CDC42BPA, MOK, ICK, PAK2, PAK3, CAMK2G, CLK1, CLK2
385	NEK6	DMK, CDC42BPA
397	no prediction	CSNK2A2,CK2A1
434	PKD, PLK1, CHK1	CSNK2A2,CK2A1
493	no prediction	DMK, CDC42BPA

Figure S2. DGCR8 Is Targeted by MAPKs; Related to Figure 2. (A) MAPKs bind characteristic primary amino acid sequences within target proteins, known as D-sites (Garai et al., 2012). Shown are sequences within DGCR8 that match this D-site motif, where X and ϕ correspond to any amino acid and a hydrophobic amino acid, respectively. (B) FLAG-JNK1a1 was immunoprecipitated from HEK293T cells that had been transfected with WT or the APF mutant FLAG-JNK1a1, or FLAG-MKK7B2 fusions of WT or the APF mutant JNK1a1. Immunoblots of these immunoprecipitates were probed with anti-FLAG to confirm protein presence and anti-pJNK to confirm activation of the constitutively activated construct. (C) HA-ERK was immunoprecipitated from HEK293T cells that had been transfected with either GFP alone, as a negative control (-), or HA-ERK together with MAPKK1-K97M, MAPKK1-R4F, or GFP. Immunoblots of these immunoprecipitates were probed with anti-HA to confirm protein presence and anti-pERK to confirm activation of ERK in the presence of its constitutively activated upstream kinase. (D) Strain 1 HeLa Flp-In cells stably expressing WT-F-DGCR8 or an empty vector were serum starved overnight, then treated for 2 hr with either DMSO control, U0126 (MEK1/2 inhibitor), or SP600125 (JNK inhibitor). Cells were then metabolically labeled with $^{32}PO_4$ upon serum addition for 4 hr. Immunoblots of total cell lysates (input) were probed for p-JNK activity.

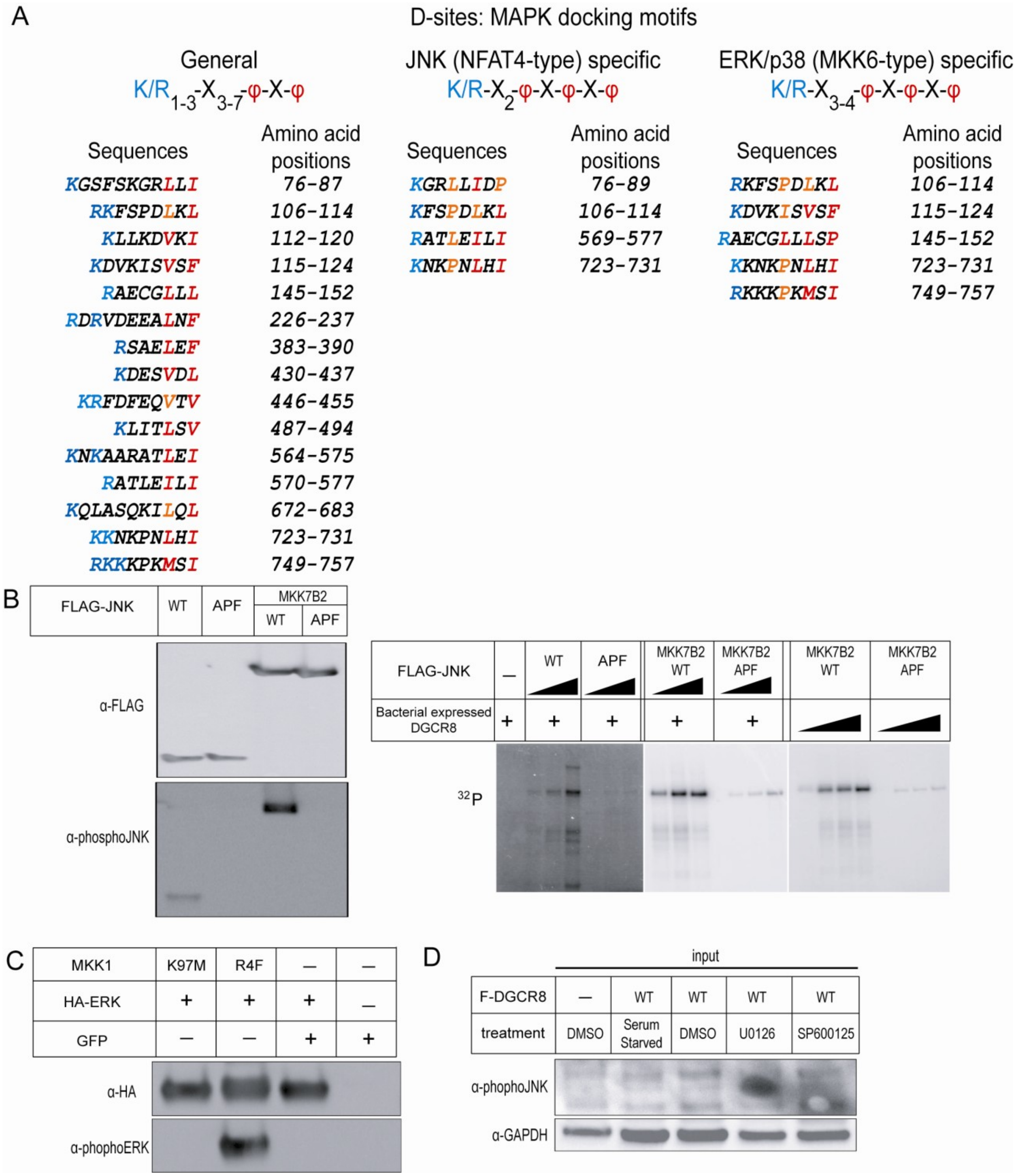
A
D-sites: MAPK docking motifs
General
K/R1-3-X3-7-φ-X-φ
Sequences
Amino acid positions
KGSFSKGRLLI 76-87
RKFSPDLKL 106-114
KLLKDVKI 112-120
KDVKISVSF 115-124
RAECGLLL 145-152
RDRVDEEALNF 226-237
RSAELEF 383-390
KDESVDL 430-437
KRFDFEQVTV 446-455
KLITLSV 487-494
KNKAARATLEI 564-575
RATLEILI 570-577
KQLASQKILQL 672-683
KKNKPNLHI 723-731
RKKKPKMSI 749-757
JNK (NFAT4-type) specific
K/R-X2-φ-X-φ-X-φ
KGRLLIDP 76-89
KFSPDLKL 106-114
RATLEILI 569-577
KNKPNLHI 723-731
ERK/p38 (MKK6-type) specific
K/R-X3-4-φ-X-φ-X-φ
RKFSPDLKL 106-114
KDVKISVSF 115-124
RAECGLLLSP 145-152
KKNKPNLHI 723-731
RKKKPKMSI 749-757
B
FLAG-JNK
WT
APF
MKK7B2
WT
APF
α-FLAG
α-phosphoJNK
FLAG-JNK
—
WT
APF
MKK7B2 WT
MKK7B2 APF
Bacterial expressed DGCR8
+
32P
C
MKK1
K97M
R4F
HA-ERK
GFP
α-HA
α-phophoERK
D
input
F-DGCR8
treatment
DMSO
Serum Starved
U0126
SP600125
α-phophoJNK
α-GAPDH

Figure S3. DGCR8 Decay Is Responsible for Differences in DGCR8 Levels; Related to Figure 3. Strain 1 isogenic HeLa Flp-In cells stably expressing WT-, Mut23-, or Mim23-F-DGCR8 were treated with 100 μg/mL cycloheximide. Cells were harvested at 0, 4, and 16 hr. Immunoblots were performed on total cell lysates to monitor DGCR8 decay.

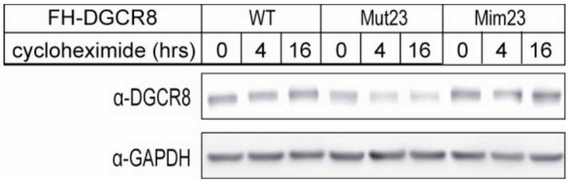

Figure S4. Microprocessor Complexes Containing Phosphomimetic DGCR8 Do Not Exhibit Altered Specific Pri-miRNA Processing Activity *In Vitro*; Related to Figure 5. (A) *In vitro* pri-miRNA-processing assays were performed by incubating various body-labeled pri-miRNAs and a short (35 nt) stable RNA, which functioned as a loading control (LC), with immunoprecipitated MCs from HEK293T cells that had been transiently transfected with GFP, Myc-Drosha, and either an empty vector or a vector expressing WT-, Mut23-, or Mim23-FH-DGCR8. The input RNAs are shown in the lanes labeled RNA. * mark the pre-miR species. Contrast has been adjusted separately on the ladder lanes. (B) Immunoblots of MCs isolated via anti-FLAG immunoprecipitation of lysates of HEK293T cells transiently transfected with vectors expressing GFP, Myc-Drosha, and either an empty vector, or a vector expressing WT-, Mut23-, or Mim23-FH-DGCR8. These MC immunoprecipitates were used for the processing assays shown in the top panels of A. (C) HEK293T cells were transiently transfected with vectors expressing GFP, Myc-Drosha, and either an empty vector, or a vector expressing WT-, Mut23-, Mim23-FH-DGCR8, or WT-SNAP-DGCR8. Immunoblots of anti-FLAG immunoprecipitated MCs were probed for p68 and p72 helicases as well as for GAPDH.

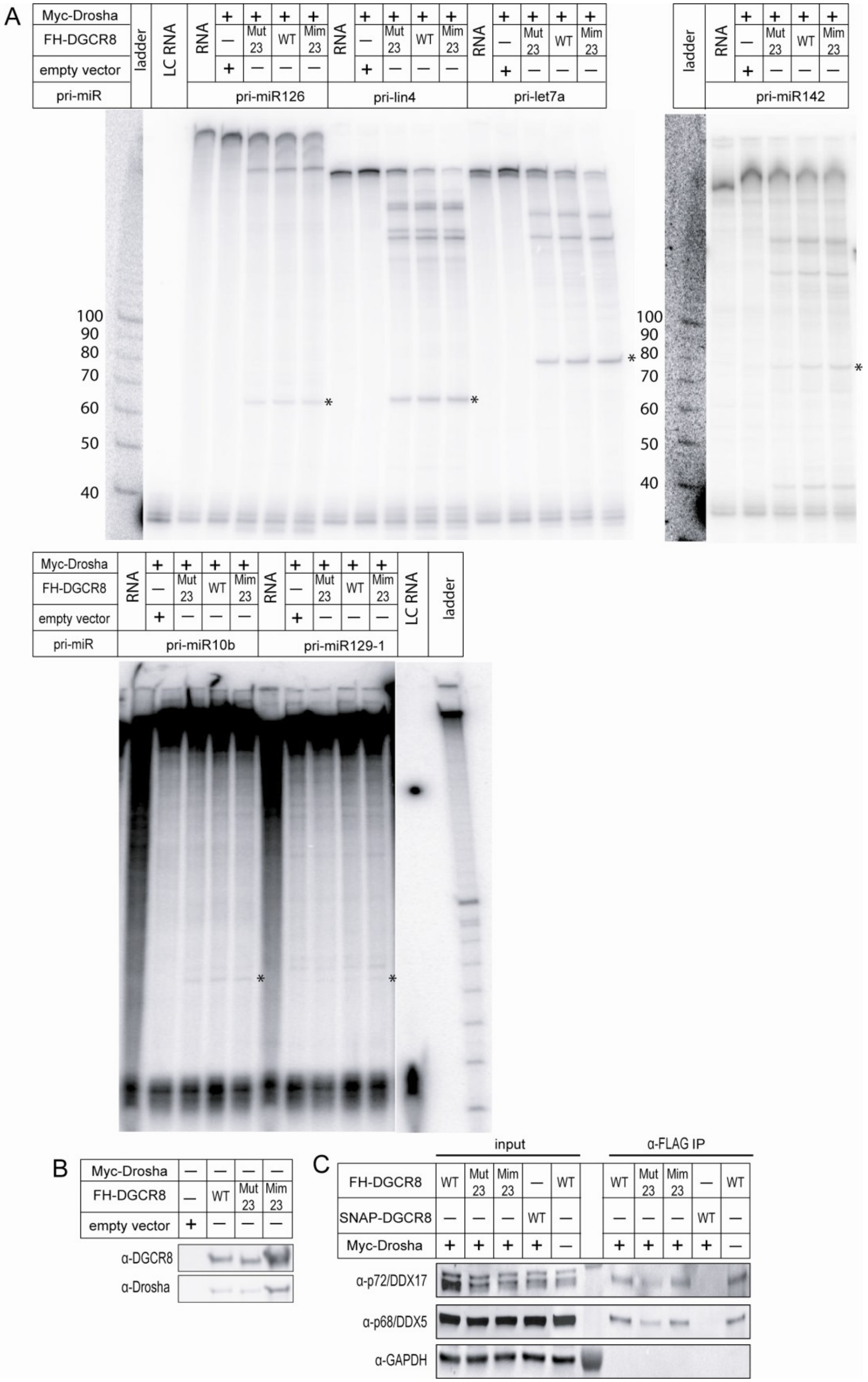
A
Myc-Drosha
FH-DGCR8
empty vector
pri-miR
ladder
LC RNA
RNA
Mut 23
WT
Mim 23
pri-miR126
pri-lin4
pri-let7a
pri-miR142
100
90
80
70
60
50
40
*
pri-miR10b
pri-miR129-1
B
α-DGCR8
α-Drosha
C
input
α-FLAG IP
SNAP-DGCR8
α-p72/DDX17
α-p68/DDX5
α-GAPDH

Figure S5. MiRNA Expression Profiles; Related to Figure 5. (A) Next-generation sequencing was used to profile levels of small RNAs from strain 2 isogenic HeLa Flp-In cells stably expressing Mim23-, WT-, or Mut23-F-DGCR8. Each dot represents, for an individual mature miRNA, the $\log_2$ relative expression in Mim23- over Mut23-F-DGCR8 cells versus the $\log_2$ relative expression in Mim23- over WT-F-DGCR8 cells. Dotted lines are shown at 1 and -1, corresponding to a 2-fold change up or down, respectively; thus, a miRNA with a greater than 2-fold up or down change in the Mim23 sample relative to both the Mut23 and WT sample will be in the upper right or lower left quadrant. Red, blue, and green dots represent separate biological replicates, while black shows the average value for each miRNA from all three replicates. Error bars are omitted for simplicity but are given in Tables S4-6. (B) Plots showing positive correlations between biological replicates of the next-generation sequencing $\log_2$ relative expression data for each mature miRNA. (C) TaqMan miRNA quantitative PCR was performed to assess expression (mean ± STD) of miR-181a (n = 3), and miR-93 (n = 2), miR-129 (n=3), miR-10b (n=4) relative to U6 in strain 2 HeLa cells stably expressing Mim23-, Mut23-, or WT-F-DGCR8.

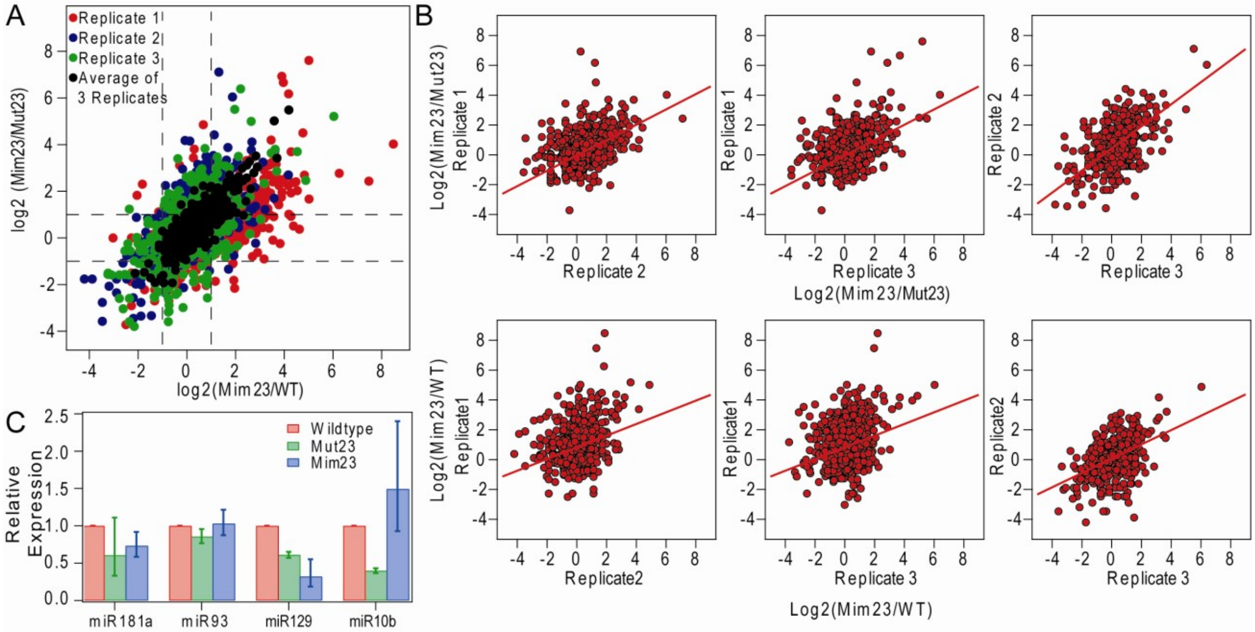

Table S4. 75 MiRNAs that Show a Greater than 2-fold Up-Regulation in the Mim23-Expressing Cells Relative to Both WT- and Mut23-Expressing Cells; Related to Figure 5. The table shows the miRNA id, the sum of reads from all samples analyzed that mapped to this miRNA, the precursor id corresponding to the mature miRNA, the average and standard error (n=3) of the $\log_2$ relative expression in Mim23- compared to Mut23-F-DGCR8-expressing cells, the average and standard error (n=3) of the $\log_2$ relative expression in Mim23- compared to WT-F-DGCR8-expressing cells.

Table S5. 7 MiRNAs that Show a Greater than 2-fold Up-Regulation in the Mim23-Expressing Cells Relative to Both WT- and Mut23-Expressing Cells; Related to Figure 5. The table shows the miRNA id, the sum of reads from all samples analyzed that mapped to this miRNA, the precursor id corresponding to the mature miRNA, the average and standard error (n=3) of the $\log_2$ relative expression in Mim23- compared to Mut23-F-DGCR8-expressing cells, the average and standard error (n=3) of the $\log_2$ relative expression in Mim23- compared to WT-F-DGCR8-expressing cells.

Table S6. 534 MiRNAs that Show Less than a 2-fold Change in the Mim23-Expressing Cells Relative to Either WT- and/or Mut23-Expressing Cells; Related to Figure 5. The table shows the miRNA id, the sum of reads from all samples analyzed that mapped to this miRNA, the precursor id corresponding to the mature miRNA, the average and standard error (n=3) of the $\log_2$ relative expression in Mim23- compared to Mut23-F-DGCR8- expressing cells, the average and standard error (n=3) of the $\log_2$ relative expression in Mim23- compared to WT-F-DGCR8-expressing cells.

SUPPLEMENTAL EXPERIMENTAL PROCEDURES

Plasmids

pFLAG/HA-DGCR8 (pFH-DGCR8) and pcDNA4/TO/cmycDrosha (Landthaler et al., 2004) were purchased (Addgene) and used to clone pCS3-MT-MycDrosha, all wildtype, mutant and mimetic FH-DGCR8 vectors, pSNAP-DGCR8 (all for transient transfections), pcDNA5/FRT-F-DGCR8 (for stable transfections), pET28a-DGCR8 (for bacterial expression), and pFast-Bac1-HisDGCR8 (for baculovirus expression). A Myc-Drosha PCR fragment was inserted into the XbaI site of the pCS3-MT plasmid to create pCS3-MT-MycDrosha. DGCR8 mutant and mimetic constructs were created with QuikChange multi- and single-site directed mutagenesis kits (Stratagene). To create pcDNA5/FRT-FLAG-DGCR8 plasmids, the HA tags in all versions of pFH-DGCR8 were deleted using site-directed mutagenesis and plasmids were digested with KpnI and NotI to obtain FLAG-DGCR8 inserts, which were subsequently ligated to the pCDNA5/FRT vector. DGCR8 PCR fragments were inserted into the NotI site of pSNAP-tag(m) vector (NEB) to create the pSNAP-DGCR8. pET28a-DGCR8 was cloned by inserting the DGCR8 PCR fragment between the EcoRI and HindIII sites of pET28a(+) (Novagen). His-tagged DGCR8 was excised from pET-28a-DGCR8 using the XbaI and HindIII sites and ligated into the same sites of pFast-Bac1 (Invitrogen) to give pFast-Bac1-HisDGCR8. pGFPmax was transfected for two reasons: (1) it allowed determination of transfection efficiency, and (2) it provided a loading control for the Northern blots. pcDNA3 was used as the empty vector control.

Mammalian Cell Assays

All mammalian cells (HEK293, HEK293T, HeLa) were cultured as in (Pawlicki and Steitz, 2008). Transfections were performed with TransIT-293 or HeLaMonster reagent (Mirus Bio) per manufacturer's instructions. Cells were harvested 30-48 hr later and frozen on dry ice for at least 30 min until further processing. Cells were lysed on ice for 45 min in lysis buffer [2% NP-40, 10% glycerol, 150 mM NaCl, 50 mM Tris-HCl pH 7.5, 5 mM EDTA, supplemented 1:100 with phosphatase inhibitor cocktails 1 and 2

(Sigma; Sigma later replaced cocktail 1 with 3) and Complete EDTA-free protease inhibitor tablets (Roche)] and spun 15 min at 15K g. Coomassie Plus (Bradford) Protein Assays (Thermo Scientific) were performed to ensure equal loading of lysates onto gels or onto resin for immunoprecipitation.

For metabolic labeling of cells, plates were washed 5 times with phosphate free DMEM (Gibco) and then incubated 3.5 hr in phosphate-free DMEM supplemented with $^{32}PO_4$ (PerkinElmer). Cells were treated with fresh 100 μg/mL cycloheximide (Sigma 100 mg/mL stock dissolved in EtOH), 20 μM U0126 (MEK1/2 inhibitor), 20 μM SP600125 (JNK inhibitor) (LC Labs), or 100 nM Calyculin A (Cell Signaling, LC labs, and Sigma) for ~20 min.

Stable HeLa Flp-In cell lines were created using a Flipase (Flp)/Flp recognition target site-directed recombination system (Invitrogen). A parent cell line was made by infecting HeLa(JW36) cells at low MOI (less than 5%) with a pTYF-based lentiviral construct containing (in succession): EGFP (expressed from EF-1α promoter and followed by the BGH polyadenylation site), the SV40 promoter upstream of an ATG-containing FRT site, and puromycin N-acetyl-transferase (expressed from the PGK promoter). Single EGFP-expressing colonies were selected for two weeks in media containing 0.2 μg/ml puromycin; this parent line yielded the strain 1 HeLa cells. The strain 2 parent line was made similarly, but did not express EGFP and was selected for Zeocin resistance. Isogenic cell lines expressing WT-, Mut23-, and Mim23-F-DGCR8 were produced from each parent Flp-In HeLa line by cotransfecting them with pcDNA5/FRT-FLAG-DGCR8 vectors and pOG44. Stable clones were selected using 200 μg/mL hygromycin (EMD-Millipore). For proliferation assays, strain 2 isogenic HeLa Flp-In cells stably expressing WT-, Mut23-, or Mim23-F-DGCR8 were plated at 200 cells per well in a 96-well plate. After settling overnight, cells were serum starved 24 hr and after serum addition cell proliferation was measured every 24 hr for 5 days using Cell Titer Glo reagent (Promega). Luminescence signals were recorded on a GloMax-Multi+ Plate reader (Promega). For *in vitro* scratch assays, strain 2 isogenic HeLa Flp-In cells stably expressing

WT-, Mut23-, or Mim23-F-DGCR8 were plated at 500,000 cells per 10-cm plate. After adhering overnight, cells were serum starved overnight and then a 200 μL pipette was used to create a scratch before the readdition of serum. Cells were photographed every 12 hr.

Immunochemistry

Equivalent amounts of each lysate were incubated for 2 hr at 4°C with pre-washed anti-FLAG M2 affinity resin (Sigma). Bound-resin was washed 4 times with wash buffer [10% glycerol, 250 mM NaCl, 50 mM Tris-HCl pH 7.5, and 2 mM EDTA supplemented 1:100 with phosphatase inhibitor cocktails 1 and 2 (Sigma) and Complete EDTA-free protease inhibitor tablets (Roche)]. Proteins for processing assays were eluted in wash buffer plus 50 μg/mL 3x FLAG peptide (Sigma). Immunoprecipitations for assessing co-purifying factors were washed with 500 mM NaCl wash buffer to reduce background and then diluted directly into SDS-PAGE loading buffer. Immunoprecipitates of metabolically-labeled cells were washed with 500 mM NaCl wash. Phosphatase treatment was performed by resuspending the washed MC-containing resin in 1 X λ phosphatase buffer, 1 mM $MnCl_2$, and 800 Units of λ Phosphatase (NEB). Samples were incubated at 30°C for 10 min and then diluted directly into SDS-PAGE loading buffer.

Immunoblots were performed according to (Pimienta et al., 2011) using 7.5–15 μg from each lysate. Blots were developed using ECL reagents (Thermo Scientific Femto Maximum Sensitivity Substrate or PerkinElmer Western Lightning ECL), images acquired on the Syngene G:Box Chemi XT[4] System, and quantitated using the GeneSys software. Immunofluorescence was performed according to (Pawlicki and Steitz, 2008) with primary antibodies diluted 1:100 and images acquired on a Leica TCS SP5 confocal microscope. The following antibodies were used: anti-DGCR8 (ProteinTech), anti-FLAG (Sigma F3165 for blots and F1804 for fluorescence), anti-Drosha, anti-GAPDH, anti-Dicer, anti-phospho-(Ser/Thr) MAPK/CDK substrate antibody sampler kit, MAPK antibody sampler kit, phospho-MAPK antibody sampler kit (Cell Signaling), anti-DDX5, anti-DDX17, anti-TRBP (Abcam), anti-Ago2 (Millipore), goat-anti-

mouse-Alexa488, goat-anti-rabbit-Alexa594 (Invitrogen), goat anti-mouse-HRP, and goat-anti-rabbit-HRP (Thermo Scientific).

Northern Blots

Northern blots were performed according to (Pawlicki and Steitz, 2008) using 7.5 μg RNA from each sample. Membranes were hybridized in ExpressHyb Solution (Clontech) overnight at 42°C to oligonucleotide probes end-labeled with T4 polynucleotide kinase in the presence of [γ-^{32}P]ATP. Probe sequences are shown below.

	DNA sequence (5′ to 3′)
GFP probe	CGTACTTCTCGATGCGGGTGTTGG
Myc probe	CAAGTCCTCTTCAGAAATGAGCTTTTGCTC
FLAG probe	CTTGTCATCGTCGTCCTTCTAGTCCAT

In Vitro Processing Assays

In vitro processing assays were performed according to (Pawlicki and Steitz, 2008) with MCs purified by anti-FLAG immunoprecipitation. Pri-miRNA encoding plasmids were cloned as in (Pawlicki and Steitz, 2008). Pri-miR16-2 *in vitro* transcription templates were generated by PCR on pcDNA3-pri-miR-15b~16-2 using a primer that adds a T7 RNA polymerase promoter. Other *in vitro* transcription templates were created by PCR with CMV and SP6 primers. *In vitro* transcription templates for generating the loading control were created by digesting pcDNA3 with BamHI and fill-in with T4 DNA polymerase. Approximately 1.25×10^5 cpm (~1 fmol) of *in vitro* transcribed pri-miRNA substrate and 1.5×10^4 cpm of the loading control were used per processing reaction. Reactions proceeded for 45 min at 37°C.

MiRNA Profiling

RNA from 10 cm plates of isogenic Flp-In HeLa cells expressing Mim23-, Mut23-, or WT-F-DGCR8 was prepared by Trizol (Invitrogen) extraction and subsequent Qiagen RNeasy cleanup procedure with on-column DNase treatment. RNA from 3 biological replicates was submitted to the Keck Yale Center for Genome Analysis for TruSeq small RNA library preparation and Illumina HiSeq 2000 sequencing. Data were analyzed using miRDeep2 (Friedlander et al., 2012). Reads were mapped to the human genome, Hg19. The quantifier module normalizes reads per mature miRNA to the total number of mature miRNA reads in each sample. For TaqMan quantitative PCR analysis of miRNA expression, cDNA was created and qPCRs were done using the TaqMan MicroRNA Reverse Transcription Kit, TaqMan Universal PCR Master Mix, No AmpErase UNG and individual miRNA assays [Applied Biosystems: hsa-miR-21 (ID: 000397), hsa-miR-181a (ID: 000480), hsa-let-7c (ID: 000379), hsa-miR-93 (ID: 000432), RNU6B (ID: 001093)] according to the manufacturer's instructions.

Search for MAPK Docking Motifs

A custom Perl script was written to search for instances of D-sites, as defined by (Garai et al., 2012), in a given protein amino acid sequence. The regular expressions [KR]{1,3}.{3,7}[ILVM].[ILVF] (General), [RK][P]..[LIV].[LIVMPF] (pepJIP type), [RK]..[LIVMP].[LIV].[LIVMPF] (pepNFAT4 type), [RK].{3,4}[LIVMP].[LIV].[LIVMPFA] (pepMKK6 type), [LI]..[RK][RK].{5}[LIVMP].[LIV].[LIVMPFA] (pepHePTP type), and [LIVMPFA].[LIV].{1,2}[LIVMP].{4,6}[LI]..[RK][RK] (RSK/MAPKAP type) were used.

In vitro Kinase Assays

HA-ERK2 (pcDNA3-HA-ERK2 WT) and MKK1 plasmids (pMCL-HA-MAPKK1-R4F [delta(31-51)/S218E/S222D] and pMCL-HA-MAPKK1-8E (K97M)) were the kind gift of B. Turk. FLAG-JNK plasmids (pCDNA3 Flag Jnk1a1, pCDNA3 Flag Jnk1a1(apf), pCDNA3 Flag MKK7B2Jnk1a1, pCDNA3 Flag MKK7B2Jnk1a1(apf)) were purchased from Addgene. Kinases were immunoprecipitated from HEK293T cells as described above, except that two additional washes were done in kinase buffer (50 mM Tris-HCl,

pH 7.5, 25mM $MgCl_2$, 150mM NaCl, 10% Glycerol). FLAG-JNKs were either eluted using a 3X FLAG-peptide in kinase buffer for the fixed substrate concentration experiments or the resin was resuspended in an equal volume of kinase buffer for the fixed kinase concentration experiments. HA-ERK constructs were immunoprecipitated as above except that lysates were incubated for 2 hr with HA.11 Clone 16B12 Monoclonal Antibody ascites (Covance) and then 25uL of a 50% slurry of protein A sepharose CL-48 (GE Healthcare) for another hr at 4°C. After washing, the sepharose was resuspended in an equal volume of Kinase buffer. Immunoprecipitated kinases were incubated with bacterially expressed DGCR8 in Kinase buffer plus 1mM DTT, 20 μM ATP, and 10 μCi [γ-^{32}P]ATP for 30 min at 30°C, after which reactions were quenched by adding NuPAGe loading dye (Invitrogen) and electrophoresed through gradient Bis-Tris polyacrylamide gels (Invitrogen).

Analysis

^{32}P-containing gels or membranes were imaged using a Storm PhosphorImager (Molecular Dynamics). Data were plotted and fit in Igor (Wavemetrics).

Proteomics Sample Preparation

Bacmids were prepared from pFast-Bac1-HisDGCR8 in DH10BAC *E. coli*, according to the manufacturer's protocol (Invitrogen). Sf9 cells were grown at 27°C in Grace's media (Gibco) supplemented with 2 mM L-Glut, 1 X penicillin streptomycin solution (Sigma Aldrich), 0.25 μg/ml Amphotericin B solution (Sigma), and 10% fetal bovine serum (BD Biosciences) and transfected with bacmids using Cellfectin (Invitrogen). After 3 days, viral stocks were isolated and amplified. Hi-5 cells were grown at 27°C in shaker flasks in SfII900 medium (Gibco) supplemented as above. One hundred twenty-five mLs of Hi-5 cells at 2 X 10^6 cells/mL were infected with 2.5 mLs of P2 viral stock and grown at 27°C for 48 hr, harvested and frozen. Cells were lysed in 50 mL Buffer A (50 mM NaH_2PO_4 pH 8.0, 300 mM NaCl, and 20 mM imidazole) supplemented with 1% NP-40 and Complete EDTA-free protease inhibitor tablets (Roche) on ice for 30

min, and then spun at 30K relative centrifugal force for 30 min. Lysates were applied to a 5 mL HisTrap (GE) column, washed with 4 column volumes Buffer A and eluted with a 1 hr linear gradient of Buffer A to Buffer B (Buffer A plus 250 mM Imidazole). Fractions containing DGCR8 were pooled and concentrated using a 50 kDa cut-off Amicon filter (Millipore).

Affinity-purified DGCR8 samples were separated by SDS-PAGE. Several Coomassie-stained DGCR8 bands were cut and in-gel digested with trypsin (Shevchenko et al., 2006). For samples prepared from mammalian cells, peptides were subsequently captured and eluted step-wise from a strong cation exchange matrix (SCX). The SCX PolySULFOETHYLA (PolyLC) matrix was equilibrated with loading buffer (50mM sodium citrate, pH 2.5, and 2.5% acetonitrile). Bound peptides were eluted in each step with 2 washes of loading buffer adjusted with 25% ammonium hydroxide to six different pH values (3, 4, 5, 6, 7, and 10). The SCX peptide fractions were desalted by C18-based solid phase extraction (C18 zip-tip, Agilent) and dried by vacuum centrifugation.

Phosphopeptides were captured batch-wise with TiO_2 either directly from the in-gel trypsin digestion or following SCX pH fractionation. After 3 washes with loading buffer (90% acetonitrile, 5% TFA, 5% water), the captured phosphopeptides, were eluted twice with 25% ammonium hydroxide and immediately mixed with Tris base solution pH 8.5. Samples were desalted and dried as described above. Peptides from the insect samples that did not bind TiO_2 (supernatant) were further processed for any missed phosphopeptides by capturing them batch-wise on PhosphoSelect IMAC (Sigma-Aldrich) beads in loading buffer (30% acetic acid, 30% acetonitrile, 30% water). Captured peptides were eluted, desalted, and dried using the TiO_2 strategy described above. For peptide and phosphosite identification, each peptide fraction was further separated by reversed-phase (RP) liquid chromatography (LC) coupled online to a tandem mass spectrometer. This was performed at the Yale University Keck Proteomics Facility, using LTQ Orbitrap Elite coupled to a Waters nanoACQUITY Ultra High Performance Liquid

Chromatography (UPLC) system that uses a Waters Symmetry C18 180 µm x 20 mm trap column and a prepacked 1.7 µm, 75 µm x 250 mm nanoACQUITY UPLC column (35°C) for peptide separation.

SUPPLEMENTAL REFERENCES

Cox, J., Neuhauser, N., Michalski, A., Scheltema, R.A., Olsen, J.V., and Mann, M. (2011). Andromeda: a peptide search engine integrated into the MaxQuant environment. J Proteome Res *10*, 1794-1805.

Friedlander, M.R., Mackowiak, S.D., Li, N., Chen, W., and Rajewsky, N. (2012). miRDeep2 accurately identifies known and hundreds of novel microRNA genes in seven animal clades. Nucleic Acids Res *40*, 37-52.

Garai, A., Zeke, A., Gogl, G., Toro, I., Fordos, F., Blankenburg, H., Barkai, T., Varga, J., Alexa, A., Emig, D., *et al.* (2012). Specificity of linear motifs that bind to a common mitogen-activated protein kinase docking groove. Sci Signal *5*, ra74.

Gnad, F., Gunawardena, J., and Mann, M. (2011). PHOSIDA 2011: the posttranslational modification database. Nucleic Acids Res *39*, D253-260.

Gnad, F., Ren, S., Cox, J., Olsen, J.V., Macek, B., Oroshi, M., and Mann, M. (2007). PHOSIDA (phosphorylation site database): management, structural and evolutionary investigation, and prediction of phosphosites. Genome Biol *8*, R250.

Landthaler, M., Yalcin, A., and Tuschl, T. (2004). The human DiGeorge syndrome critical region gene 8 and its D. melanogaster homolog are required for miRNA biogenesis. Curr Biol *14*, 2162-2167.

Linding, R., Jensen, L.J., Ostheimer, G.J., van Vugt, M.A., Jorgensen, C., Miron, I.M., Diella, F., Colwill, K., Taylor, L., Elder, K., *et al.* (2007). Systematic discovery of in vivo phosphorylation networks. Cell *129*, 1415-1426.

Pawlicki, J.M., and Steitz, J.A. (2008). Primary microRNA transcript retention at sites of transcription leads to enhanced microRNA production. J Cell Biol *182*, 61-76.

Pimienta, G., Herbert, K.M., and Regan, L. (2011). A compound that inhibits the HOP-Hsp90 complex formation and has unique killing effects in breast cancer cell lines. Mol Pharm *8*, 2252-2261.

Shevchenko, A., Tomas, H., Havlis, J., Olsen, J.V., and Mann, M. (2006). In-gel digestion for mass spectrometric characterization of proteins and proteomes. Nat Protoc *1*, 2856-2860.
