# Supplementary material for: Phosphorylation of DGCR8 Increases Its Intracellular Stability and Induces a Progrowth miRNA Profile
Source: Cell Rep. Author manuscript; Available in PMC 2014 Nov 27. (PMC3892995; doi:10.1016/j.celrep.2013.10.017)

pSer35

Protein:	Q8WYQ5-1; Q8WYQ5; Q53GY5; Q8WYQ5-3; Q8WYQ5; Q8WYQ5-2; Q8WYQ5; C9JSD5
Score:	88.29
Source:	OT11-1423
Scannumber:	7475
Method:	ITMS; CID; 2

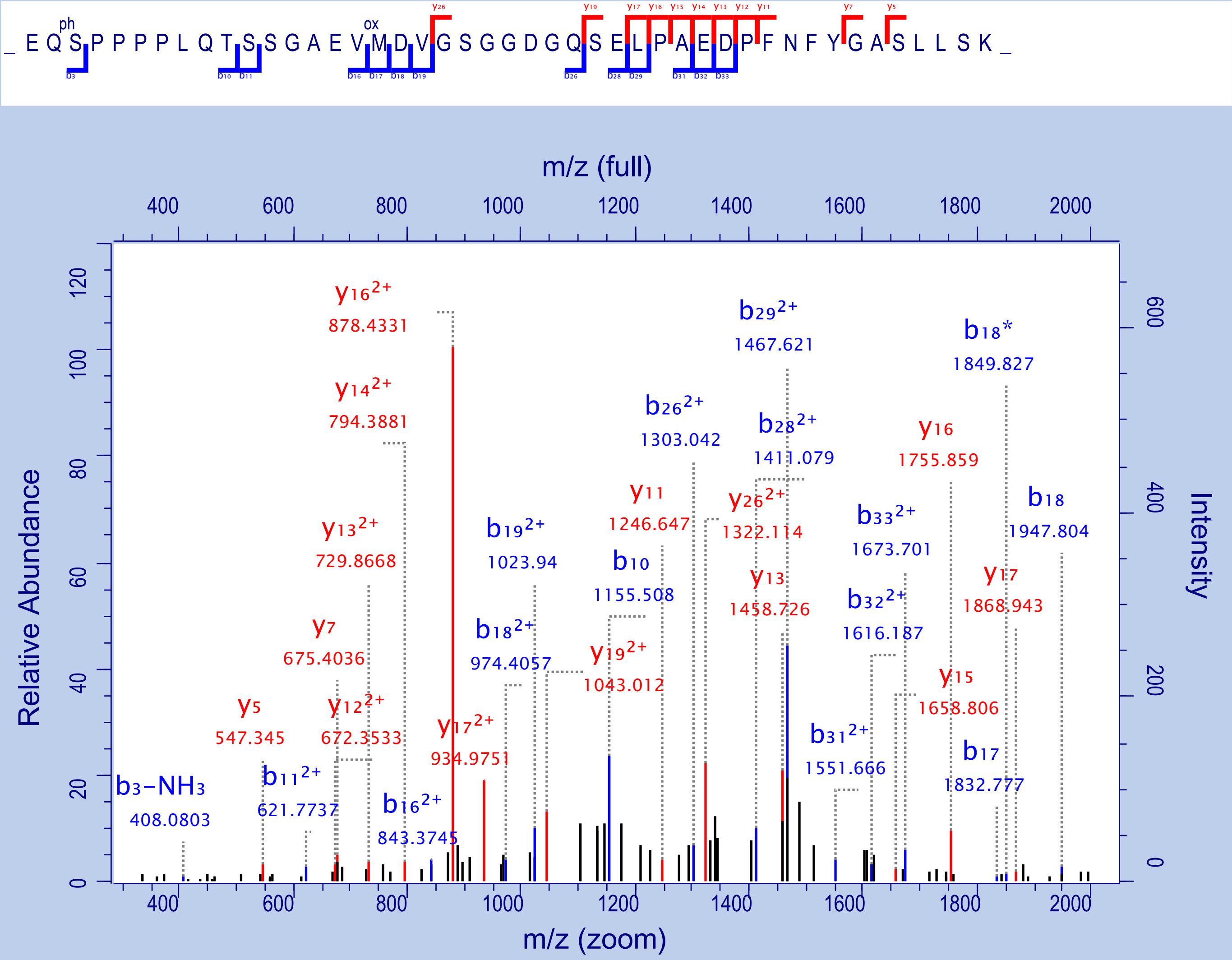

pSer35

Protein: Q8WYQ5-1; Q8WYQ5; Q53GY5; Q8WYQ5-3; Q8WYQ5; Q8WYQ5-2; Q8WYQ5; C9JSD5
Score: 112.75
Source: OT10-0371_TiO2El
Scannumber: 4503
Method: ITMS; CID; 2

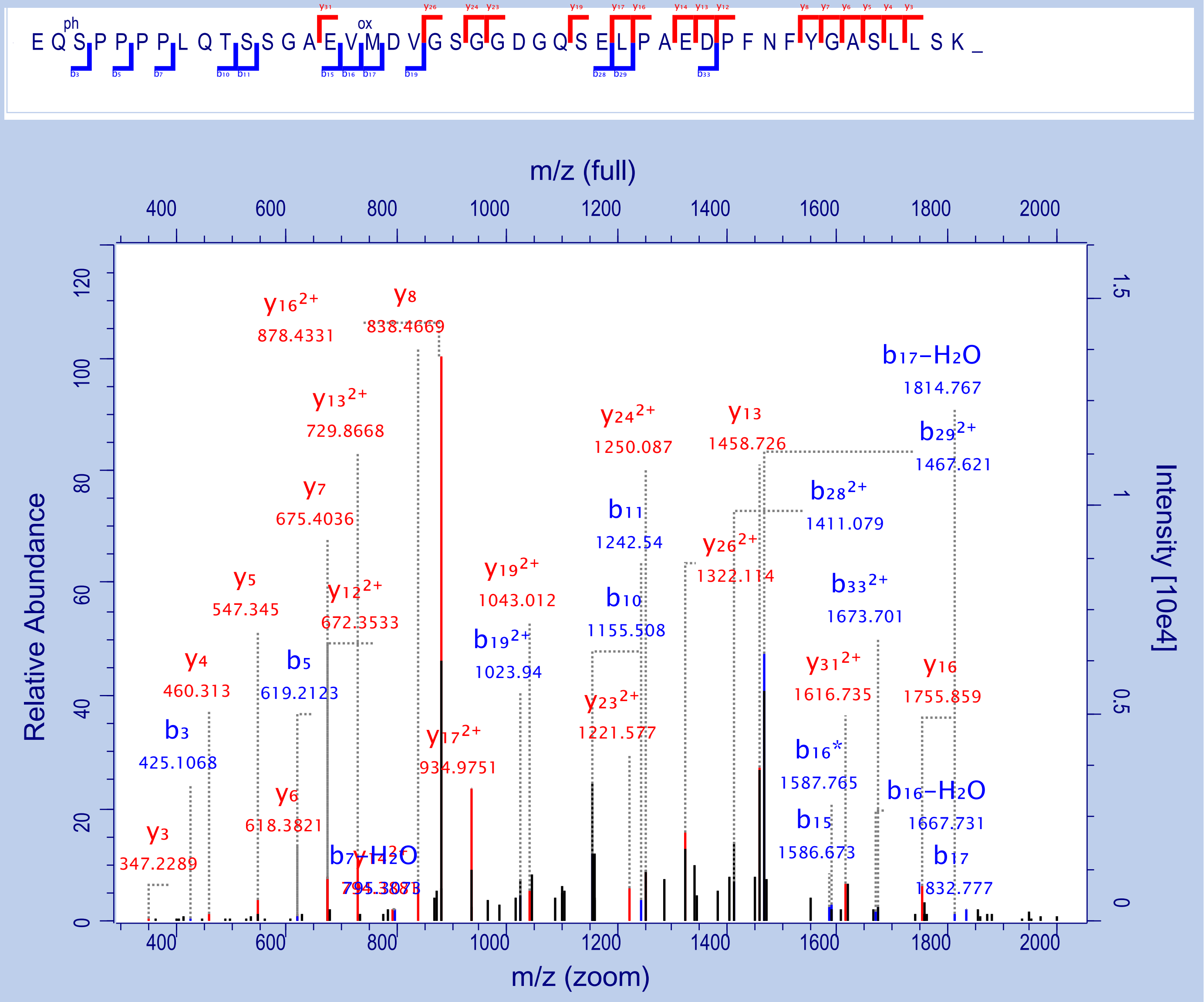

pT42

Protein:	Q8WYQ5-1; Q8WYQ5; Q53GY5; Q8WYQ5-3; Q8WYQ5; Q8WYQ5-2; Q8WYQ5; C9JSD5
Score:	158.24
Source:	OT10-0371_TiO2El
Scannumber:	4607
Method:	ITMS; CID; 2

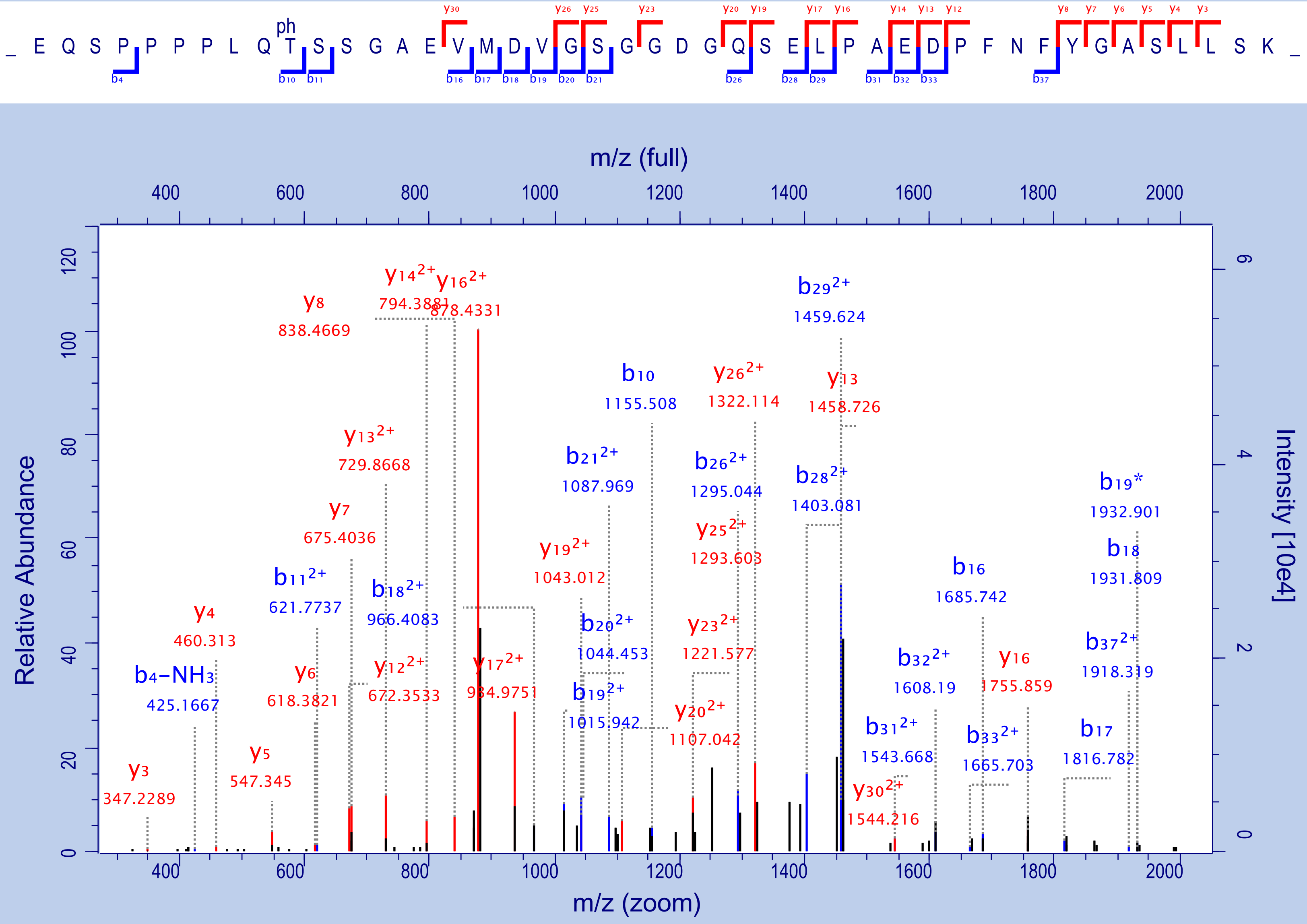

pSer35 and pSer59

Protein: Q8WYQ5-1; Q8WYQ5; Q53GY5; Q8WYQ5-3; Q8WYQ5; Q8WYQ5-2; Q8WYQ5; C9JSD5
Score: 83.43
Source: OT10-0371_TiO2El
Scannumber: 4651
Method: ITMS; CID; 2

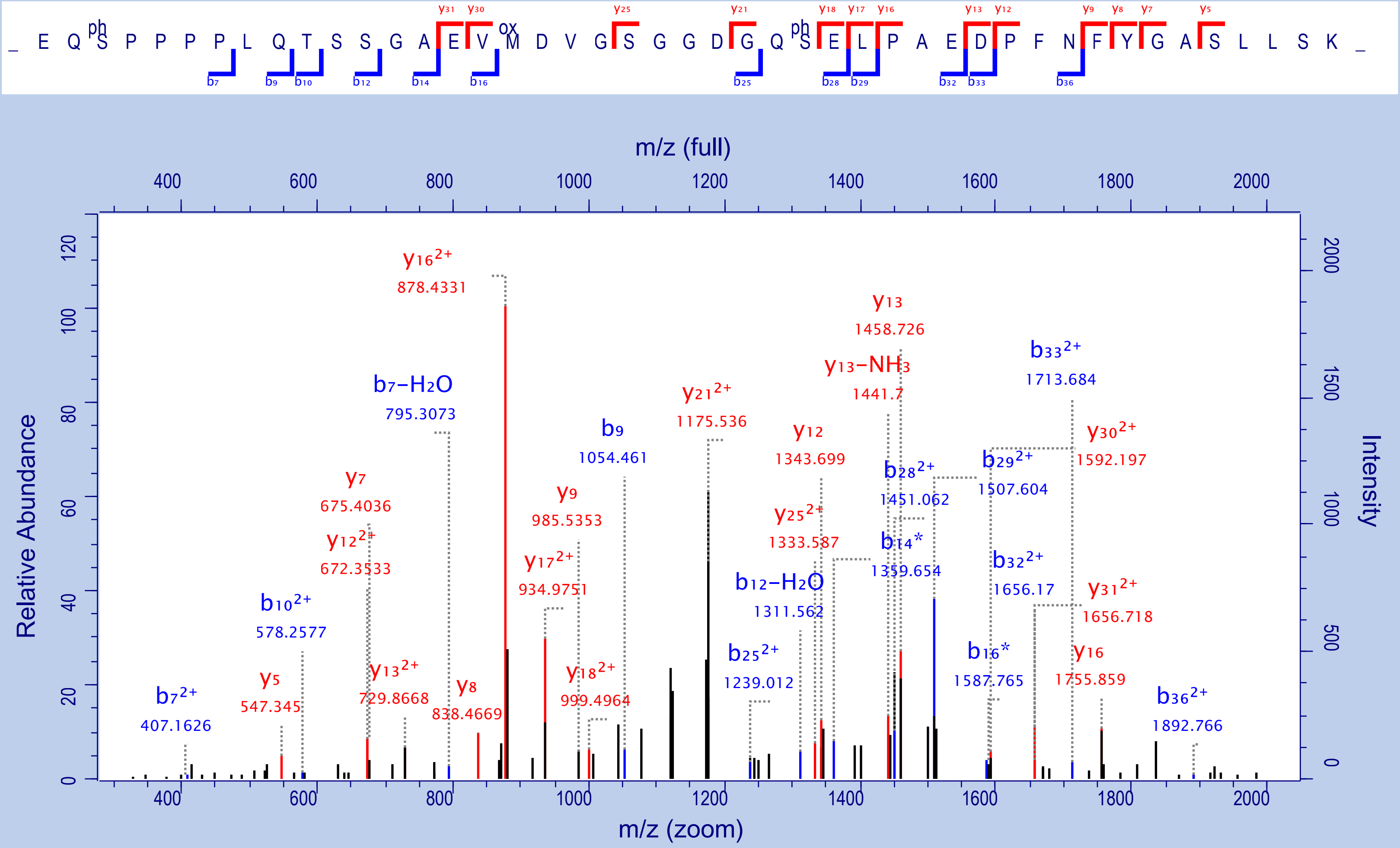

pS92

Protein: Q8WYQ5-1; Q8WYQ5; Q53GY5; Q8WYQ5-3; Q8WYQ5; Q8WYQ5-2; Q8WYQ5; C9JSD5
Score: 25.64
Source: OT10-0371_TiO2El
Scannumber: 2959
Method: ITMS; CID; 2

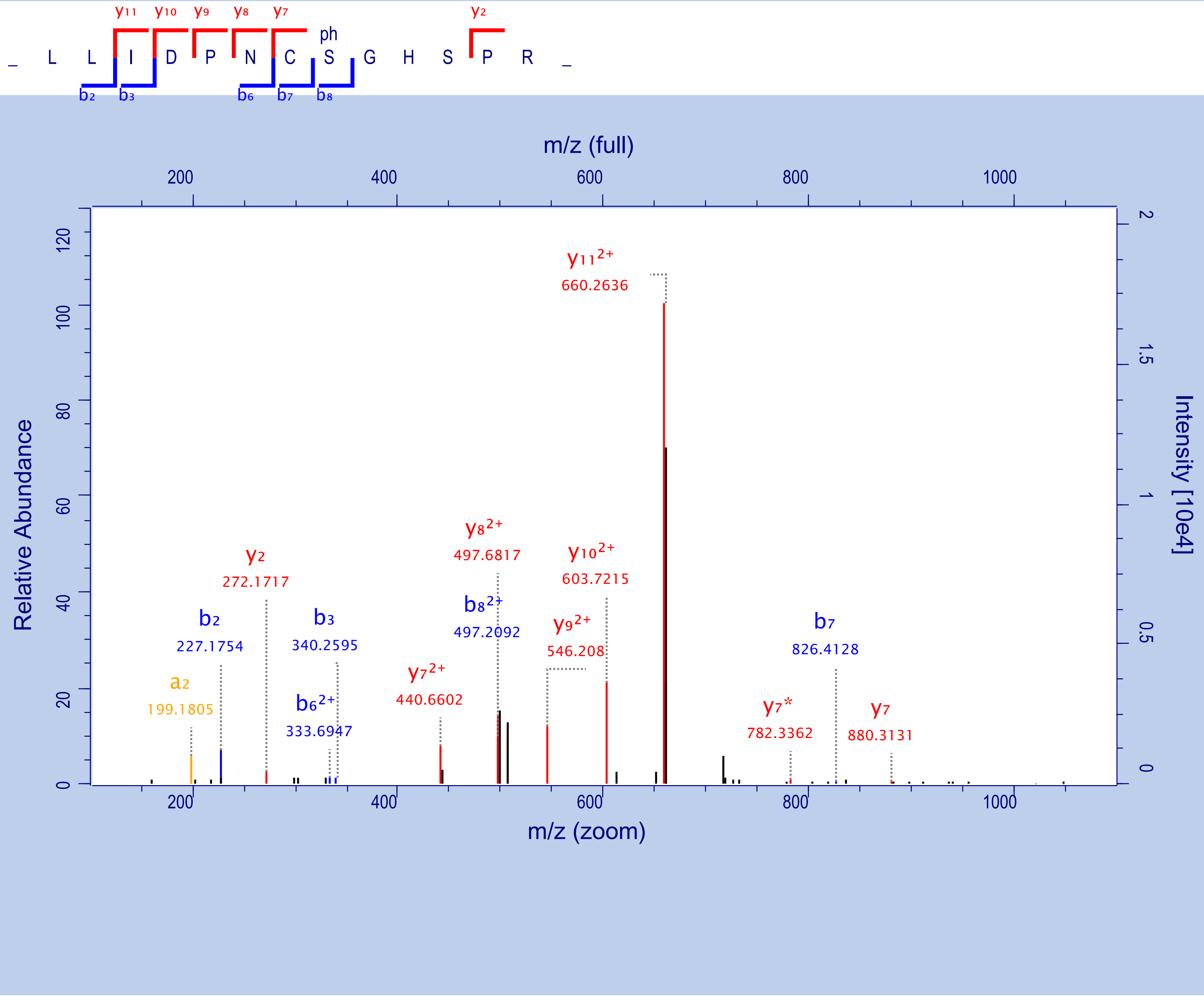

pS95

Protein: Q8WYQ5-1; Q8WYQ5; Q53GY5; Q8WYQ5-3; Q8WYQ5; Q8WYQ5-2; Q8WYQ5; C9JSD5
Score: 72.23
Source: OT10-0371_TiO2El
Scannumber: 2963
Method: ITMS; CID; 2

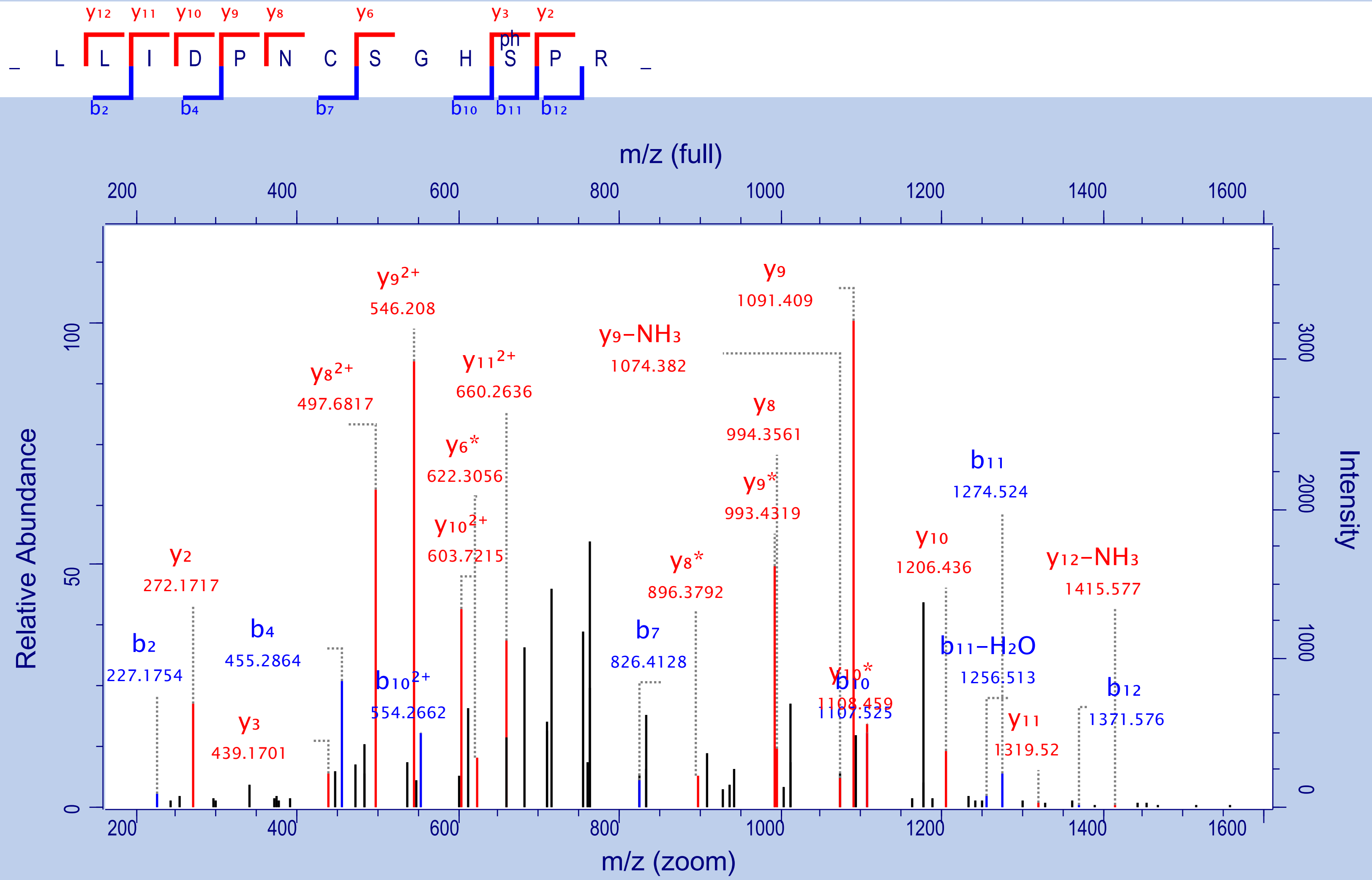

pSer109

Protein: Q8WYQ5-1; Q8WYQ5; Q53GY5; Q8WYQ5-3; Q8WYQ5; Q8WYQ5-2; Q8WYQ5; C9JSD5
Score: 17.87
Source: OT11-1424
Scannumber: 2811
Method: ITMS; CID; 2

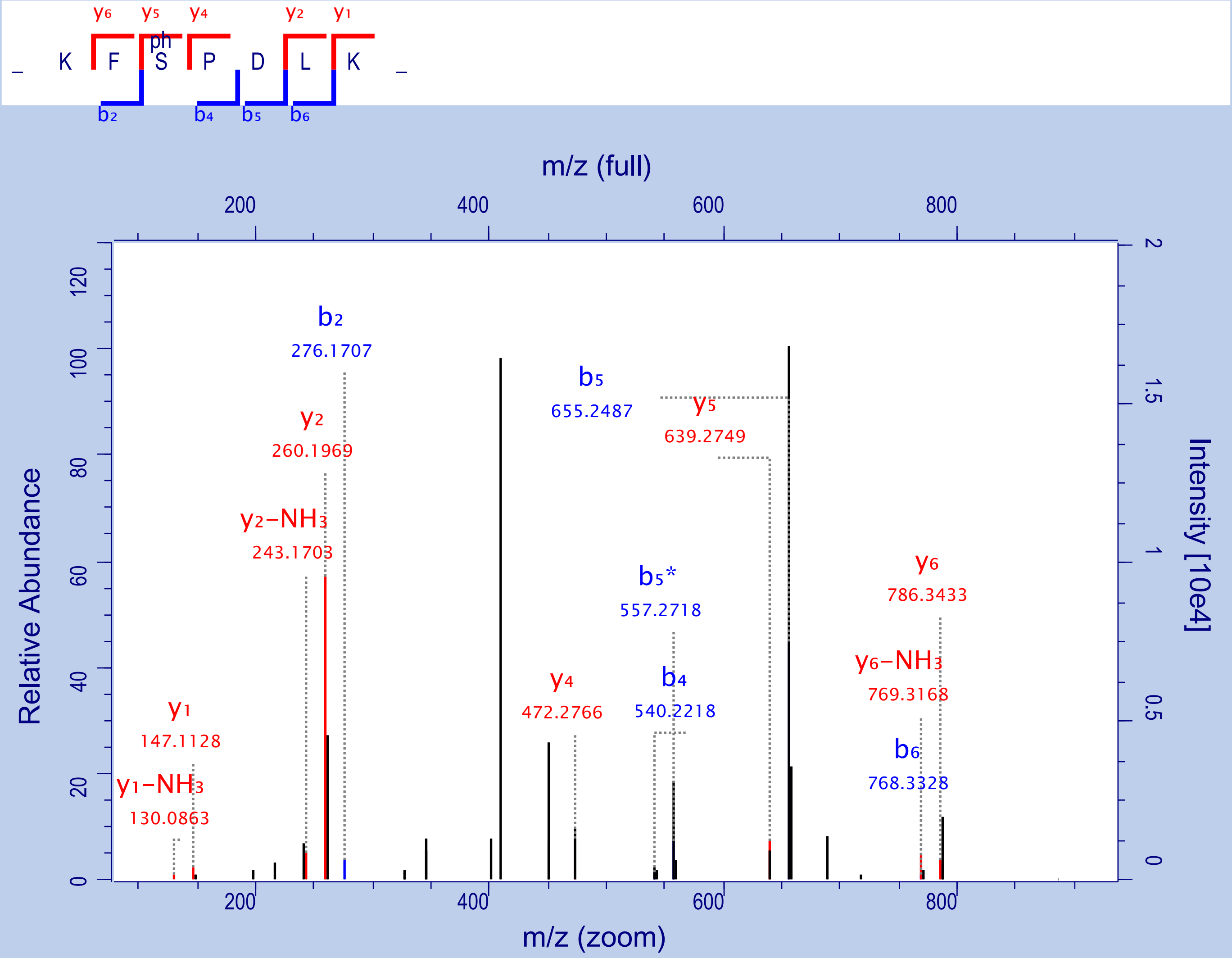

pSer123

Protein:	Q8WYQ5-1; Q8WYQ5; Q53GY5; Q8WYQ5-3; Q8WYQ5; Q8WYQ5-2; Q8WYQ5
Score:	37.01
Source:	VOT11-1093
Scannumber:	2109
Method:	FTMS; HCD; 2

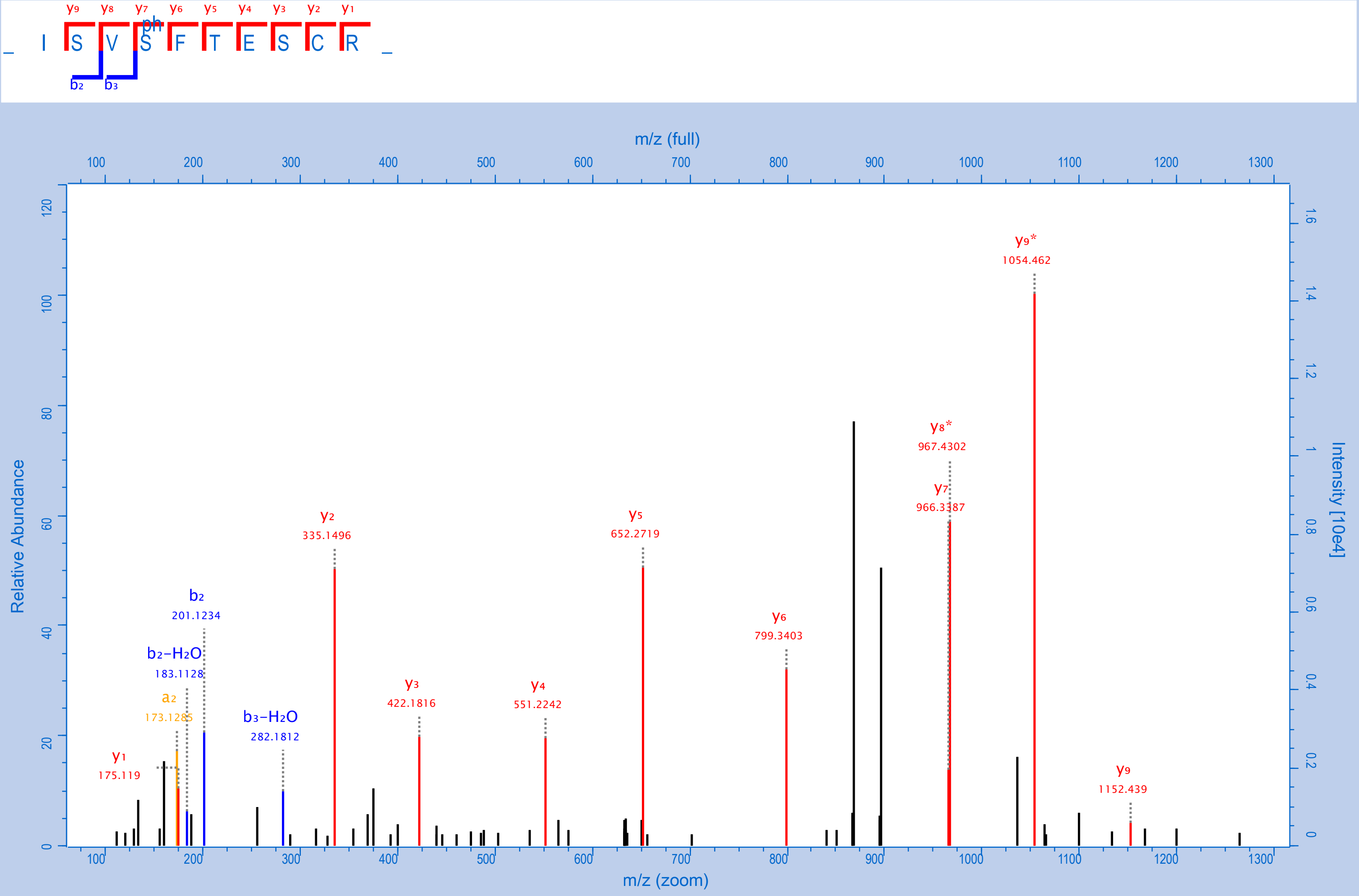

pSer123 and pSer127

Protein: Q8WYQ5-1; Q8WYQ5; Q53GY5; Q8WYQ5-3; Q8WYQ5; Q8WYQ5-2; Q8WYQ5
Score: 39.39
Source: VOT11-1093
Scannumber: 2107
Method: FTMS; HCD; 2

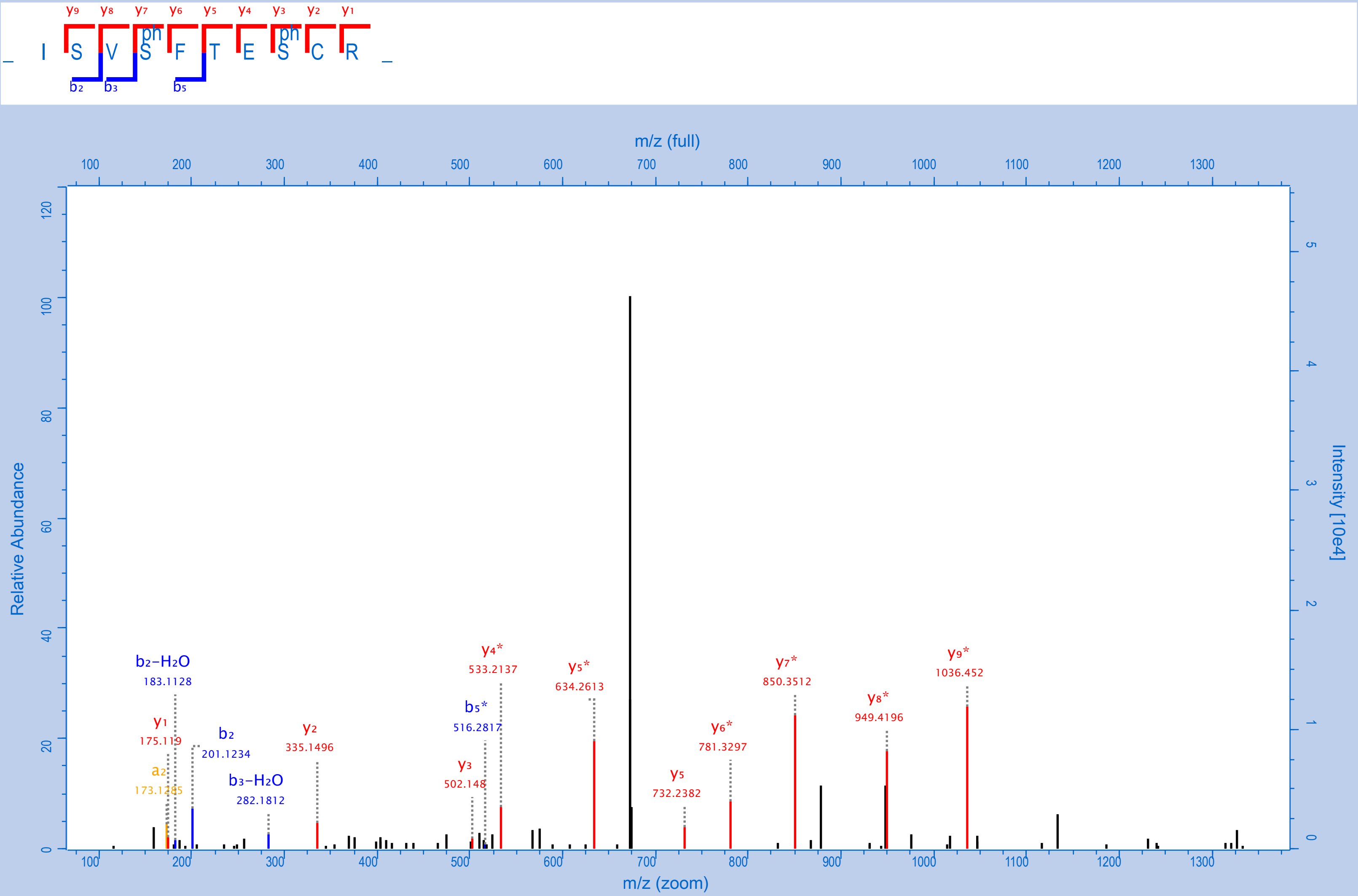

pThr125

Protein:	Q8WYQ5-1; Q8WYQ5; Q53GY5; Q8WYQ5-3; Q8WYQ5; Q8WYQ5-2; Q8WYQ5
Score:	72.11
Source:	VOT11-1093
Scannumber:	2193
Method:	FTMS; HCD; 2

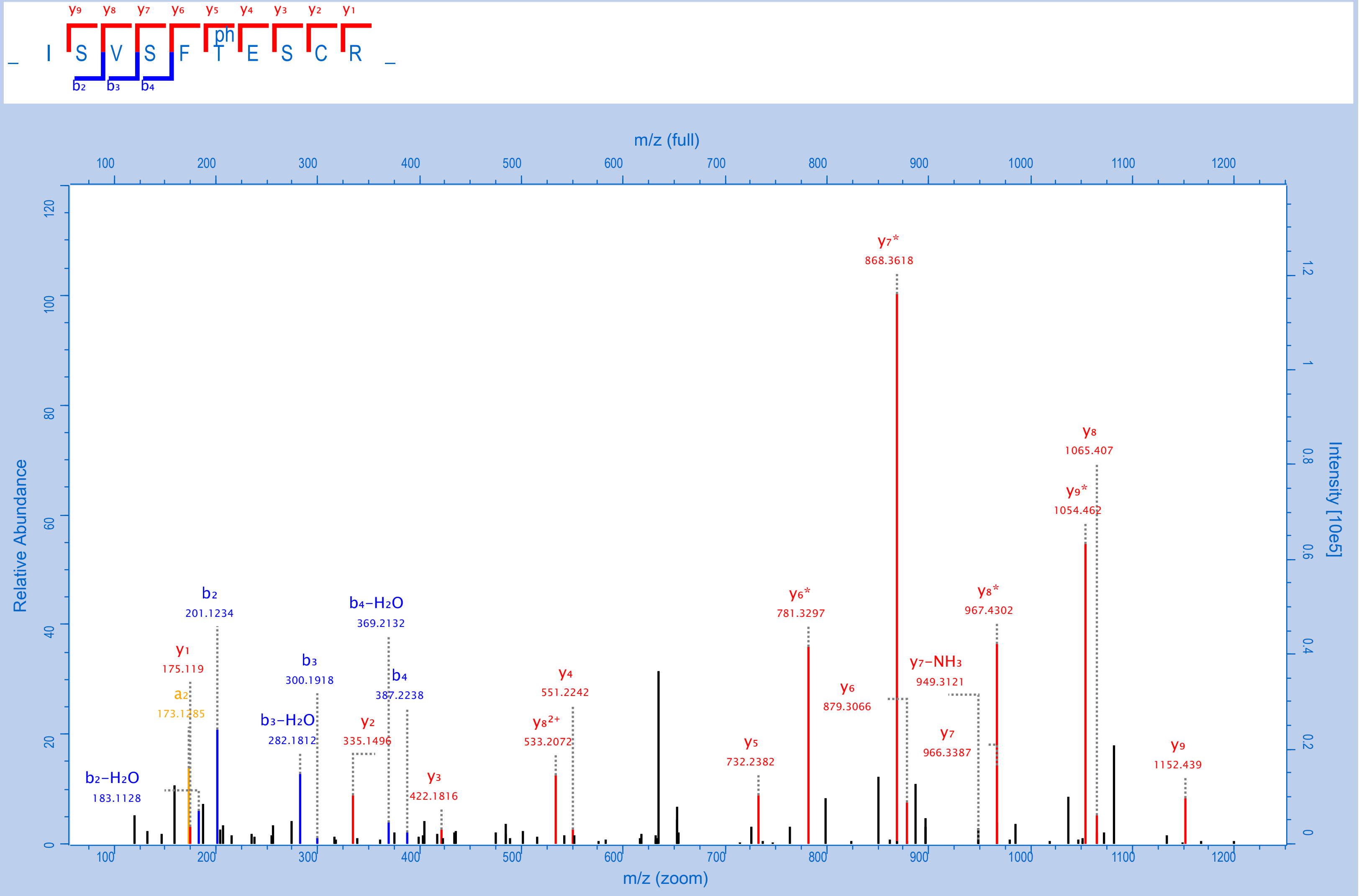

pThr138

Protein: Q8WYQ5-1; Q8WYQ5; Q53GY5; Q8WYQ5-3; Q8WYQ5; Q8WYQ5-2; Q8WYQ5
Score: 28.87
Source: OT11-1424
Scannumber: 2220
Method: ITMS; CID; 2

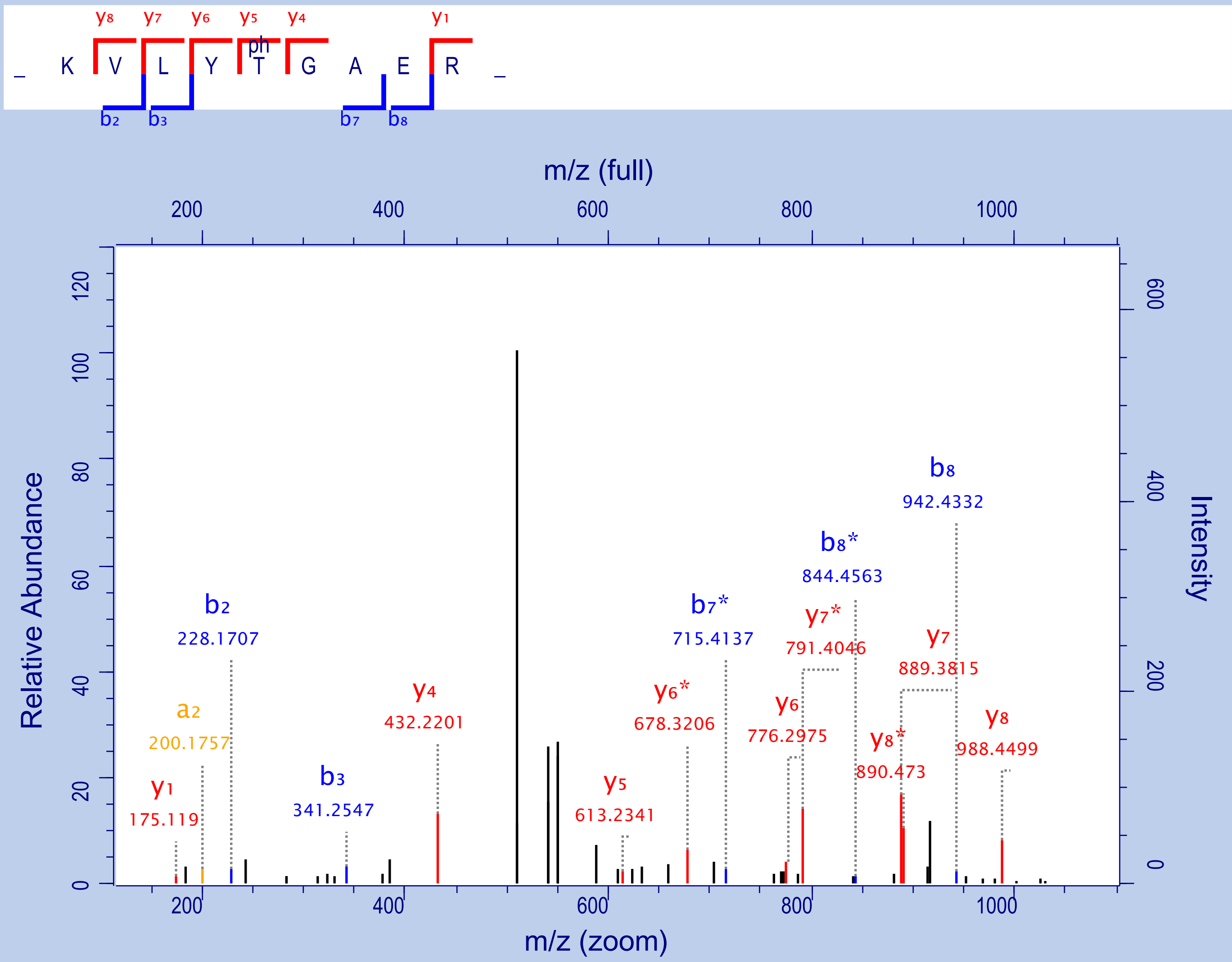

pS153

Protein: Q8WYQ5-1; Q8WYQ5; Q53GY5; Q8WYQ5-3; Q8WYQ5; Q8WYQ5-2; Q8WYQ5
Score: 88.03
Source: OT10-0371_TiO2El
Scannumber: 3794
Method: ITMS; CID; 2

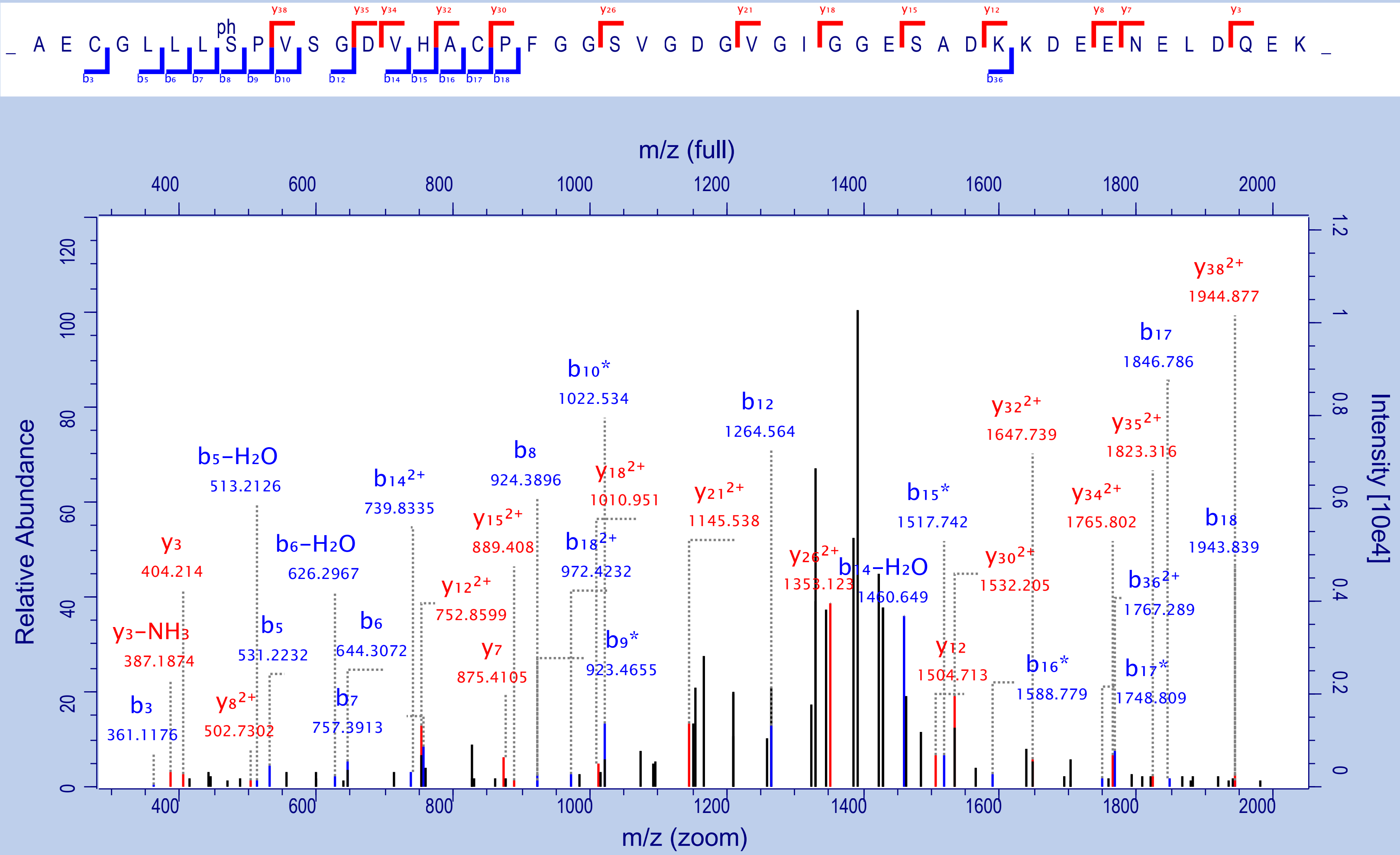

pS156

Protein: Q8WYQ5-1; Q8WYQ5; Q53GY5; Q8WYQ5-3; Q8WYQ5; Q8WYQ5-2; Q8WYQ5
Score: 112.06
Source: OT10-0371_TiO2El
Scannumber: 4005
Method: ITMS; CID; 2

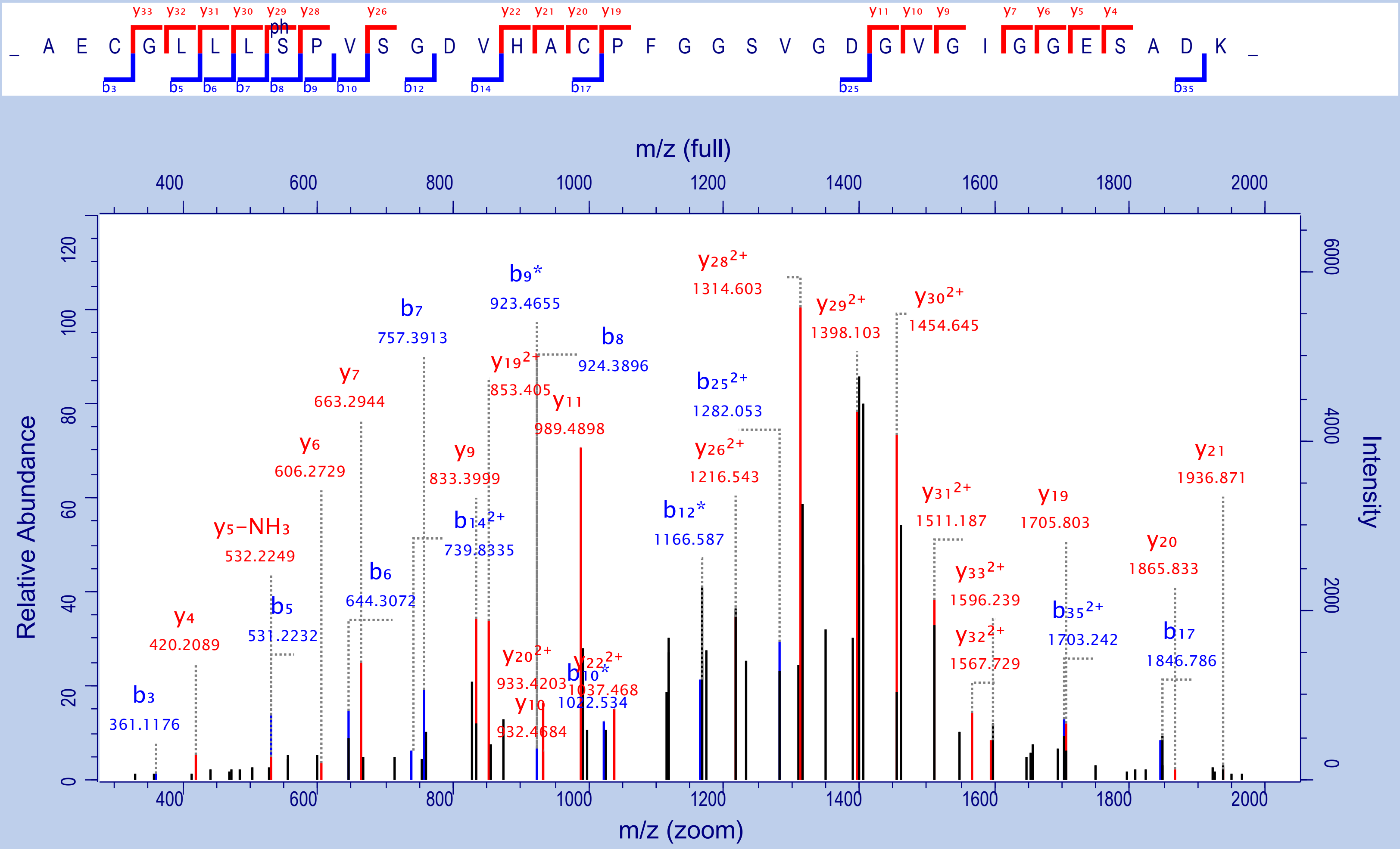

pTyr267 and pSer271

Protein: Q8WYQ5-1; Q8WYQ5; Q53GY5; Q8WYQ5-3; Q8WYQ5; Q8WYQ5-2; Q8WYQ5
Score: 11.05
Source: OT11-1426
Scannumber: 2200
Method: ITMS; CID; 2

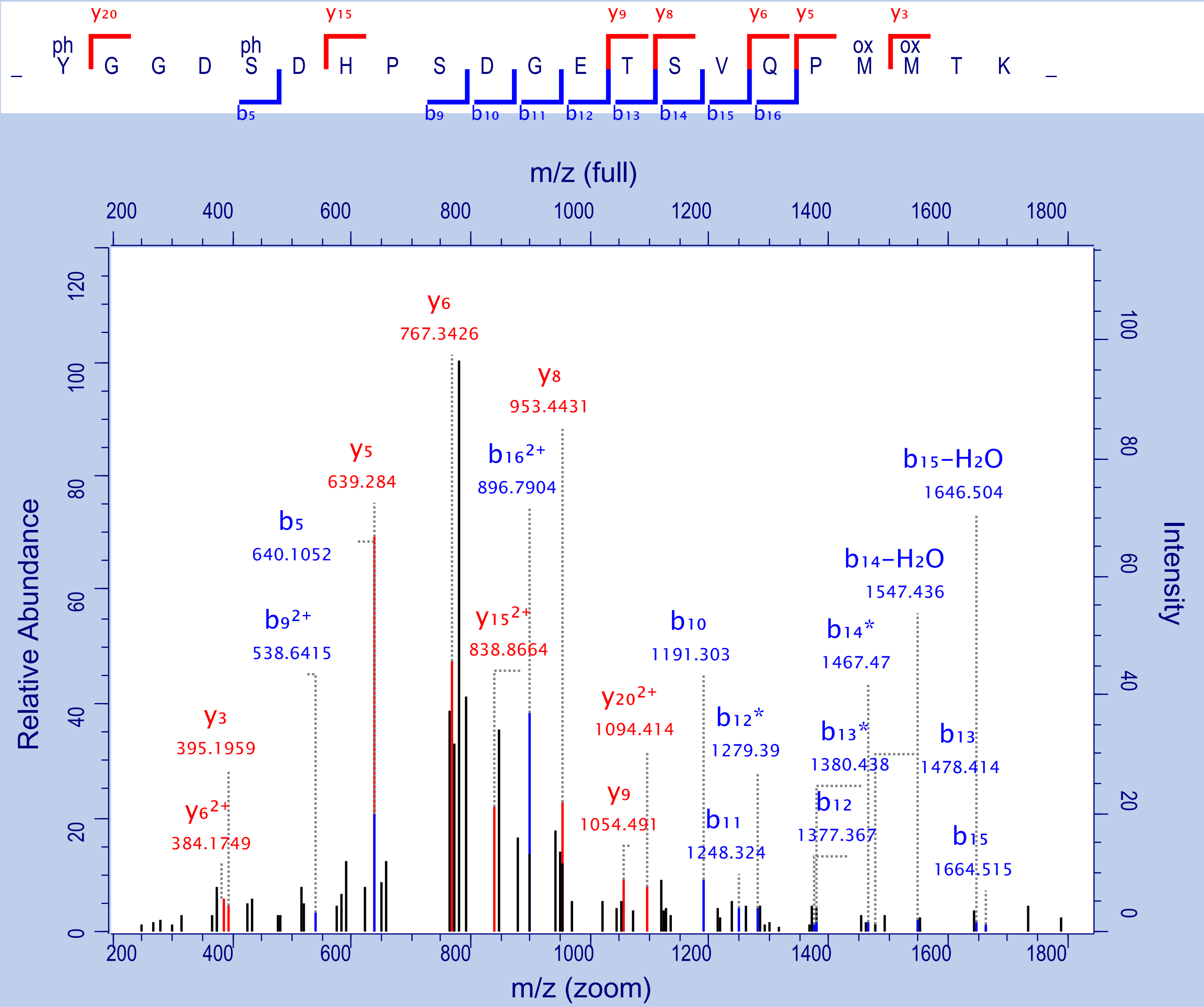

pSer271

Protein: Q8WYQ5-1; Q8WYQ5; Q53GY5; Q8WYQ5-3; Q8WYQ5; Q8WYQ5-2; Q8WYQ5
Score: 13.59
Source: OT11-1419
Scannumber: 2091
Method: ITMS; CID; 2

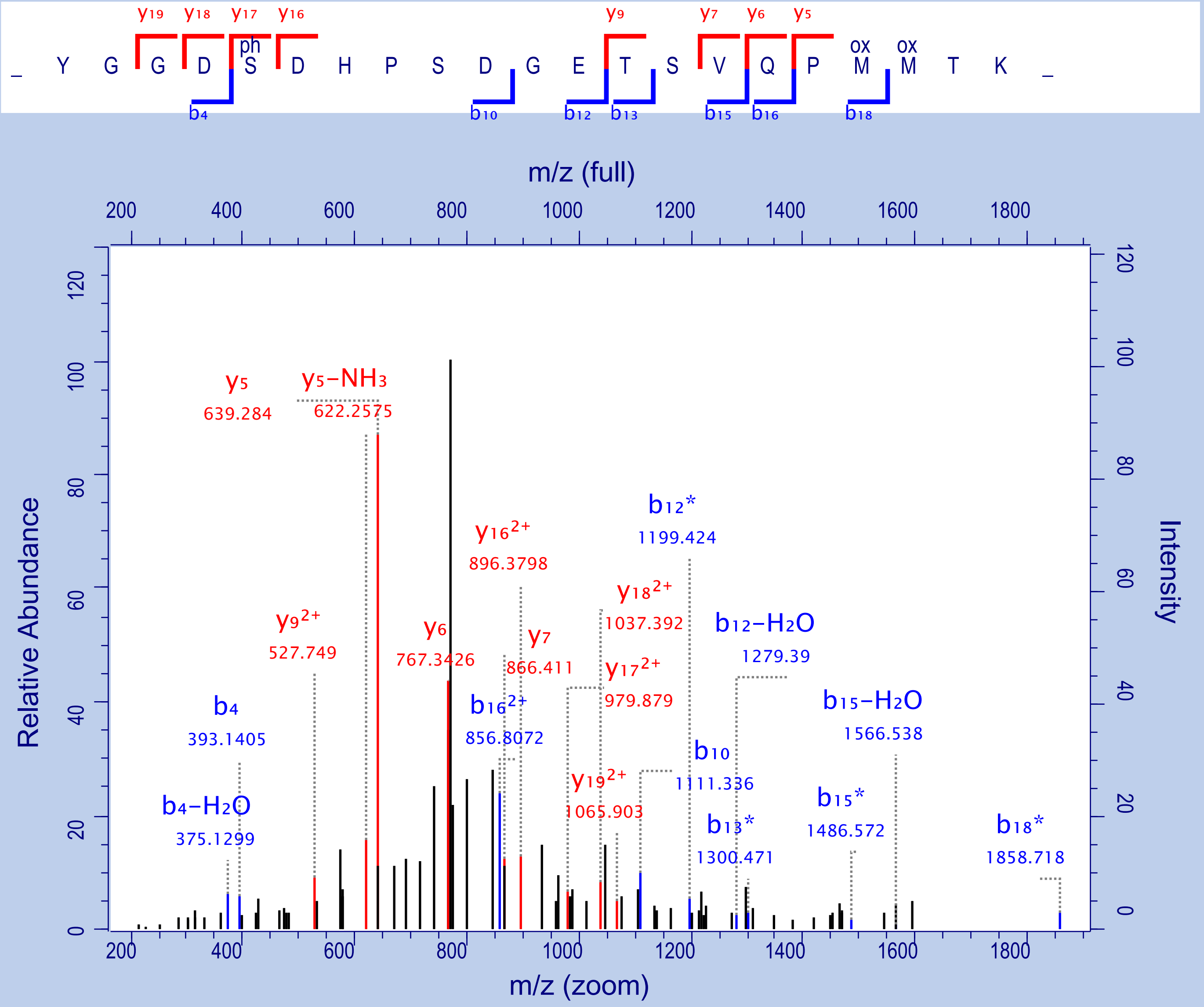

pSer271 and pSer275

Protein: Q8WYQ5-1; Q8WYQ5; Q53GY5; Q8WYQ5-3; Q8WYQ5; Q8WYQ5-2; Q8WYQ5
Score: 94.93
Source: OT11-1424
Scannumber: 2125
Method: ITMS; CID; 2

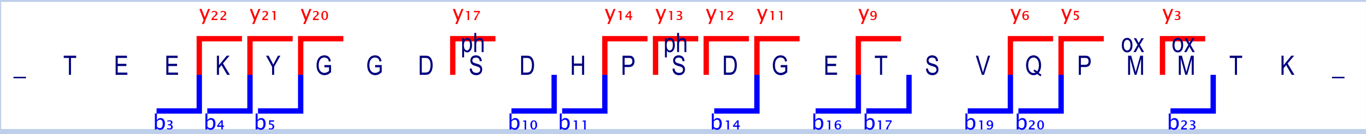

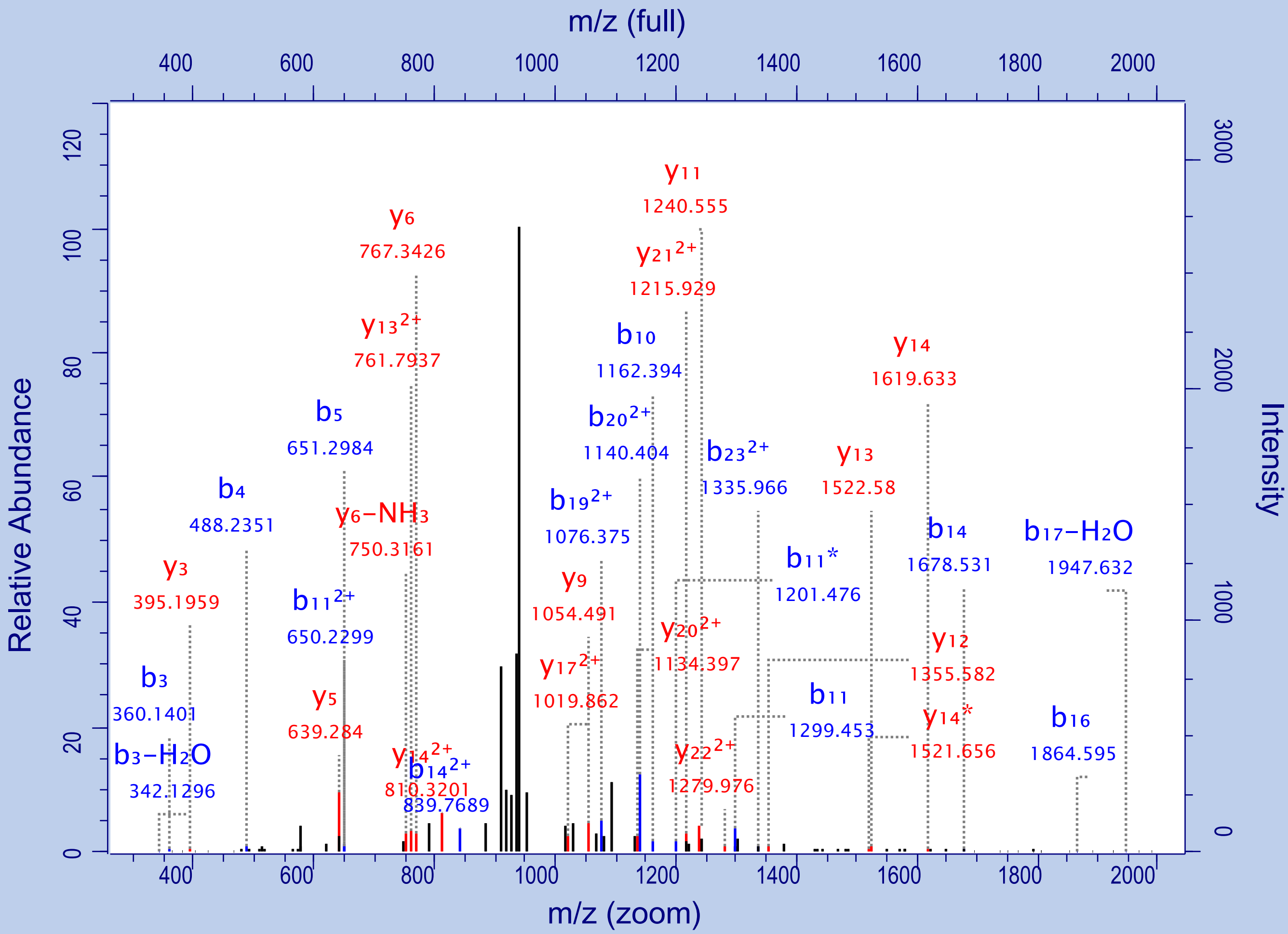

pSer275

Protein: Q8WYQ5-1; Q8WYQ5; Q53GY5; Q8WYQ5-3; Q8WYQ5; Q8WYQ5-2; Q8WYQ5
Score: 79.11
Source: OT11-1419
Scannumber: 2082
Method: ITMS; CID; 2

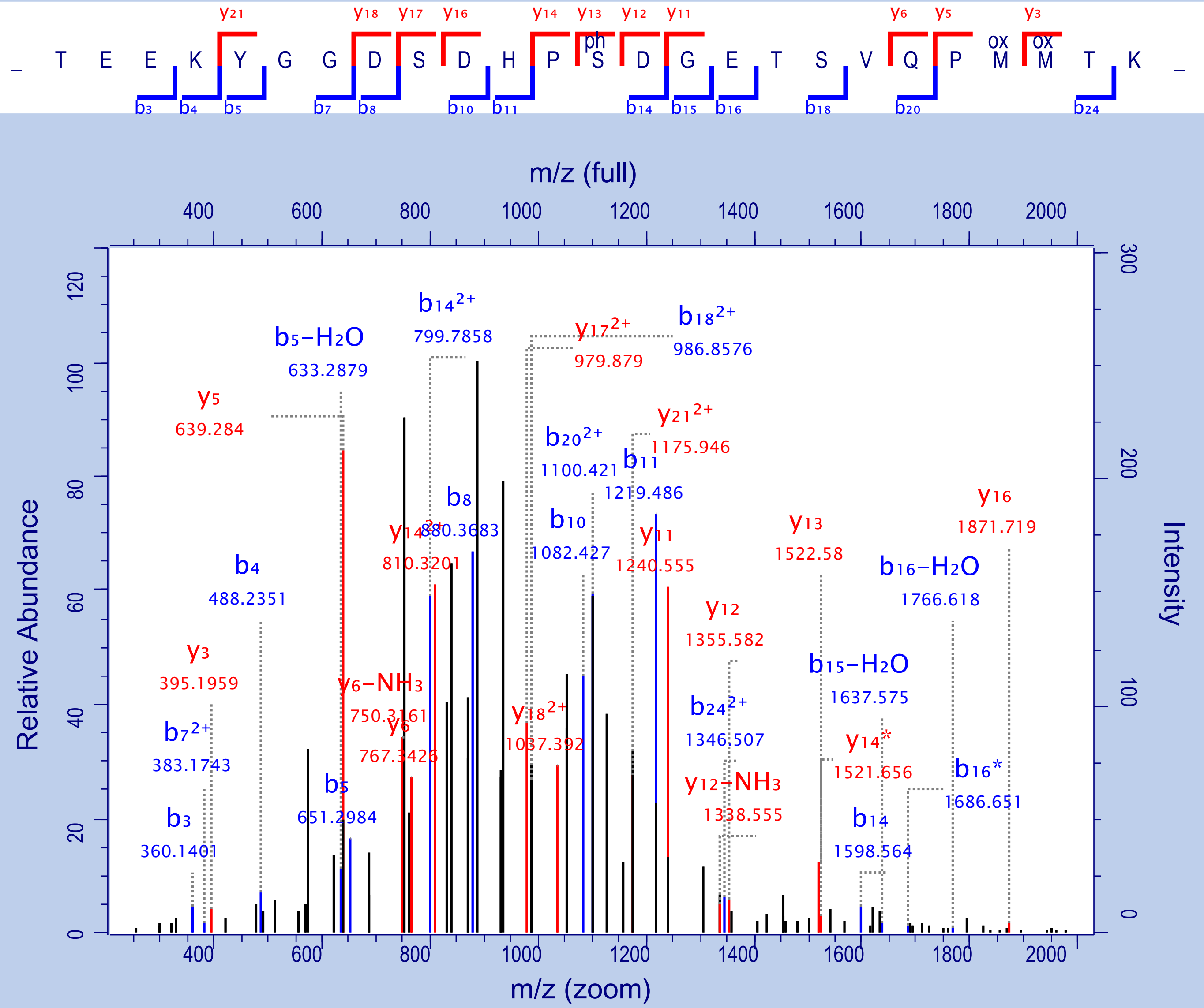

pSer271 and pThr279

Protein: Q8WYQ5-1; Q8WYQ5; Q53GY5; Q8WYQ5-3; Q8WYQ5; Q8WYQ5-2; Q8WYQ5
Score: 96.68
Source: OT11-1417
Scannumber: 2740
Method: ITMS; CID; 2

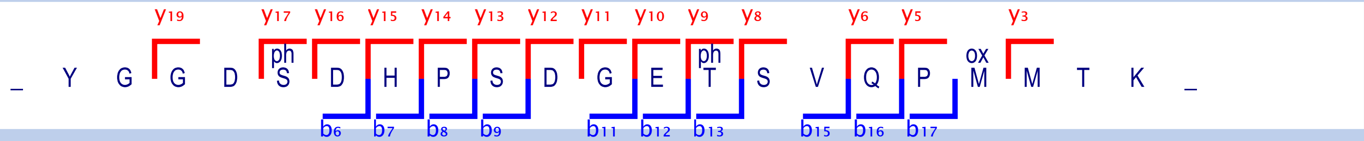

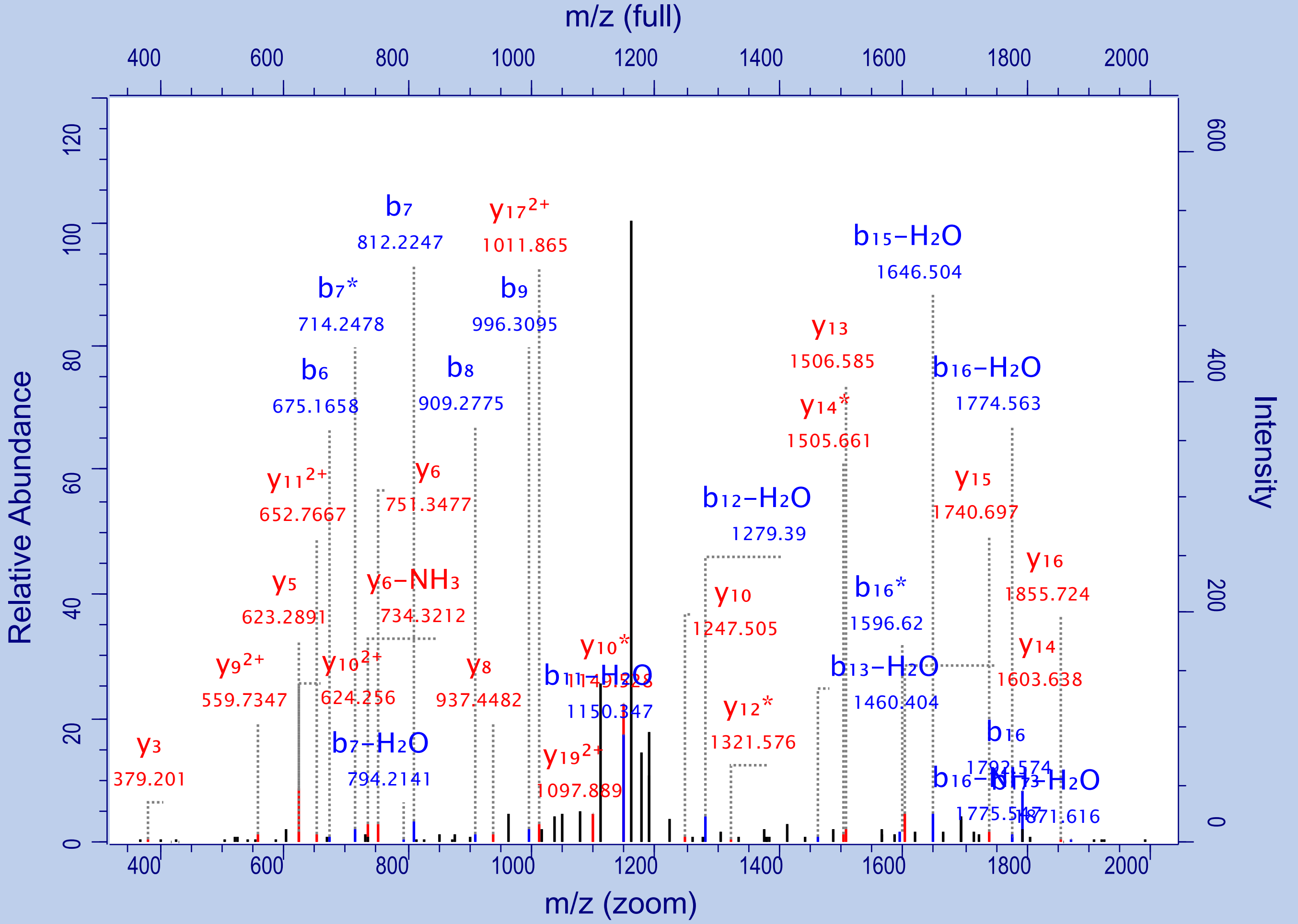

pSer271 and pSer280

Protein: Q8WYQ5-1; Q8WYQ5; Q53GY5; Q8WYQ5-3; Q8WYQ5; Q8WYQ5-2; Q8WYQ5
Score: 39.68
Source: OT11-1419
Scannumber: 2031
Method: ITMS; CID; 2

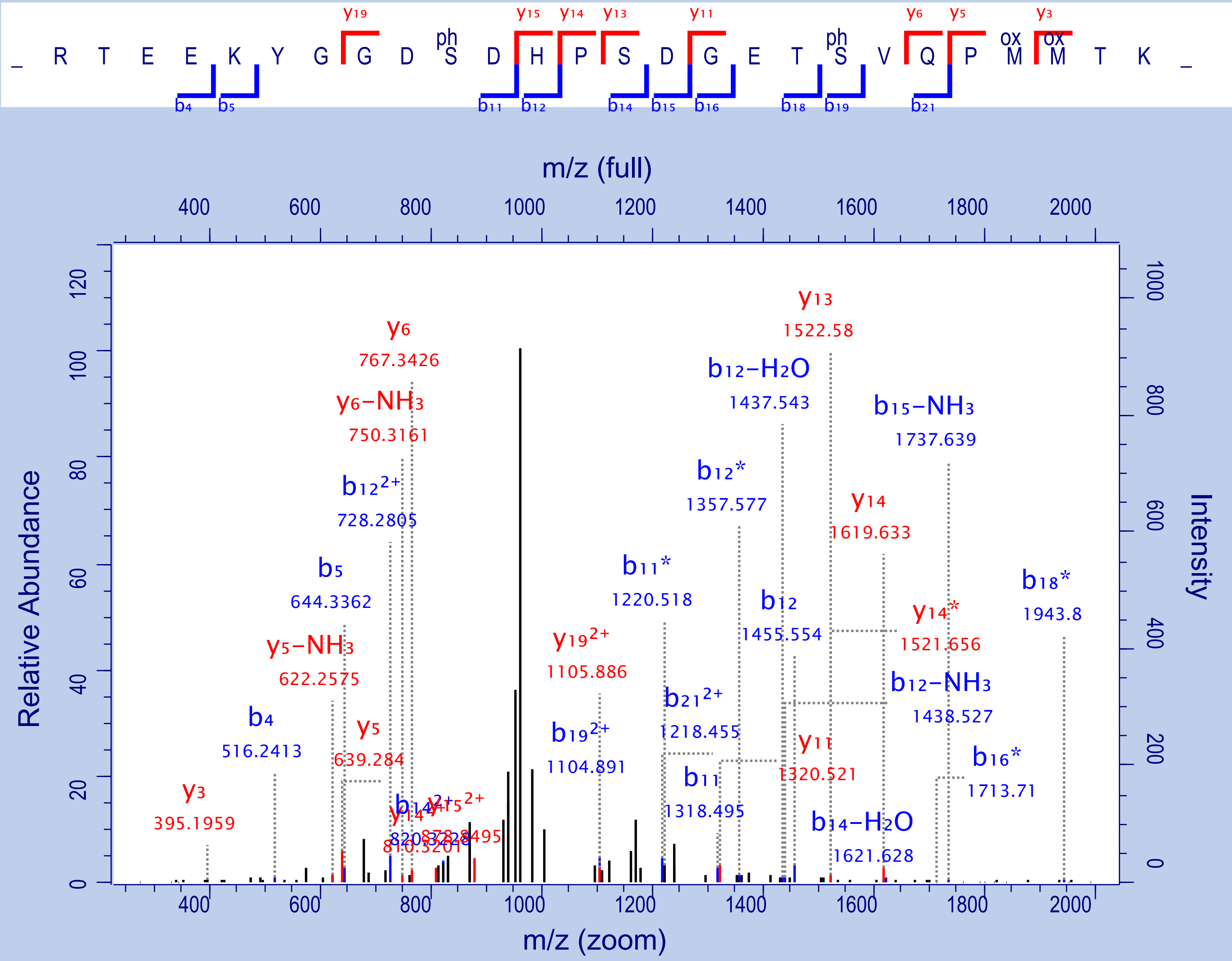

pThr371 and pSer383

Protein: Q8WYQ5-1; Q8WYQ5; Q53GY5; Q8WYQ5-3; Q8WYQ5
Score: 25.47
Source: OT11-1417
Scannumber: 3567
Method: ITMS; CID; 2

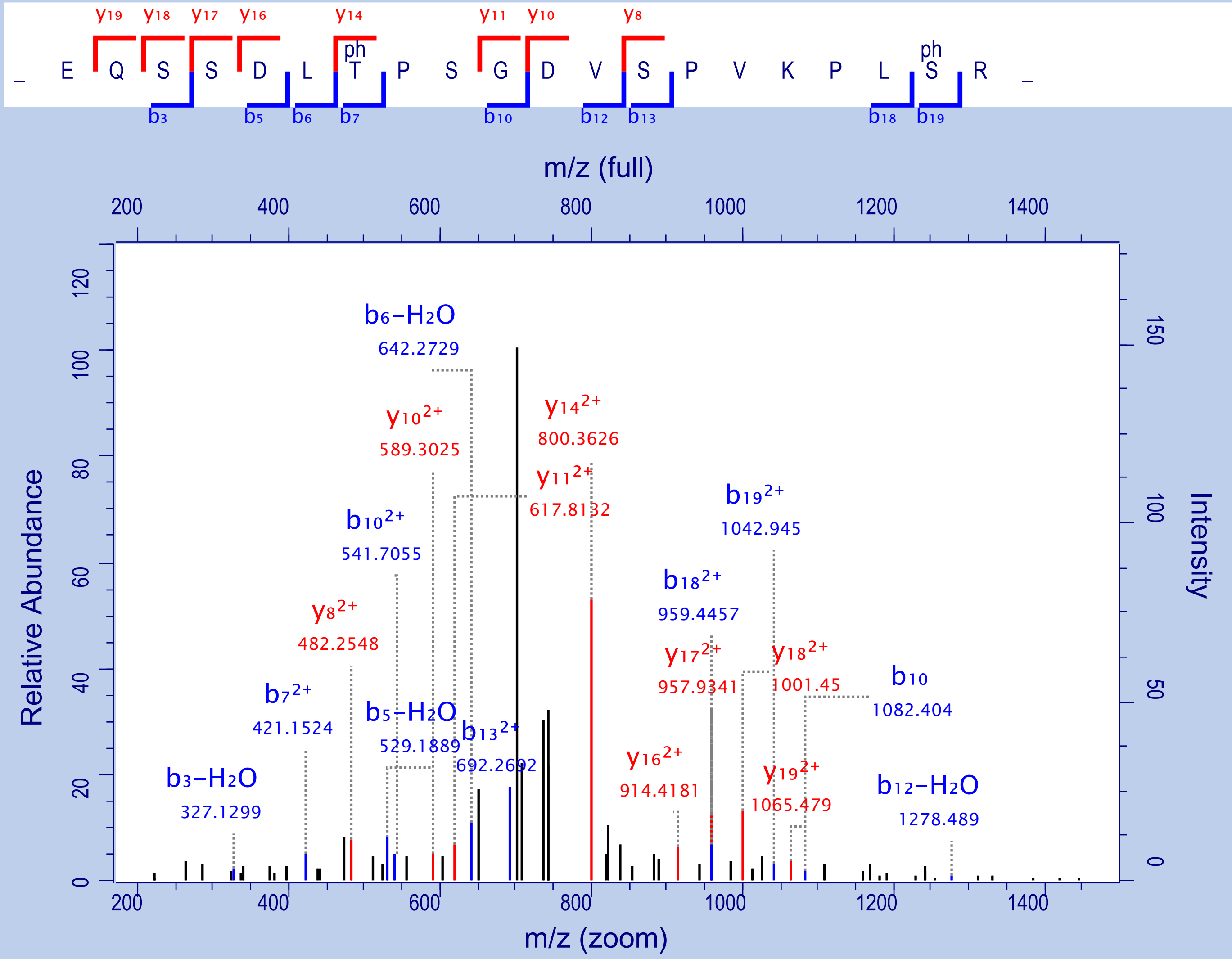

pSer373

Protein: Q8WYQ5-1; Q8WYQ5; Q53GY5; Q8WYQ5-3; Q8WYQ5
Score: 7.79
Source: OT11-1417
Scannumber: 3532
Method: ITMS; CID; 2

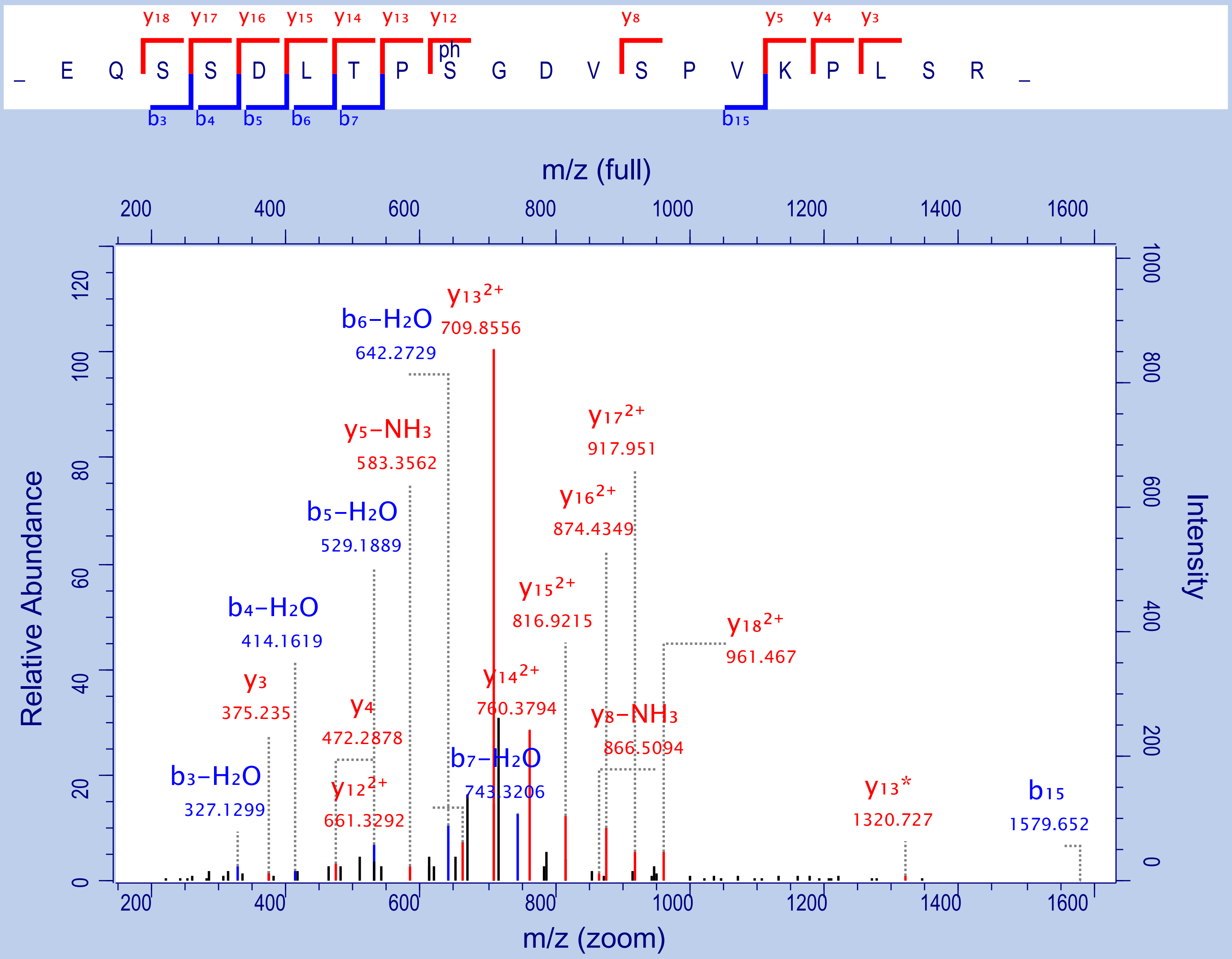

pSer373 and pSer377

Protein: Q8WYQ5-1; Q8WYQ5; Q53GY5; Q8WYQ5-3; Q8WYQ5
Score: 134.4
Source: OT11-1424
Scannumber: 3517
Method: ITMS; CID; 2

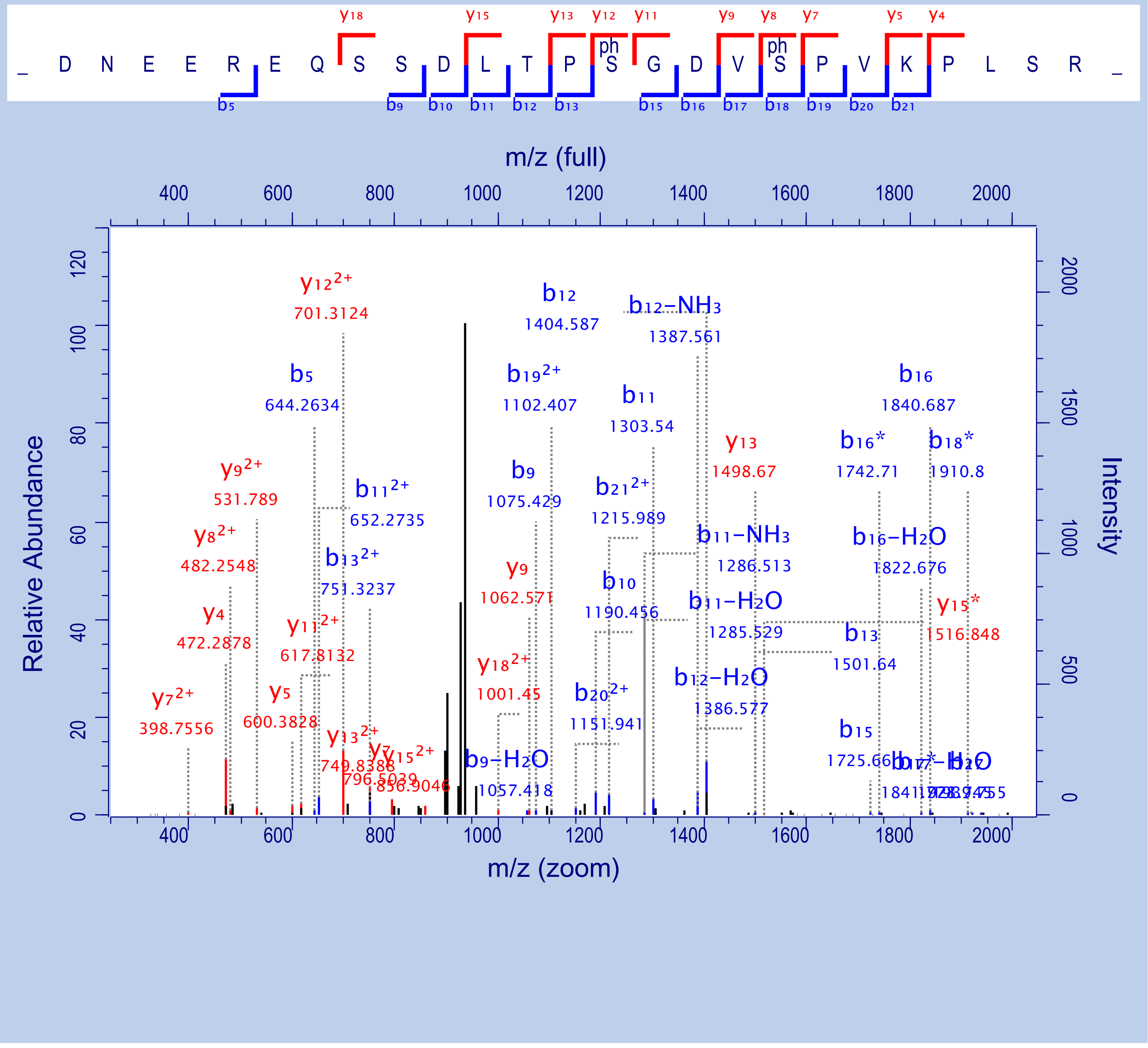

pSer377

Protein: Q8WYQ5-1; Q8WYQ5; Q53GY5; Q8WYQ5-3; Q8WYQ5
Score: 87.72
Source: OT11-1423
Scannumber: 3620
Method: ITMS; CID; 2

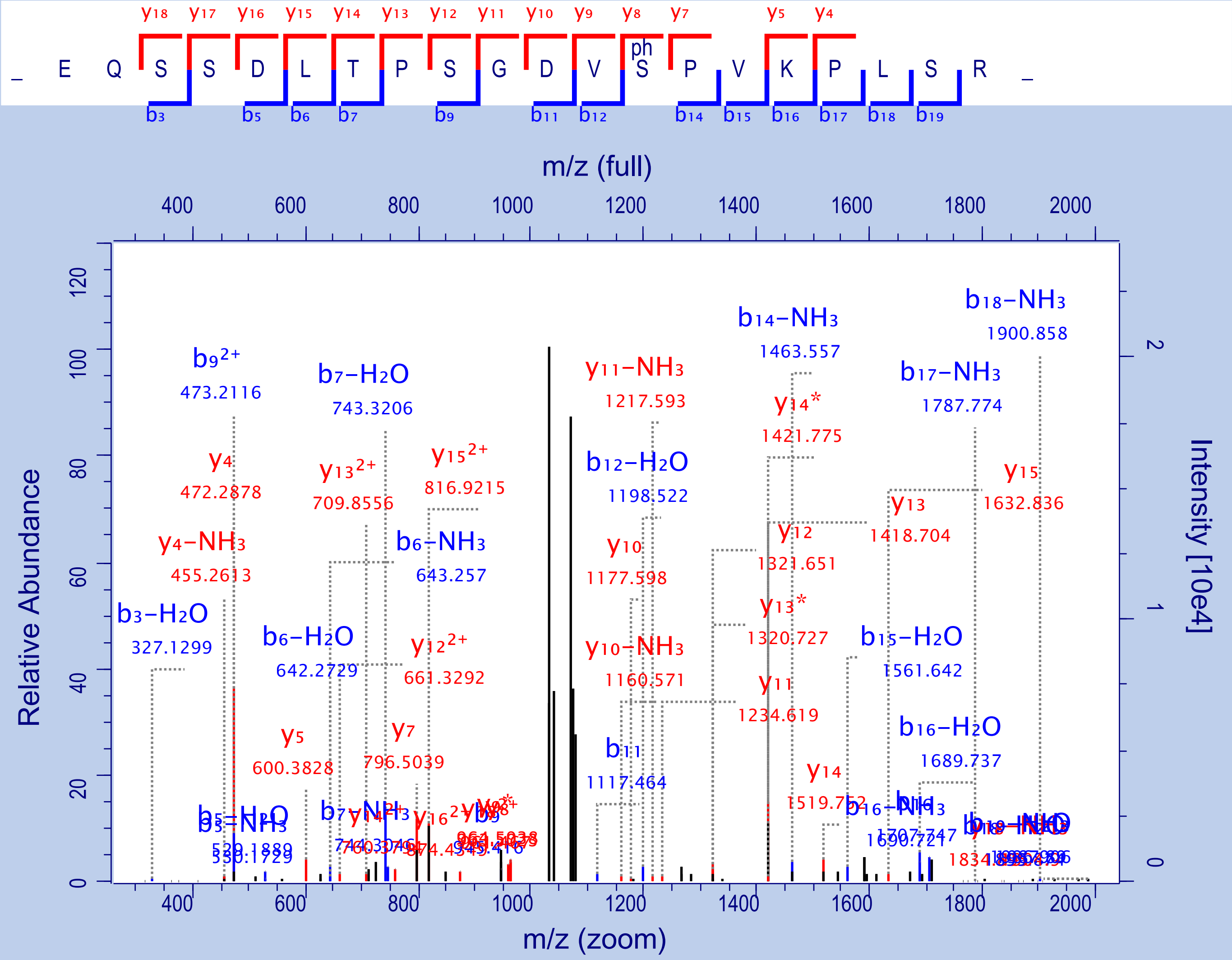

pSer385

Protein:	Q8WYQ5-1; Q8WYQ5; Q53GY5; Q8WYQ5-3; Q8WYQ5
Score:	74.8
Source:	OT10-0371_TiO2El
Scannumber:	4105
Method:	ITMS; CID; 2

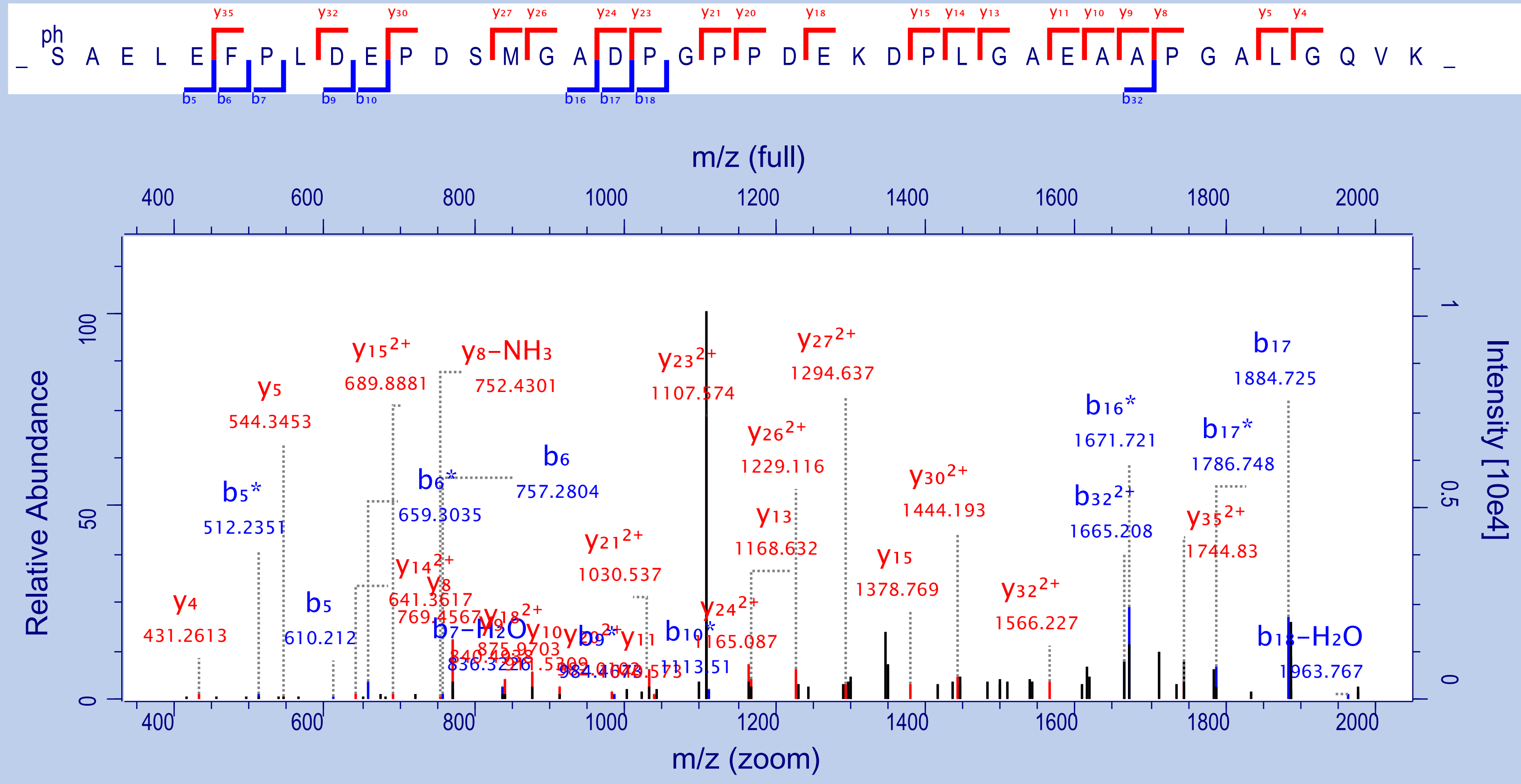

pSer397

Protein:	Q8WYQ5-1; Q8WYQ5; Q53GY5; Q8WYQ5-3; Q8WYQ5
Score:	63.31
Source:	OT11-1423
Scannumber:	6325
Method:	ITMS; CID; 2

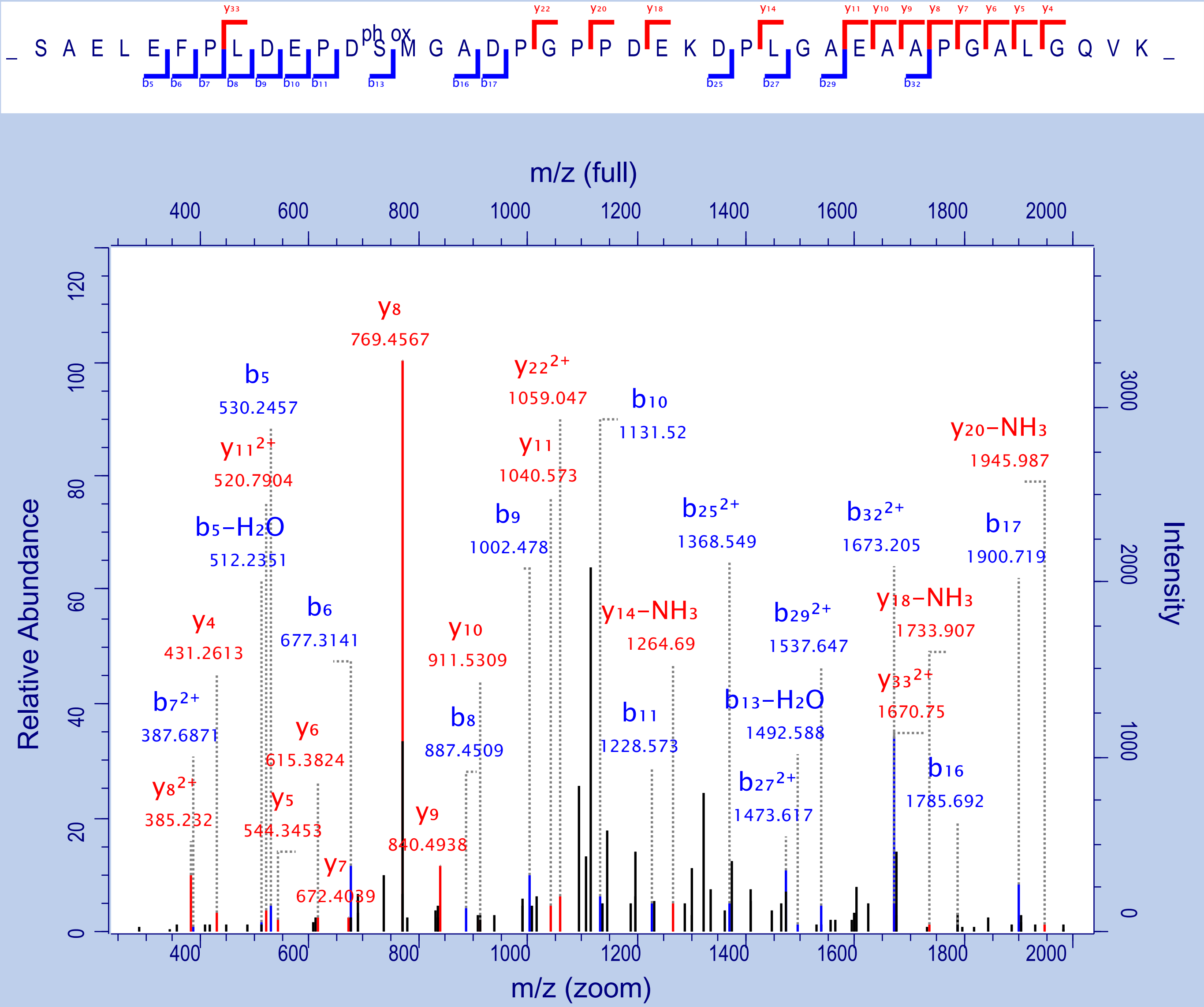

pSer434

Protein: Q8WYQ5-1; Q8WYQ5; Q53GY5; Q8WYQ5-3; Q8WYQ5
Score: 22.03
Source: OT10-0371_TiO2El
Scannumber: 3401
Method: ITMS; CID; 2

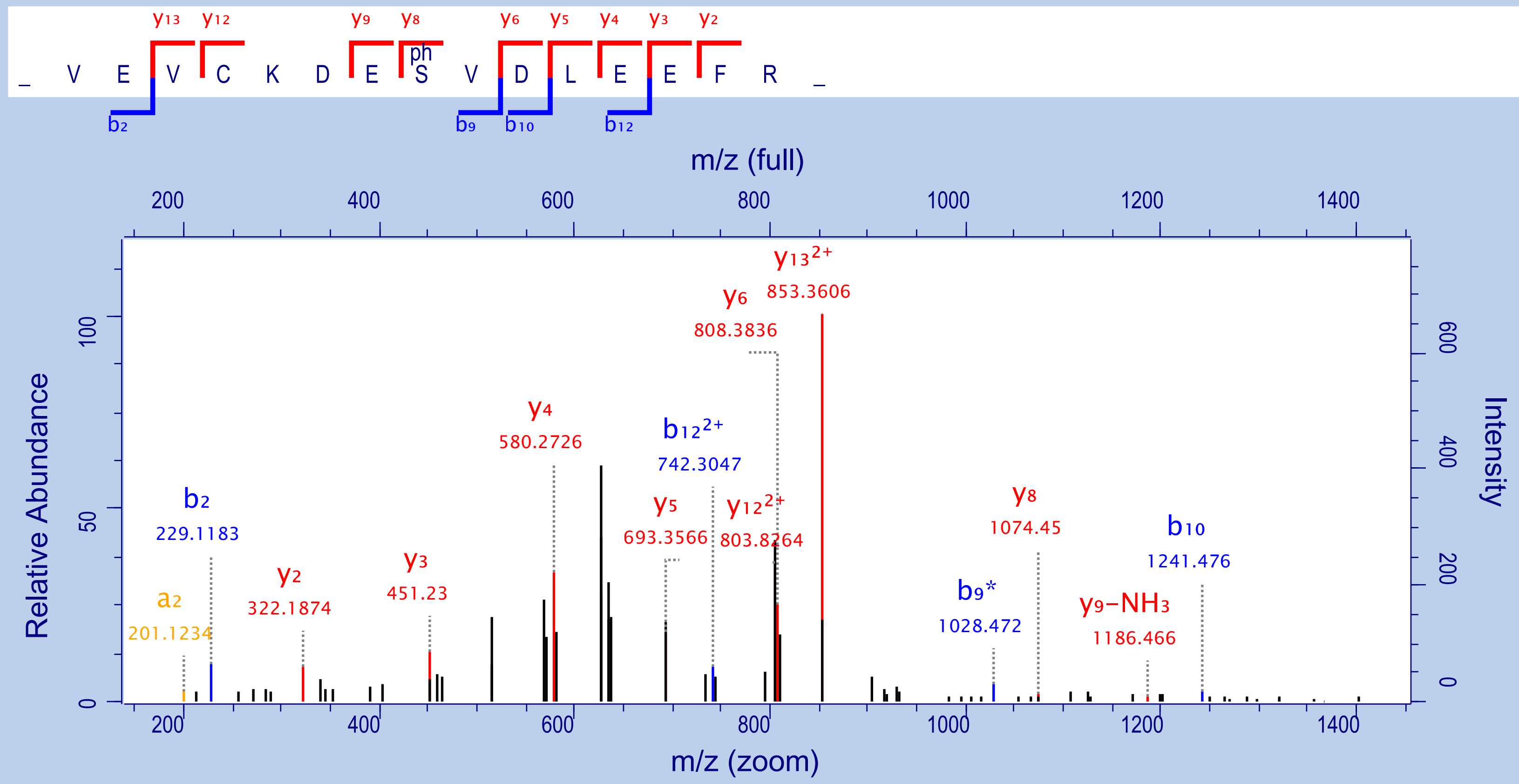

pSer454

Protein: Q8WYQ5-1; Q8WYQ5; Q53GY5; Q8WYQ5-3; Q8WYQ5
Score: 9.2
Source: OT11-1424
Scannumber: 3659
Method: ITMS; CID; 2

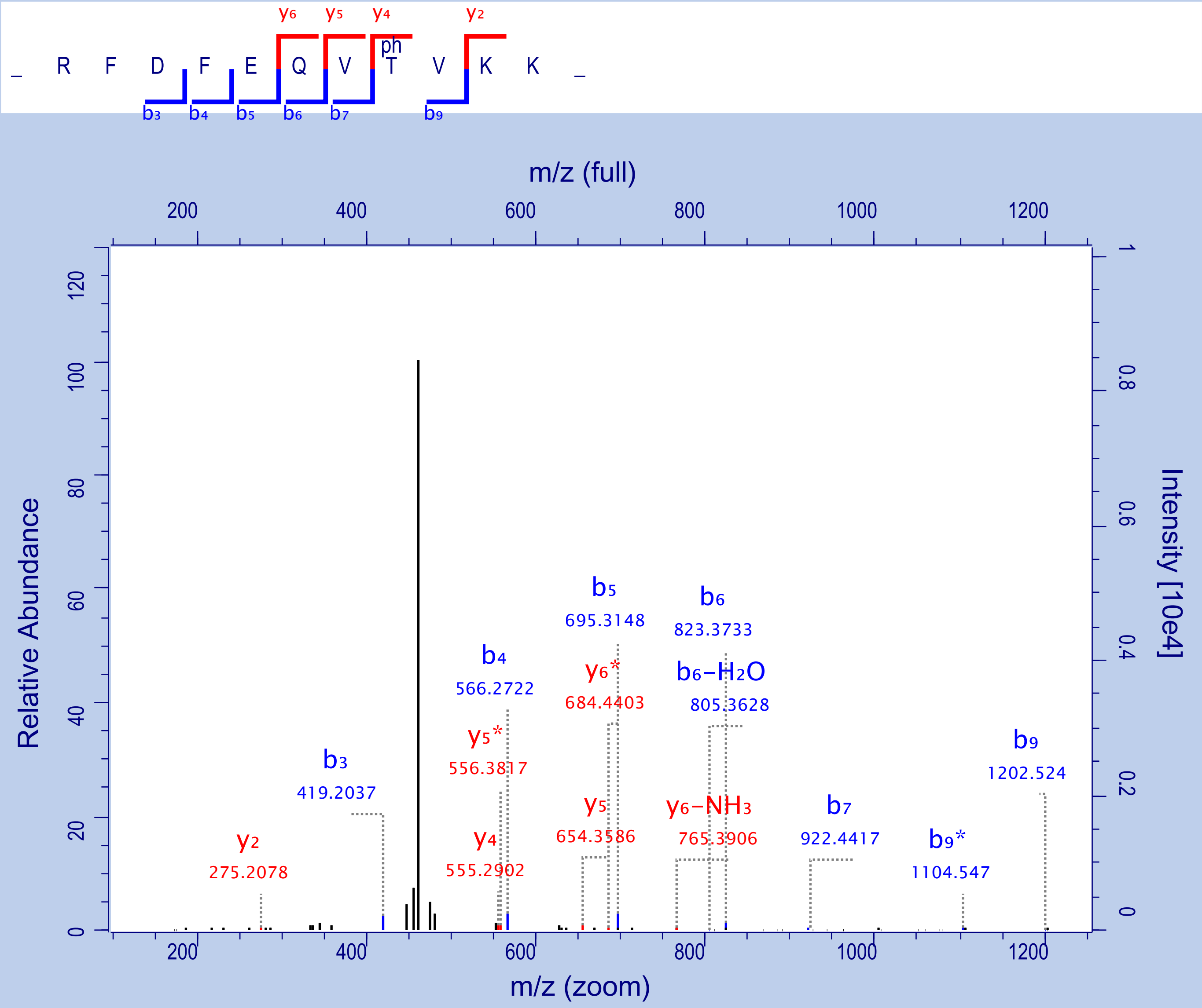

pSer493

Protein:	Q8WYQ5-1; Q8WYQ5; Q53GY5; Q8WYQ5-3; Q8WYQ5
Score:	123.14
Source:	OT11-1423
Scannumber:	3838
Method:	ITMS; CID; 2

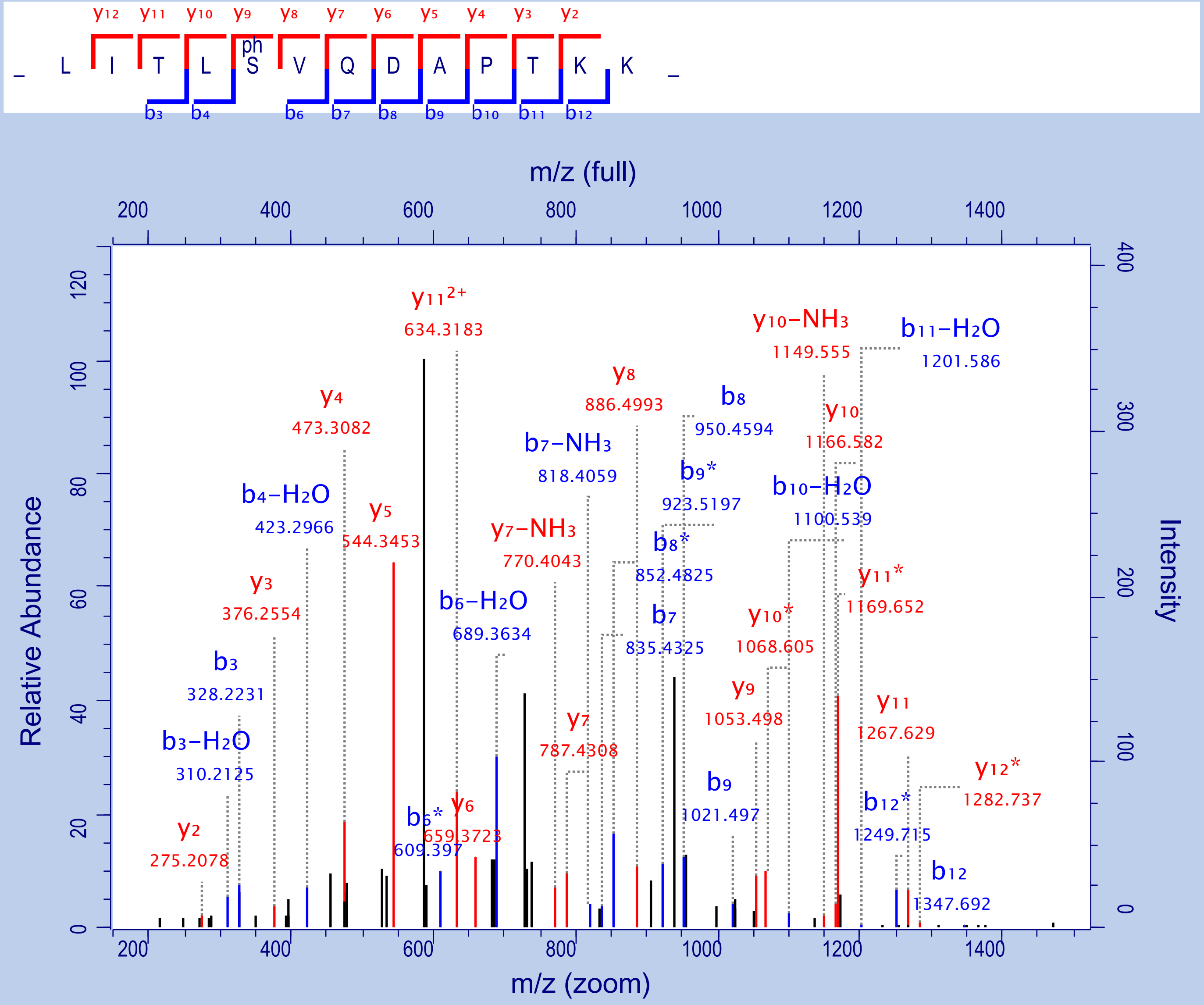

pSer714

Protein: Q8WYQ5-1; Q8WYQ5; Q53GY5; Q8WYQ5-3; Q8WYQ5
Score: 58.92
Source: OT11-1423
Scannumber: 3808
Method: ITMS; CID; 2

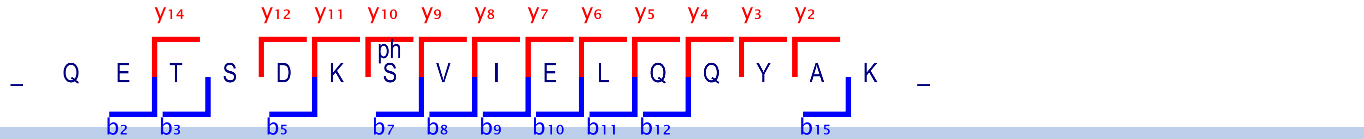

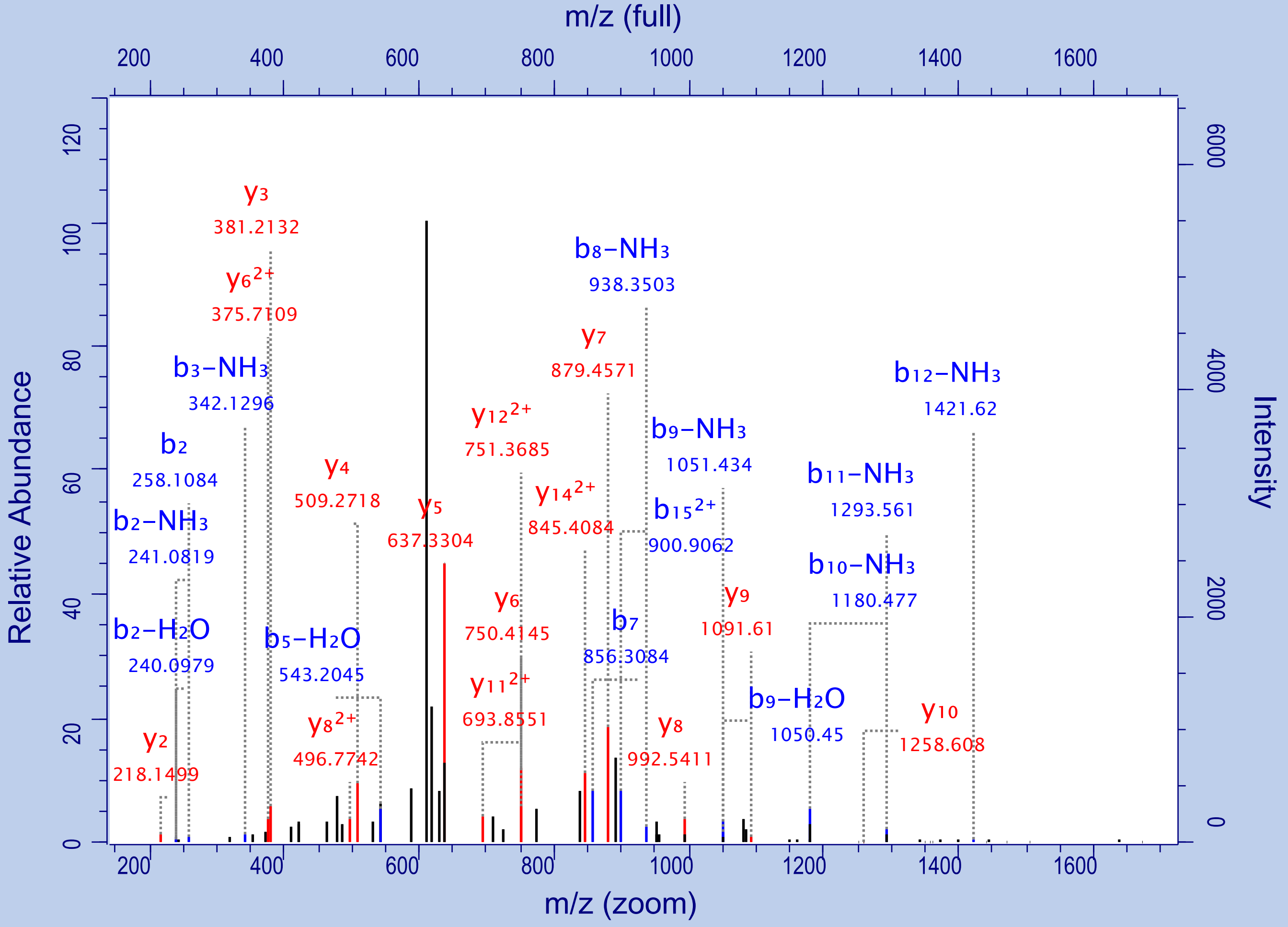

Supplement: 3 [file NIHMS542053-supplement-3.pdf]
