# Supplementary material for: Phosphorylation of DGCR8 Increases Its Intracellular Stability and Induces a Progrowth miRNA Profile
Source: Cell Rep. Author manuscript; Available in PMC 2014 Nov 27. (PMC3892995; doi:10.1016/j.celrep.2013.10.017)
Supplement: 4 [file NIHMS542053-supplement-4.pdf]

Cell PRESS
Open ACCESS
Cell Reports
Article

Phosphorylation of DGCR8 Increases Its Intracellular Stability and Induces a Progrowth miRNA Profile

Kristina M. Herbert,[1] Genaro Pimienta,[1] Suzanne J. DeGregorio,[1] Andrei Alexandrov,[1] and Joan A. Steitz[1,*]
[1]Department of Molecular Biophysics and Biochemistry, Howard Hughes Medical Institute, Yale University School of Medicine, New Haven, CT 06536, USA
*Correspondence: joan.steitz@yale.edu
http://dx.doi.org/10.1016/j.celrep.2013.10.017
This is an open-access article distributed under the terms of the Creative Commons Attribution-NonCommercial-No Derivative Works License, which permits non-commercial use, distribution, and reproduction in any medium, provided the original author and source are credited.

SUMMARY

During miRNA biogenesis, the microprocessor complex (MC), which is composed minimally of Drosha, an RNase III enzyme, and DGCR8, a double-stranded RNA-binding protein, cleaves the primary miRNA (pri-miRNA) in order to release the pre-miRNA stem-loop structure. Using phosphoproteomics, we mapped 23 phosphorylation sites on full-length human DGCR8 expressed in insect or mammalian cells. DGCR8 can be phosphorylated by mitogenic ERK/MAPK, indicating that DGCR8 phosphorylation may respond to and integrate extracellular cues. The expression of phosphomimetic DGCR8 or inhibition of phosphatases increased the cellular levels of DGCR8 and Drosha proteins. Increased levels of phosphomimetic DGCR8 were not due to higher mRNA levels, altered DGCR8 localization, or DGCR8's ability to self-associate, but rather to an increase in protein stability. MCs incorporating phosphomutant or phosphomimetic DGCR8 were not altered in specific processing activity. However, HeLa cells expressing phosphomimetic DGCR8 exhibited a progrowth miRNA expression profile and increased proliferation and scratch closure rates relative to cells expressing phosphomutant DGCR8.

INTRODUCTION

miRNAs are ~22 nt long and posttranscriptionally regulate their target mRNAs through degradation and translational repression (Guo et al., 2010). They are involved in a diverse array of biological processes ranging from cell growth, survival, and differentiation to disease states such as cancer. miRNA genes are typically transcribed by RNA polymerase II into long, capped, and polyadenylated primary transcripts (pri-miRNAs), which follow a two-step processing pathway to yield a mature miRNA. The nuclear microprocessor complex (MC), which is composed of the ribonuclease (RNase) III enzyme Drosha and its essential cofactor DGCR8, excises a ~70 nt stem-loop structure (the pre-miRNA) with a 5′ phosphate and a ~2 nt 3′ overhang (Denli et al., 2004; Gregory et al., 2004; Han et al., 2004; Landthaler et al., 2004). This step is critical for proper miRNA biogenesis because the Drosha cleavage site defines the sequence of the mature miRNA by generating one end of the ~22 nt mature miRNA. The resulting pre-miRNA is then transported by the Exportin-5/Ran-GTP complex to the cytoplasm, where it is further processed by the RNase III enzyme Dicer. Dicer, together with a double-stranded RNA binding domain (dsRBD)-containing protein, TRBP2, cleaves the upper hairpin stem, generating ~2 nt 3′ overhangs on the ~22 nt dsRNA product (Chendrimada et al., 2005; Haase et al., 2005). One strand is then incorporated into an RNA-induced silencing complex (RISC), whose main component is an Argonaute family protein. This complex targets mRNAs via basepairing between the miRNA and mRNA, resulting in the regulation of protein expression.

Several proteins involved in miRNA processing are regulated by posttranslational modifications (PTMs). TRBP2 stability is increased upon phosphorylation by extracellular signal-regulated kinases (ERKs), leading to increased Dicer and progrowth miRNA levels (Paroo et al., 2009). Upon cell-cycle reentry, Exportin 5 expression is posttranscriptionally induced in a phosphoinositide 3-kinase (PI3K) pathway-dependent process (Iwasaki et al., 2013). Phosphorylation of Drosha by glycogen synthase kinase-3β (GSK3β) is required for proper Drosha localization to the nucleus (Tang et al., 2010, 2011), and acetylation of Drosha inhibits its degradation (Tang et al., 2013). The ability of DGCR8 to bind RNA has been reported to be modulated by acetylation of lysine residues within its dsRBDs (Wada et al., 2012). Although ten phosphorylation sites in DGCR8 have been mapped in high-throughput tandem mass spectrometry (MS/MS) studies of total mammalian cell lysates (Dephoure et al., 2008; Olsen et al., 2006), the roles of these phosphorylations remain elusive.

DGCR8 function is clearly important, as it is essential for viability in mice and DGCR8-knockout embryonic stem cells show a proliferation defect (Wang et al., 2007). DGCR8 deficiency in the brain has also been suggested to cause behavioral and neuronal defects associated with the 22q11.2 deletion syndrome

known as DiGeorge syndrome (Schofield et al., 2011; Stark et al., 2008). As an essential component of the MC, DGCR8 (1) localizes to the nucleus, (2) associates with Drosha and RNA, and (3) allows Drosha's RNase III domains to access the RNA substrate. The stoichiometry of DGCR8 and Drosha within the MC remains unclear (Gregory et al., 2004; Han et al., 2004); however, purified DGCR8 has been shown to form a dimer (Barr et al., 2011; Faller et al., 2007; Senturia et al., 2012). It is therefore possible that DGCR8's subcellular localization and/or ability to associate with cofactors (RNA, Drosha, or itself) could be affected by phosphorylation. Likewise, the altered phosphorylation status of DGCR8 in conditions of uncontrolled cell signaling, as in cancer cells, could contribute to the disease phenotype.

In this study, we confirm that human DGCR8 is phosphorylated in metazoan cells. Using peptide fractionation and phosphopeptide enrichment strategies, we mapped 23 phosphosites on DGCR8, the 10 previously identified sites (Dephoure et al., 2008; Olsen et al., 2006), plus an additional 13. At least some of these sites are targeted by ERK, indicating an important regulatory function. By mutating these amino acids to either prevent or mimic phosphorylation, we found that multisite phosphorylation stabilized the DGCR8 protein. Expression of the mimetic DGCR8 construct showed increased protein levels relative to a wild-type (WT) DGCR8 construct and led to an altered progrowth miRNA expression profile, and enhanced cell proliferation. These data implicate DGCR8 as a critical link between extracellular proliferative cues and reprogramming of the cellular miRNA profile.

RESULTS

DGCR8 Is Multiply Phosphorylated

To verify that DGCR8 is phosphorylated in metazoan cells, we transiently expressed human N-terminally FLAG-hemagglutinin (HA)-tagged DGCR8 (FH-DGCR8) and Myc-Drosha in either human embryonic kidney (HEK) 293T or HeLa cells metabolically labeled with radioactive orthophosphate. DGCR8 immunoprecipitated from both cell lines showed ^{32}P incorporation (Figures 1A and 1B). To create a comprehensive phosphorylation profile, we expressed tagged human DGCR8 and immunopurified it from baculovirus-infected Hi-5 insect cells or transiently transfected HEK293 cells. Then, we coupled peptide fractionation protocols and phosphopeptide enrichment strategies with high-resolution MS/MS and MaxQuant software (Cox et al., 2011) for data analysis (Figure 1C). We obtained 73% total amino acid sequence coverage of DGCR8 from the baculovirus-infected insect cell culture (Figure S1), which allowed us to confirm nine of the ten phosphosites reported from high-throughput studies (Dephoure et al., 2008; Olsen et al., 2006) and map ten additional phosphosites (Table 1). In two independent experiments analyzing phosphosites on DGCR8 expressed in HEK293 cells, we obtained 53% and 60% sequence coverage, respectively (Figure S1). All ten known sites and four of the ten newly identified sites were confirmed, and three additional sites were mapped (Table 1). All of the identified sites exhibited high MaxQuant scores (>60) and low posterior error probability scores (<0.1) in at least one experiment, and most (19 of 23) were found in multiple peptides (Table 1). Sites that had scores lower than 60 or had not previously been identified in high-throughput studies were not considered further (Table S1). Representative spectra of phosphopeptides for each site are shown in Figure 1D and Data S1. Several examples of peptides phosphorylated at multiple sites were observed (Figure 1D lower spectra; Data S1), suggesting that multisite phosphorylation might be important for DGCR8 function.

Overall, we detected a total of 23 phosphorylation sites in DGCR8 (Figure 1E) with high statistical confidence. Most of these phospho-acceptor sites are conserved over a number of species (data not shown). All 23 sites occur in the N terminus of DGCR8, outside regions for which three-dimensional structures have been determined (Senturia et al., 2012; Sohn et al., 2007; Wostenberg et al., 2010). Consistent with global analyses of the structural context of phosphorylation sites (Holt et al., 2009), a secondary structure prediction of DGCR8 suggests that 21 of the 23 sites reside in loops that should be accessible to kinases and may represent regions of protein-protein interactions (data not shown).

To ensure that we mapped all relevant phosphosites in DGCR8 under our growth conditions, we mutated each of the 23 phosphosites in the FH-DGCR8 construct to either prevent or mimic phosphorylation (hereafter referred to as Mut23 and Mim23, respectively; see Table S2 for details). Immunoprecipitation of Mut23 from cells metabolically labeled with ^{32}P-orthophosphate showed no ^{32}P signal, whereas Mim23 showed less signal than the WT, despite higher total protein levels (Figure 1F). The remaining ^{32}P signal for Mim23 may be due to phosphorylation at phosphosites identified with lower statistical confidence (Table S1). The higher DGCR8 protein levels resulting from expression of the Mim23 construct suggested that phosphorylation might stabilize the exogenous DGCR8 protein.

DGCR8 Is Phosphorylated by Mitogenic MAPKs

Methods for predicting kinase-substrate pairs suggested that many cellular kinases could be involved in phosphorylating DGCR8 (Table S3). However, from a panel of phospho-(Ser/Thr) kinase substrate antibodies (MAPK/CDK, AKT, PKA, ATM/ATR, and PKC), DGCR8 immunopurified from insect cells was recognized by the anti-MAPK/CDK substrate antibody (Figure 2A). Since DGCR8 possesses MAPK docking motifs that match both of the recently structurally defined motifs that are specific for JNK and ERK/p38 kinases (Garai et al., 2012; Figure S2A), we probed immunoblots of anti-FLAG-immunoprecipitated MCs from HEK 293T cell extracts for the presence of these kinases (Figure 2B). JNK1 and JNK2 and ERK1 and ERK2, but not p38, were specifically coimmunoprecipitated, but not from the negative control extract where DGCR8 with an alternate tag (SNAP) was expressed. Protein phosphatase 2A subunit A was also coimmunoprecipitated with MCs (Figure 2B), pointing to an equilibrium between phosphorylation and dephosphorylation that might be regulated by cellular conditions.

To confirm that JNK and ERK can phosphorylate DGCR8, we performed in vitro kinase assays with bacterially expressed DGCR8 and immunopurified kinases. A constitutively active form of JNK (FLAG-MKK7B2-JNK1a1 WT: FLAG-JNK1a1 fused to its upstream kinase MKK7; Zheng et al., 1999) or the significantly less active WT JNK1a1, expressed and immunopurified from HEK 293T cells (Figure S2B, left) was specifically able to

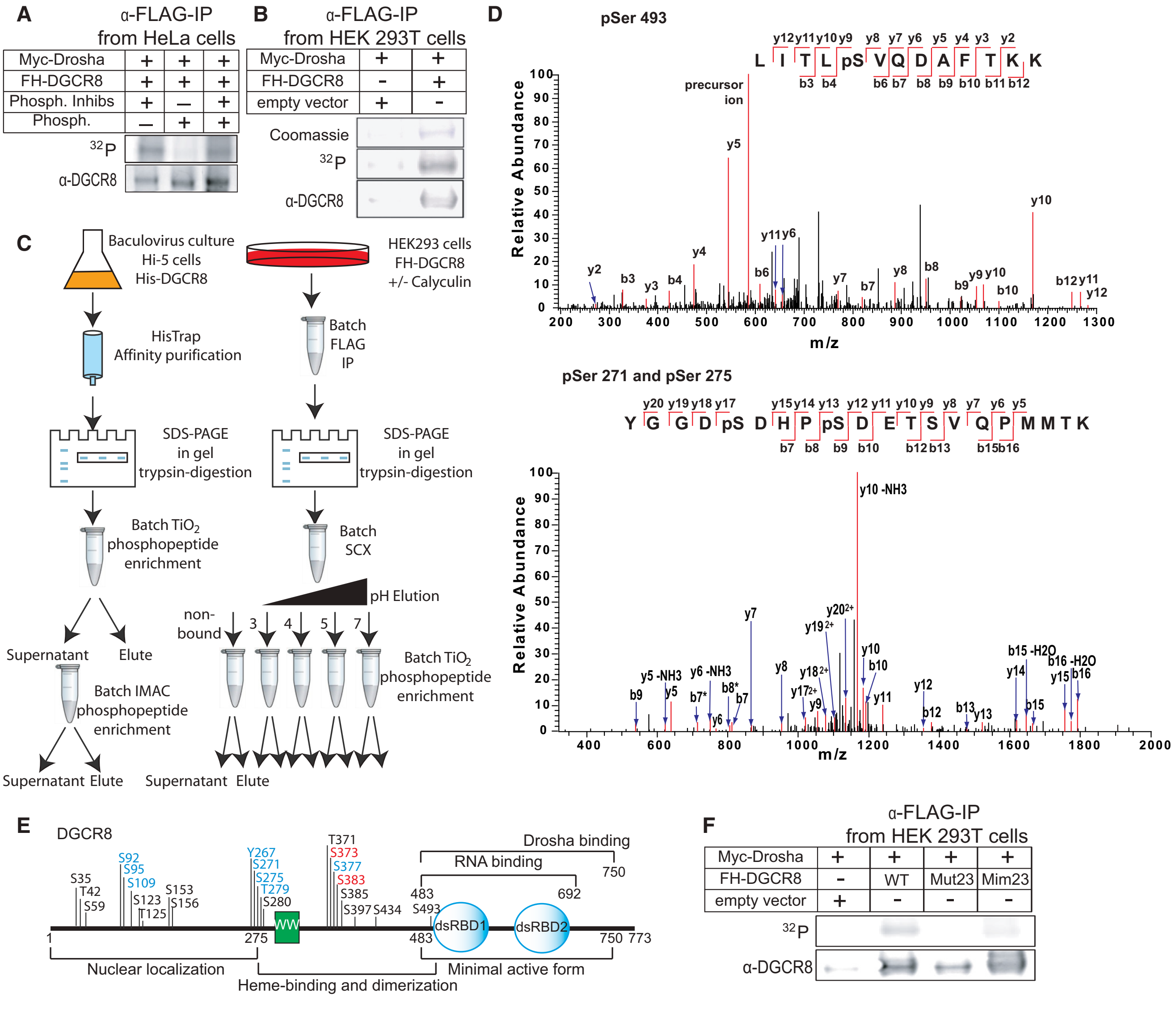

Figure 1. DGCR8 Is Multiply Phosphorylated

(A and B) DGCR8 expressed in mammalian cell lines shows ^{32}P incorporation due to phosphorylation. HeLa (A) and HEK 293T (B) cells were transiently transfected with vectors expressing FH-DGCR8, Myc-Drosha, and GFP (as a transfection control). Cells were metabolically labeled with $^{32}PO_4$, and anti-FLAG immunoprecipitation was used to isolate MCs. Immunoblots, ^{32}P, and Coomassie-stained gel images are shown. Lanes that were not run next to each other were moved together. In (A), immunopurified MCs were subjected to phosphatase treatment either alone or in the presence of phosphatase inhibitors.

(C) Purification and enrichment scheme for isolating DGCR8 phosphopeptides.

(D) Representative fragmentation spectra of two identified phosphopeptides. Each spectrum shows relative intensity measurements of mass-to-charge ratios (m/z) after assigning the most abundant ion 100%. The "b" and "y" fragmentation ions are indicated in red along the peptide sequence assigned to the spectrum. Phosphorylated residues are indicated with a "p." Spectra are labeled as follows: precursor ion (unfragmented peptide), −H20 (water loss), −NH3 (ammonium loss), * (oxidation), and 2+ or 3+ (charge). Red lines indicate signals corresponding to assigned peptide fragments, and black lines are unassigned. Each fragmentation spectrum explains the assigned sequence.

(E) Schematic diagram of the domain structure of DGCR8 with the 23 mapped phosphosites indicated. Previously identified sites are shown in red (Olsen et al., 2006) and blue (Dephoure et al., 2008). Regions that are important for various functions of DGCR8 are indicated (Faller et al., 2007; Yeom et al., 2006).

(F) HEK 293T cells were transiently transfected with vectors expressing GFP (as a transfection and RNA loading control), Myc-Drosha, and either an empty vector or WT-FH-DGCR8, Mut23-FH-DGCR8, or Mim23-FH-DGCR8. Cells were metabolically labeled with $^{32}PO_4$ and MCs isolated via anti-FLAG immunoprecipitation. Immunoblots (bottom) and ^{32}P (top) images are shown. See also Figure S1, Data S1, and Table S2.

phosphorylate DGCR8 in vitro (Figure S2B, right). Activated ERK was obtained by coexpressing and immunoprecipitating HA-ERK with a constitutively active (R4F) version of its upstream kinase MKK1, whereas HA-ERK expressed with a kinase-dead (K97M) version of MKK1 or without any MKK1 yielded inactive ERK (Figure S2C). Only activated ERK was able to phosphorylate

Cell PRESS Open ACCESS

Table 1. Phosphosites Mapped on His-DGCR8 Isolated from HEK293 or Hi-5 Insect Cells

	HEK293 Cells Experiment 1			HEK293 Cells Experiment 2			Hi-5 Insect Cells		
	Coverage 52.9%		No. of Peptides Phosphorylated	Coverage 60.0%		No. of Peptides Phosphorylated	Coverage 73.5%		No. of Peptides Phosphorylated
aa Sites	PEP	Score	Total	PEP	Score	Total	PEP	Score	Total
S35	1.34×10^{-19}	113.1	2/2	NA	NA	0/0	8.59×10^{-14}	158.2	7/8
T42	NA	NA	0/2	NA	NA	0/0	8.59×10^{-14}	158.2	1/8
S59	NA	NA	0/2	NA	NA	0/0	9.01×10^{-2}	83.4	1/8
**S92**	NA	NA	0/6	3.32×10^{-2}	56.4	1/45	8.60×10^{-2}	72.2	1/9
**S95**	NA	NA	0/6	4.58×10^{-71}	101.4	3/45	8.60×10^{-2}	72.2	1/9
**S109**	1.92×10^{-2}	40.7	8/22	4.32×10^{-2}	40.8	7/26	3.88×10^{-1}	31.4	2/7
S123	NA	NA	0/0	1.21×10^{-3}	72.1	3/51	NA	NA	0/2
T125	NA	NA	0/0	1.21×10^{-3}	72.1	1/51	NA	NA	0/2
S153	NA	NA	0/0	NA	NA	0/1	6.91×10^{-5}	112.1	7/10
S156	NA	NA	0/0	NA	NA	0/1	6.91×10^{-5}	112.1	2/10
**Y267**	2.40×10^{-16}	117.9	2/28	NA	NA	0/10	3.14×10^{-15}	164.5	1/35
**S271**	2.40×10^{-16}	117.9	23/28	1.88×10^{-85}	247.2	8/10	3.14×10^{-15}	164.5	22/35
**S275**	1.32×10^{-5}	79.1	17/28	1.88×10^{-85}	247.2	7/10	1.03×10^{-22}	183.8	19/35
**T279**	2.40×10^{-16}	117.9	2/28	NA	NA	0/10	NA	NA	0/35
S280	2.40×10^{-16}	117.9	1/28	NA	NA	0/10	NA	NA	0/35
T371	3.89×10^{-33}	134.4	2/37	2.89×10^{-43}	204.0	1/30	1.89×10^{-5}	122.5	7/28
S373	3.89×10^{-33}	134.4	3/37	2.89×10^{-43}	204.0	3/30	1.89×10^{-5}	122.5	4/28
**S377**	7.67×10^{-54}	152.8	21/37	1.05×10^{-15}	147.0	16/30	2.70×10^{-15}	169.0	22/28
S383	3.89×10^{-33}	134.4	1/37	1.05×10^{-15}	147.0	1/30	2.70×10^{-15}	169.0	1/28
S385	NA	NA	0/19	NA	NA	0/30	9.12×10^{-4}	101.5	4/23
S397	1.75×10^{-1}	63.3	3/19	NA	NA	0/4	9.12×10^{-4}	101.5	1/23
S434	NA	NA	0/5	7.54×10^{-2}	37.075	1/72	7.27×10^{-3}	92.5	4/14
S493	5.16×10^{-21}	123.1	1/59	3.49×10^{-2}	51.5	2/59	1.51×10^{-3}	102.7	1/15

The amino acid number of each newly mapped phosphosite or previously identified phosphosite (italic [Dephoure et al., 2008] or bold [Olsen et al., 2006]) is shown with the values for the posterior error probability (PEP), and MaxQuant score. Also shown are the fractions of phosphorylated peptides (Total). See also Table S1.

bacterially expressed DGCR8, yielding ^{32}P-phosphorylated bands that increased in intensity with increasing kinase (Figure 2C, top) or substrate (Figure 2C, bottom) levels. To determine whether these kinases also phosphorylate DGCR8 in vivo, we serum starved a HeLa cell line that we developed to stably overexpress FLAG-DGCR8 (F-DGCR8) from a chromosomal locus (Flp-In cells; see the Supplemental Experimental Procedures) overnight, added either DMSO, the MKK1 inhibitor UO126, or the JNK inhibitor SP600125 prior to serum, and metabolically labeled the cells with ^{32}P-orthophosphate. When we immunoprecipitated DGCR8 and assessed the amount of ^{32}P incorporation, we found that U0126 reduced the levels of activated phospho-ERK induced by serum addition and also showed significantly less ^{32}P incorporation into DGCR8 (Figure 2D) relative to the DMSO control. These results indicate that DGCR8 is phosphorylated by ERKs in response to serum addition. The JNK inhibitor SP600125 increased the ^{32}P-DGCR8 levels (Figure 2D) relative to cells treated with the DMSO control, possibly due to the compensatory overactivation of ERK kinases that is often observed during the inhibition of other MAPKs (Ohashi et al., 2004; Paroo et al., 2009). However, we were unable to detect JNK activation in response to serum addition (Figure S2D) and it remains to be determined whether DGCR8 is phosphorylated by JNK in response to other stimuli, such as UV stress.

DGCR8 Phosphorylation Increases Microprocessor Levels by Increasing DGCR8 Protein Stability

To further test the correlation between DGCR8 phosphorylation and the observed DGCR8 protein levels (Figure 1F), we treated HeLa cells transfected with our FH-DGCR8 constructs with calyculin A, a serine/threonine phosphatase inhibitor. Upon calyculin A treatment, we observed ~2.3-fold and 1.7-fold higher levels of WT-FH-DGCR8 and Myc-Drosha (Figure 3A, Cal versus D lanes), respectively, as would be expected if indeed increased phosphorylation stabilizes DGCR8 (DGCR8 and Drosha levels are known to correlate since DGCR8 stabilizes Drosha protein; Han et al., 2009; Triboulet et al., 2009). Mut23 yielded DGCR8 levels similar to those observed for the WT (relative to the glyceraldehyde 3-phosphate dehydrogenase [GAPDH] loading control) in untreated cells, and Mim23 showed similar levels relative to WT in calyculin A-treated cells. Neither of the mutated constructs exhibited increased protein levels upon calyculin A treatment (Figure 3A). This result reconfirms that we have identified most, if not all, of the relevant phosphosites responsible for

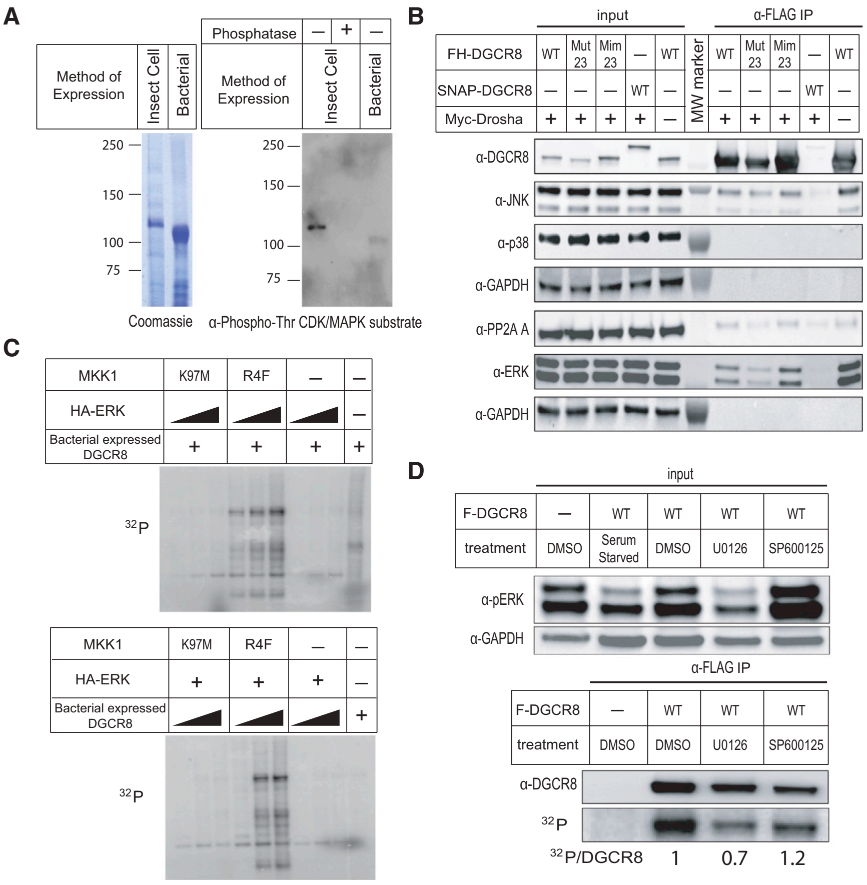

Figure 2. DGCR8 Is Targeted by ERK/MAPKs In Vivo

(A) Recombinant DGCR8 purified from baculovirus-infected insect cells, but not from *E. coli*, is recognized by an anti-Phospho-Thr CDK/MAPK substrate antibody. Left: Coomassie-stained SDS-PAGE of affinity-purified proteins. Right: immunoblot of the same affinity-purified protein samples (either phosphatase treated or untreated) probed with an anti-CDK/MAPK substrate antibody.

(B) Selected MAPKs can be immunopurified with MCs. HEK 293T cells were transiently transfected with vectors expressing GFP, Myc-Drosha, and either an empty vector or WT-FH-DGCR8, Mut23-FH-DGCR8, Mim23-FH-DGCR8, or WT-SNAP-DGCR8. Immunoblots of anti-FLAG immunoprecipitated MCs were probed for ERK, p38, or JNK MAPKs, as well as for PP2A A and GAPDH.

(C) DGCR8 can be phosphorylated by ERKs in vitro. ^{32}P-exposed gel images of ERK in vitro kinase assays. HA-ERK was immunoprecipitated from HEK 293T cells that had been transfected with either GFP alone (as a negative control [–]) or HA-ERK together with its upstream kinase MKK1-K97M (kinase dead), MKK1-R4F (constitutively active), or GFP. Immunoprecipitated ERK was incubated with bacterially expressed DGCR8. The top gel shows constant DGCR8 substrate levels (7.5 μl) with varying kinase levels (2, 5, or 10 μl immunoprecipitate), and the bottom gel shows increasing substrate levels (0, 7.5, 15 μl) with constant immunoprecipitated ERK levels (7.5 μl). The final lane of each gel shows the control immunoprecipitate incubated with DGCR8 using the highest levels of control immunoprecipitate (top gel) or DGCR8 (bottom gel).

(D) ERKs can phosphorylate DGCR8 in vivo. Strain 1 HeLa Flp-In cells stably expressing WT-F-DGCR8 or an empty vector were serum starved overnight and treated for 2 hr with DMSO control, U0126 (MEK1/2 inhibitor), or SP600125 (JNK inhibitor). Cells were then metabolically labeled with $^{32}PO_4$ upon serum addition for 4 hr. Total cell lysates (input) were probed for p-ERK in the upper immunoblots. Anti-FLAG immunoprecipitates were probed for DGCR8 and the ^{32}P signal was assessed in the lower immunoblots. Numbers indicate the amount of ^{32}P normalized to the DGCR8 signal.

See also Table S3 and Figure S2.

increasing DGCR8 protein levels. More importantly, we conclude that increased phosphorylation of DGCR8 leads to increased protein levels.

To corroborate the effect of multisite phosphorylation on DGCR8 protein levels, we expressed in HEK 293T cells constructs containing subsets of residues mutated to either prevent (Mut23 and Mut14 have 23 and 14 sites mutated, respectively) or mimic (Mim11 and Mim23 have 11 and 23 sites mutated, respectively; Table S2) phosphorylation. We examined the protein and mRNA levels for these constructs via immunoblots of cellular lysates and northern blot analyses of total RNA prepared from these cells, respectively (Figure 3B, left, with quantitations on the right). Increased DGCR8 protein levels were observed as the number of residues available for phosphorylation or mimicking phosphorylation increased, whereas DGCR8 mRNA levels remained constant for all constructs. Additional mutants with single sites or clusters of sites mutated to prevent or mimic phosphorylation were examined, but they exhibited no obvious phenotypes (data not shown). Thus, the multisite phosphorylation of DGCR8 appears to regulate DGCR8 stability in a graded fashion, rather than phosphorylation exceeding a threshold beyond which DGCR8 stability is changed in a sharp, switch-like manner or a single phosphosite being the sole regulator of protein stability. As previously reported (Han et al., 2009; Triboulet et al., 2009), increased DGCR8 protein levels correlated with the levels of tagged, cotransfected Drosha. Indeed, increased Drosha protein levels were independent of Drosha mRNA levels (Figure 3B). Comparable changes in Mut23-DGCR8 versus Mim23-DGCR8 protein levels were also seen in transfected HeLa cells (Figure 3A, lanes labeled D [DMSO]), indicating a general rather than a cell-specific effect.

To confirm that phosphorylation of DGCR8 increases its stability, we generated two different strains of HeLa cells that stably express F-DGCR8 constructs (F-DGCR8 Mut23, WT, Mim23, or the empty vector) from the same chromosomal site within each strain (Flp-In cells; see the Supplemental Experimental Procedures; WT-F-DGCR8 and the empty vector from strain 1 were used for Figure 2D above). These two cell lines exhibit levels of exogenous DGCR8 that are ~15- to 45-fold higher than endogenous levels, and Drosha protein levels that are ~2- to 5-fold

Cell PRESS
Open ACCESS

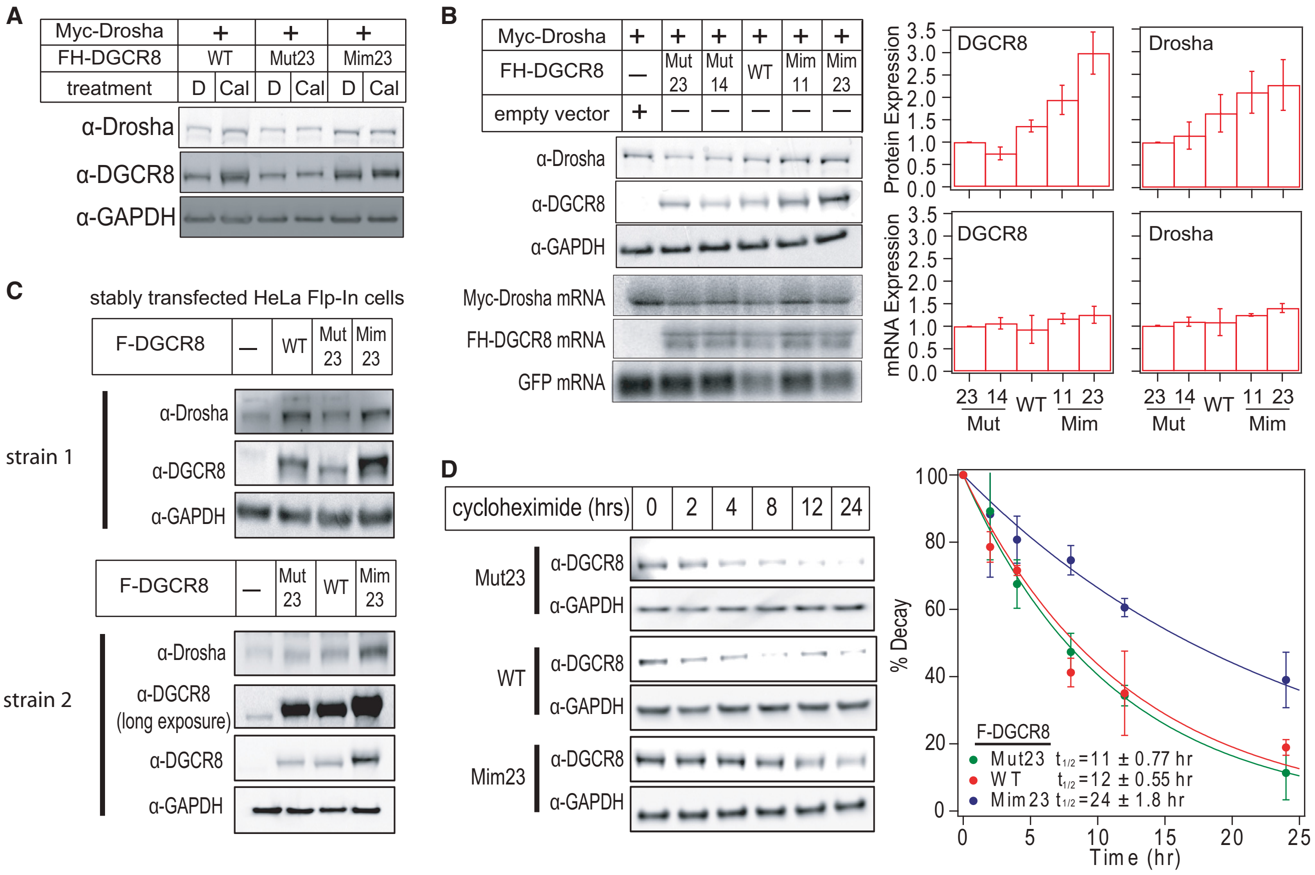

Figure 3. Expression of the Phosphomimetic Increases DGCR8 Stability

(A) Inhibition of phosphatase stabilizes DGCR8 protein. HeLa cells were transiently transfected with vectors expressing GFP, Myc-Drosha, and either WT-FH-DGCR8, Mut23-FH-DGCR8, or Mim23-FH-DGCR8. After 24 hr, cells were treated with either DMSO (D, control) or calyculin A (Cal) for 20 min before harvesting. Immunoblotting for Drosha, DGCR8, and GAPDH was performed on total cell lysates.

(B) In transiently transfected HEK 293T cells, increased DGCR8 protein levels are observed as the number of residues available for phosphorylation or mimicking phosphorylation increases. HEK 293T cells were transiently transfected with vectors expressing GFP, Myc-Drosha, and either an empty vector or WT-FH-DGCR8, Mut23-FH-DGCR8, Mut14-FH-DGCR8, Mim11-FH-DGCR8, or Mim23-FH-DGCR8. Equal portions of cells were used for immunoblotting and making RNA preparations for northern blots. Northern blots were probed with antisense oligonucleotides specific for GFP or for the tag sequences for Drosha (Myc) and DGCR8 (FLAG). Quantitative analyses of protein levels from immunoblots (top) and RNA levels from northern blots (bottom), normalized to Mut23 protein and mRNA levels, respectively, are shown on the right. Values represent mean ± SEM, n = 5.

(C) Stably transfected HeLa cells also show increased DGCR8 protein levels as the number of residues available for phosphorylation or mimicking of phosphorylation increases. Immunoblots showing protein levels in total cell lysates from two isogenic strains of HeLa Flp-In cells stably expressing WT-F-DGCR8, Mut23-F-DGCR8, Mim23-F-DGCR8, or an empty vector.

(D) Increased DGCR8 protein levels are due to differences in protein stability. Strain 2 isogenic HeLa Flp-In cells stably expressing WT-F-DGCR8, Mut23-F-DGCR8, or Mim23-F-DGCR8 were treated with 100 μg/ml cycloheximide. Cells were harvested at 0, 2, 4, 8, 12, and 24 hr. Immunoblots were performed on total cell lysates to monitor DGCR8 decay. Quantitative analyses are shown on the right. Values are normalized to protein levels at 0 hr and represent mean ± SEM, n = 3. Fitting the curves with single-exponential decays (amplitude fixed at 1 and baseline at 0) generated the following $t_{1/2}$ values (mean ± SD): Mim23 = 24 ± 1.8 hr, Mut23 = 11 ± 0.77 hr, WT = 12 ± 0.55 hr.

See also Figure S3.

higher. Although the protein levels of Mim23 relative to Mut23 are 2- to 3-fold higher in both cell lines (Figure 3C), the level of WT-DGCR8, which retains the ability to respond to various signaling cascades, varies between the two cell lines: WT-F-DGCR8 levels are comparable to those of Mim23 in strain 1 and those of Mut23 in strain 2. This is likely due to variations in activated signaling cascades in different cell lines and is consistent with the idea that DGCR8 levels are regulated by phosphorylation.

Then, we used these stably expressing HeLa cell lines to verify that the observed differences in phosphomutant and phosphomimetic DGCR8 protein levels were due to changes in protein stability by measuring protein decay after treating the cells with the translation inhibitor cycloheximide. In strain 2, Mim23-F-DGCR8 had a half-life of ~22 hr, whereas WT-F-DGCR8 and Mut23-F-DGCR8 had half-lives of ~11 hr (Figure 3D). In strain 1, where WT levels were closer to those of Mim23, decay rates after cycloheximide treatment were

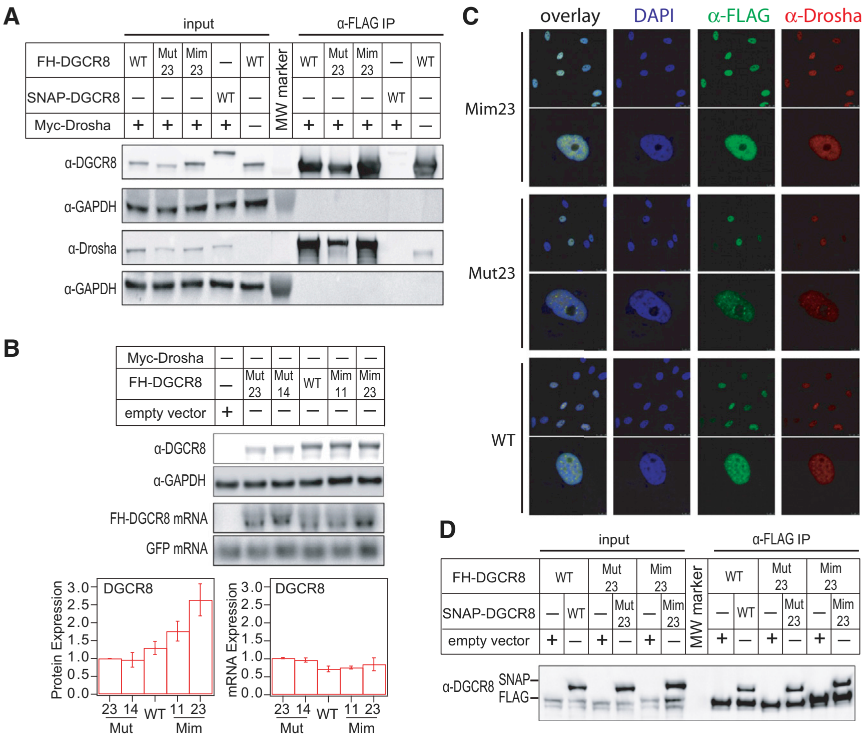

Figure 4. Phosphorylation of DGCR8 Does Not Alter Its Localization or Ability to Associate with Drosha or Itself

(A) Drosha is not differentially coimmunoprecipitated with the DGCR8 phosphosite mutants. HEK 293T cells were transiently transfected with vectors expressing GFP, Myc-Drosha, and either an empty vector or WT-FH-DGCR8, Mut23-FH-DGCR8, Mim23-FH-DGCR8, or WT-SNAP-DGCR8. Immunoblots of anti-FLAG immunoprecipitated MCs were probed for Drosha, DGCR8, and GAPDH. The anti-DGCR8 panel is reproduced from Figure 2B.

(B) The stabilization of DGCR8 protein levels is independent of MC formation. HEK 293T cells were transiently transfected with GFP and either an empty vector or vectors expressing WT-FH-DGCR8, Mut23-FH-DGCR8, Mut14-FH-DGCR8, Mim11-FH-DGCR8, or Mim23-FH-DGCR8. Equal portions of cells were used for immunoblotting and making RNA preparations for northern blots. Northern blots were probed with oligonucleotides specific for GFP or the tag sequence in the case of DGCR8 (FLAG). Quantitative analyses of protein expression levels from immunoblots and RNA expression levels from northern blots, normalized to Mut23 protein and mRNA levels, respectively, are shown below. Values represent mean ± SEM, n = 6.

(C) Phosphomutant and phosphomimetic DGCR8 do not show altered cellular localization. Immunofluorescence of isogenic HeLa Flp-In cells stably expressing WT-FLAG-DGCR8, Mut23-FLAG-DGCR8, or Mim23-FLAG-DGCR8. The subnuclear distribution of all DGCR8 constructs, WT-FLAG-DGCR8, Mut23-FLAG-DGCR8, and Mim23-FLAG-DGCR8 was variable, sometimes exhibiting foci and sometimes localizing to nucleoli.

(D) Phosphomutant and phosphomimetic DGCR8s, like WT DGCR8, self-associate. HEK 293T cells were transiently transfected with vectors expressing either WT-FH-DGCR8, Mut23-FH-DGCR8, or Mim23-FH-DGCR8, and either WT-SNAP-DGCR8, Mut23-SNAP-DGCR8, or Mim23-SNAP-DGCR8, as well as GFP. DGCR8 was isolated via anti-FLAG immunoprecipitation. SNAP- and FH-tagged versions of DGCR8 can be distinguished by migration shifts.

examined over a more limited number of time points, and WT-F-DGCR8 showed stability similar to that observed for the Mim23 construct (Figure S3). Together, these data argue that phosphorylation stabilizes DGCR8 protein, which results in increased MC levels.

The Increased Stabilization of Phosphomimetic DGCR8 Is Not Due to Altered Localization or Ability to Associate with Drosha or Itself

Increased DGCR8 protein stability could result primarily from protein phosphorylation or secondarily from phosphorylation-induced changes in association with Drosha, ability to self-associate, or cellular localization. DGCR8 phosphorylation does not appear to significantly affect interactions with Drosha. Mim23-DGCR8, WT- DGCR8, and Mut23-DGCR8 all coimmunoprecipitated cotransfected Drosha protein comparably, with the amount of associated Drosha being proportional to the amount of DGCR8 (Figure 4A). Consistent with the fact that none of the phosphosites are in the segment of DGCR8 required for association with Drosha (Yeom et al., 2006), we conclude that the increased protein stability of phosphorylated DGCR8 is not due to differential interactions with Drosha. Figure 4A also shows that DGCR8 can coimmunoprecipitate considerably more Drosha than is present endogenously, suggesting that endogenous Drosha levels are not high enough to bind the overexpressed DGCR8 stoichiometrically. However, Mim23-DGCR8 shows increased protein levels compared with WT- DGCR8 or Mut23-DGCR8 when expressed either transiently in HEK 293T cells (Figure 4B) or stably in strain 1 or 2 HeLa cells (Figure 3C), even though Drosha is not overexpressed and therefore is not available to bind DGCR8 stoichiometrically in either cellular context. Therefore, we further conclude that, unlike Drosha protein, which is stabilized by complex formation with DGCR8 (Han et al., 2009), the stabilizing effect of phosphorylation on DGCR8 protein is independent of MC formation.

All 23 DGCR8 phosphosites appear in the N terminus, which is required for nuclear localization (Yeom et al., 2006) and for the ability of DGCR8 to homodimerize (Faller et al., 2007). We performed immunofluorescence studies on strain 2 HeLa cells stably expressing F-DGCR8 constructs. As was observed for WT-F-DGCR8, both the Mut23 and Mim23 proteins localized exclusively to the nucleus (Figure 4C). Phosphorylation also did not significantly alter DGCR8's ability to self-associate. As reported previously (Han et al., 2004), WT-FH-DGCR8 coimmunoprecipitated a differently tagged WT DGCR8 construct (SNAP-DGCR8) (Figure 4D). Mut23-FH and Mim23-FH coimmunoprecipitated SNAP-tagged Mut23 and Mim23, respectively, to the same extent (Figure 4D).

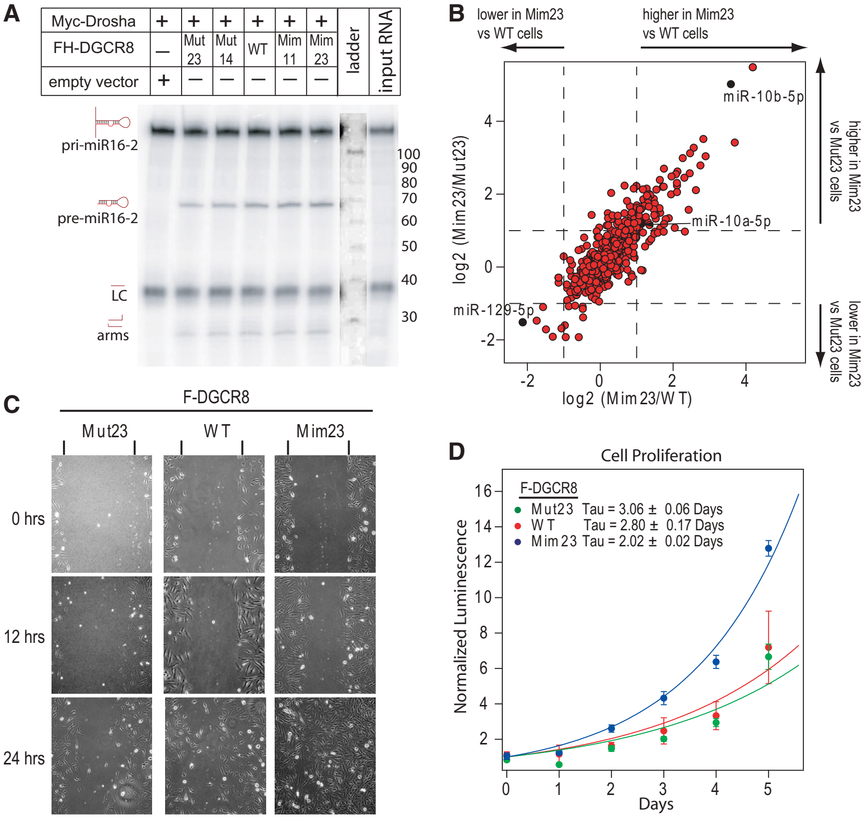

Figure 5. Expression of Phosphomimetic DGCR8 Leads to a Progrowth MiRNA Profile and Increases Cell Proliferation and In Vitro Scratch Closure Rates

(A) MCs incorporating phosphomutant or phosphomimetic DGCR8 do not show altered specific pri-miRNA processing activity. In vitro pri-miRNA-processing assays were performed by incubating ^{32}P body-labeled pri-miR16-2 and a short (35 nt) stable RNA, which functions as a loading control (LC), with immunoprecipitated MCs from equal concentrations of lysates from HEK 293T cells that had been transiently transfected with GFP, Myc-Drosha, and either an empty vector or vectors expressing WT-FH-DGCR8, Mut23-FH-DGCR8, Mut14-FH-DGCR8, Mim11-FH-DGCR8, or Mim23-FH-DGCR8. The input RNAs are shown in the far-right lane. Contrast has been adjusted separately on the ladder lane. Ladder sizes are indicated on the right in nucleotides.

(B) Cells expressing phosphomimetic DGCR8, compared with WT or phosphomutant DGCR8, show a progrowth miRNA profile. Next-generation sequencing was used to profile levels of small RNAs from strain 2 isogenic HeLa Flp-In cells stably expressing Mim23-F-DGCR8, WT-F-DGCR8, or Mut23-F-DGCR8. Each dot represents (for an individual mature miRNA) the average (n = 3) log_2 relative expression in Mim23-F-DGCR8 over Mut23-F-DGCR8 cells versus the log_2 relative expression in Mim23-F-DGCR8 over WT-F-DGCR8 cells. Dotted lines are shown at 1 and −1, corresponding to a 2-fold change up or down, respectively. Thus, a miRNA with a >2-fold up or down change in the Mim23 sample relative to both the Mut23 and WT sample will be in the upper-right or lower-left quadrant. Error bars are omitted for simplicity.

(C) Cells expressing phosphomimetic DGCR8, compared with WT or phosphomutant DGCR8, show a faster in vitro scratch closure rate. Strain 2 isogenic HeLa Flp-In cells stably expressing WT-F-DGCR8, Mut23-F-DGCR8, or Mim23-F-DGCR8 were plated at 500,000 cells per 10 cm plate. After settling overnight, cells were serum starved 24 hr. Then, a 200 μl pipette was used to create a scratch before readdition of serum. Cells were photographed every 12 hr.

(D) Cells expressing phosphomimetic DGCR8, compared with WT or phosphomutant DGCR8, show increased cell proliferation rates. Strain 2 isogenic HeLa Flp-In cells stably expressing WT-F-DGCR8, Mut23-F-DGCR8, or Mim23-F-DGCR8 were plated at 200 cells per well in a 96-well plate. After settling overnight, cells were serum starved 24 hr. Upon serum addition, cell proliferation was measured every 24 hr for 5 days using Cell Titer Glo reagent. Plots of luminescence normalized to average luminescence on day 1 (mean ± SEM, n = 6) versus time were fit to a single-exponential growth equation (value at time 0 fixed at 1), which determined the doubling rates (τ) of (mean ± SD).

See also Figures S4 and S5 and Tables S4–S6.

MCs Containing Phosphomutant or Phosphomimetic DGCR8 Are Not Altered in Specific Processing Activity

To test whether Drosha's catalytic activity is altered by association with phosphorylated DGCR8, we incubated equal volumes of immunoprecipitated MCs from transiently transfected HEK 293T cell cultures with body-labeled, in vitro-transcribed pri-miRNA substrates. Processing activity, as measured by the yield of pre-miRNA relative to the loading control, correlated with MC expression levels in these cells, i.e., it was lower than in the WT for MCs containing Mut23, and higher for MCs containing Mim23 (Figures 5A and S4A). Note that these reactions contained different amounts of MC, since DGCR8 concentrations in immunoprecipitates are proportional to lysate concentrations (Figure S4B).

This in vitro assay detects primarily the activity of MCs that are minimally composed of Drosha and DGCR8, since (1) interacting proteins were not cotransfected and therefore were not present in quantities stoichiometric to Drosha and DGCR8, and (2) the immunoprecipitates were washed with high salt concentrations (250 mM) to minimize the copurification of other factors. Nonetheless, the immunoprecipitated MCs were probed for two of the best-known MC-interacting factors (the p68 and p72 helicases; Figure S4C), other factors known to regulate pri-miRNA cleavage (KHSRP, SRp20, RNH1, Ars2, and FUS), and the downstream miRNA biogenesis factor Exportin 5 (data not shown). Although all were present at higher levels in the immunoprecipitates than in the nonspecific controls, their levels in each immunoprecipitate were proportional to the amount of DGCR8, indicating that there were no significant differences in cofactor association. These results argue that DGCR8 phosphorylation does not significantly alter the specific processing activity of individual minimal MCs into which DGCR8 is incorporated.

Expression of Phosphomimetic DGCR8 Generates a Progrowth miRNA Expression Profile and Increases Cell Proliferation

Since the specific activities of individual MCs were not significantly affected by the incorporation of Mut23 or Mim23 DGCR8, we tested whether the differences in protein levels observed when these DGCR8 mutants were stably expressed led to differences in miRNA biogenesis. We used next-generation sequencing to profile small RNAs from strain 2 HeLa cells stably expressing Mim23-DGCR8, Mut23-DGCR8, or WT-F-DGCR8 (Figure 5B). It should be noted that although DGCR8 is overexpressed in these cells, its level was observed by immunofluorescence to be uniform from cell to cell due to stable transformation. Moreover, it has been reported that high MC performance can be achieved even when MC levels significantly exceed cellular levels of pri-miRNAs (Barad et al., 2012). We normalized individual miRNA read counts by the total number of miRNA reads per sample and then averaged the $\log_2$-fold changes over the three biological replicates (Figures S5A and S5B). This strategy yielded average fold changes for mature miRNAs that were consistent with values obtained by TaqMan quantitative PCR (Figure S5C). The average $\log_2$-fold change for cells expressing the mimetic versus the mutant DGCR8 was 0.38 ± 0.035 (corresponding to a fold change of 1.28–1.34), whereas the average $\log_2$-fold change for the mimetic over the WT was 0.32 ± 0.031 (corresponding to a fold change of 1.22–1.27). The 2- to 3-fold differences in cellular protein levels for the DGCR8 WT and mutants would be expected to alter global levels of mature miRNAs if DGCR8 were limiting for miRNA biogenesis. However, given the complexity in normalization of RNA sequencing values (Dillies et al., 2012; Robinson and Oshlack, 2010), we do not believe the small increase in global miRNA abundance is significant. This conclusion is consistent with previous work showing that other components of the miRNA biogenesis pathway are limiting (Diederichs and Haber, 2007; Yi et al., 2005) and with models of DGCR8 haploinsufficiency that show effects only on selected miRNAs (Schofield et al., 2011; Stark et al., 2008).

Because miRNA biogenesis is highly regulated, certain miRNAs appeared to be more sensitive to MC levels and/or the phosphorylation status of DGCR8. Of 616 miRNAs, 75 showed a >2-fold increase in the Mim23 cell line relative to both the WT- and Mut23-expressing cell lines (upper-right quadrant of Figure 5B; Tables S4–S6). Of the 75 upregulated miRNAs, the most abundant (those with the highest total read count) were miR-10a-5p and miR-10b-5p. Only seven miRNAs showed a >2-fold decrease in the Mim23 cell line (lower-left quadrant of Figure 5B; Table S5). Of those seven, the most abundant was miR-129-5p. The miR-10 family of miRNAs is deregulated in several types of cancer (Lund, 2010). MiR-10b is highly expressed in metastatic breast cancer cells, where it positively regulates cell migration and invasion (Ma et al., 2007), and the level of miR-10a affects the capacity of cells to undergo oncogenic transformation (Ørom et al., 2008). MiR129-5p, on the other hand, has been reported to have an antiproliferative effect by targeting Cdk6 (Wu et al., 2010). Neither miR-10b nor miR-129-1 was processed with significantly different efficiency by MCs containing DGCR8 mutants (Figure S4A). Therefore, the in vivo sensitivity of mature miR-10b and miR-129 levels to DGCR8 protein level or phosphorylation status could be due to differential interactions with some protein cofactor that regulates processing or to indirect effects of DGCR8 phosphorylation.

The upregulation of the tumorigenic, progrowth miR10a and miR10b, and downregulation of the antiproliferative miR129-5p seen in the Mim23-expressing cells would be predicted to alter cell growth and invasion properties. Indeed, in an in vitro scratch assay, Mim23-expressing cells exhibited faster rates of scratch closure compared with Mut23- and WT-expressing cells (Figure 5C). HeLa cells expressing Mim23-F-DGCR8 showed higher doubling rates than those expressing Mut23-DGCR8 or WT-F-DGCR8 (Figure 5D). The increased proliferation rate of Mim23-expressing cells, which show higher MC levels than WT-DGCR8-expressing cells, is consistent with reports that DGCR8 knockout (Chapnik et al., 2012; Chen et al., 2012; Steiner et al., 2011; Wang et al., 2007; Yi et al., 2009) or sequestration (Sellier et al., 2013) leads to cell-cycle defects or apoptosis. Thus, the phosphorylation of DGCR8 may be a means by which the MC senses cell-cycle regulation cues, leading to cell proliferation.

DISCUSSION

We have investigated the impact of protein modification on the critical miRNA biogenesis factor DGCR8. Our results demonstrate that multisite phosphorylation regulates DGCR8 protein stability, thereby raising MC levels (Figure 3), changing the mature miRNA profile of the cell, and increasing cell proliferation and migration (Figure 5). Moreover, we find that the accumulation of multiple phosphorylations creates a graded response in DGCR8 stability (Figure 3B), rather than a single phosphosite modulating DGCR8 protein. The modifications are introduced at least in part by ERK/MAPKs in vivo (Figure 2), linking control of miRNA biogenesis to extracellular cues. Because miRNAs have been implicated in a myriad of biological functions and disease processes, it is not surprising that their biogenesis is regulated at many levels. Our findings provide important mechanistic insights into the functional and biological consequences of DGCR8 phosphorylation.

Previously, multisite phosphorylation of proteins was found to regulate protein function in either a graded fashion, as we have found, or by a switch-like response (Nash et al., 2001; Serber and Ferrell, 2007; Strickfaden et al., 2007). The levels of DGCR8 are tightly regulated by two autoregulatory feedback mechanisms: one in which the microprocessor cleaves *Dgcr8* mRNA (Han et al., 2009; Kadener et al., 2009; Triboulet et al., 2009) and one in which the levels of DGCR8 adjust to those of pri-miRNA substrates (Barad et al., 2012). Multisite phosphorylation represents yet another possible mechanism to ensure tight control over microprocessor levels to keep them in an optimal range for activity.

Modulation of protein stability by phosphorylation is becoming a common theme in biology, and examples of crosstalk between phosphorylation and ubiquitin-mediated degradation of proteins are increasingly being reported (Hunter, 2007). Within the miRNA biogenesis pathway itself, changes in the PTMs of miRNA processing enzymes and their dsRNA-binding partners, effected

by cell-signaling pathways, have been reported for TRBP2 and Drosha phosphorylation, and for DGCR8 and Drosha acetylation (Paroo et al., 2009; Tang et al., 2010, 2011, 2013; Wada et al., 2012). Exactly how phosphorylation confers increased stability to DGCR8 or TRBP2 is not yet known. The mapped DGCR8 phosphosites all exist within regions that are known to be important for nuclear localization or homodimerization, yet neither of these properties of DGCR8 was affected by DGCR8 phosphorylation (Figures 4C and 4D). Drosha protein levels also did not appear to be important for stabilization of phosphomimetic-DGCR8 (Figure 4B). It has been suggested that DGCR8 might exist in complexes with endonucleases and proteins other than Drosha (Macias et al., 2012; Shiohama et al., 2007). The different interacting partners of phosphorylated and unphosphorylated DGCR8 warrant future studies to determine whether an unknown protein binding partner interacts preferentially with one form or another. Such studies could also identify other kinases acting on DGCR8, and could elucidate whether DGCR8 is a target of ubiquitin-mediated degradation by identifying a ubiquitin E3-ligase that preferentially binds the unphosphorylated form, leading to DGCR8 ubiquitination and degradation. DGCR8 shows several RXXL motifs (i.e., potential APC/C-recognized destruction boxes).

DGCR8 was recently shown to be the target of caspase 3-mediated cleavage (Gong et al., 2012). Significant crosstalk between phosphorylation and caspase cleavage has been documented (Dix et al., 2012) and phosphorylation of DGCR8 at S397 (the amino acid immediately C-terminal to the caspase-cleaved scissile bond) is predicted to interfere with caspase cleavage (Tözsér et al., 2003). However, the observed differences in protein stability among our WT-DGCR8, Mim23-DGCR8, and Mut23-DGCR8 constructs cannot be explained solely by differences in susceptibility to caspase-mediated cleavage, as we observed little, if any, caspase 3 activity (determined by blotting for cleaved Poly ADP ribose polymerase) in either our transiently transfected or stable cell lines (data not shown). Additionally, after incubating immunoprecipitated WT-FH-DGCR8, Mut23-FH-DGCR8, or Mim23-FH-DGCR8 from HEK 293T cells with recombinant caspase 3 or activating caspases in the various DGCR8-expressing cells with etoposide, we observed similar extents of DGCR8 cleavage by caspase for all three constructs (data not shown). These observations preliminarily indicate that phosphorylation does not regulate caspase cleavage of DGCR8.

We have demonstrated that phosphorylation driven by ERK/MAPKs regulates MC levels. ERKs are mitogenic kinases that drive cellular proliferation upon signaling stimulation mainly by extracellular growth factors. Accordingly, HeLa cells stably expressing Mim23-F-DGCR8 showed increased cell proliferation and invasion relative to Mut23-F-DGCR8 and WT-F-DGCR8-expressing cells, and the progrowth miR-10a and miR-10b were significantly enhanced (Figure 5). The phosphorylation of DGCR8 by ERK1 and ERK2 during the cell cycle and/or upon extracellular stimulation may thus be one way in which the MC senses regulatory cues to promote cell proliferation. This finding is similar to observations regarding TRBP2 phosphorylation by ERKs (Chakravarthy et al., 2010; Paroo et al., 2009). Since DGCR8 and TRBP2 respond comparably to ERK/MAPKs, we investigated whether expression of phosphomimetic or phosphomutant DGCR8 might affect TRBP2 protein levels, but we found no evidence for such a feedback loop between the nuclear and cytoplasmic arms of the miRNA biogenesis pathway (data not shown). However, it will be important to further characterize the signaling pathways that target the MC and miRNA biogenesis in general, given that many drugs inhibit kinases and thus have the potential to reprogram miRNA expression.

DGCR8 is an integral component of the cellular microprocessor. The phosphorylation events we have identified allow the cell to respond to extracellular cues, such as the mitogens that stimulate ERK1 and ERK2, and appear comparable to the digital data input that a computer microprocessor receives. Changes in DGCR8 stability induced by phosphorylation events likewise result in an altered digital output that affects cellular growth rates.

EXPERIMENTAL PROCEDURES

Plasmids

pFLAG/HA-DGCR8 (pFH-DGCR8) and pcDNA4/TO/cmycDrosha (Landthaler et al., 2004) were purchased from Addgene. Details on how pCS3-MT-MycDrosha; all WT, mutant, and mimetic FH-DGCR8 constructs (for transient transfections); pSNAP-DGCR8 (for transient transfections); pcDNA5/FRT-F-DGCR8 (for stable transfections); pET28a-DGCR8 (for bacterial expression); and pFast-Bac1-HisDGCR8 (for baculovirus expression) were cloned from the original pFH-DGCR8 and pcDNA4/TO/cmycDrosha plasmids are provided in the Supplemental Experimental Procedures. The sequences of each mutant and mimetic construct are given in Table S2. pGFPmax was used for two reasons: (1) it allowed determination of transfection efficiency and (2) it provided a loading control for the northern blots. pcDNA3 was used as the empty vector control.

Mammalian Cell Assays

Details on cell culture, transfections, cell lysis, metabolic labeling, development of stable cell lines, and proliferation assays are provided in the Supplemental Experimental Procedures.

Immunoprecipitations, Blots, Immunofluorescence, and In Vitro Processing Assays

Immunoprecipitations and immunoblots were performed according to Pimienta et al. (2007) and Pimienta et al. (2011), respectively. Immunofluorescence, northern blots, and in vitro processing assays were performed according to Pawlicki and Steitz (2008). Detailed protocols with modifications are provided in the Supplemental Experimental Procedures.

SUPPLEMENTAL INFORMATION

Supplemental Information includes Supplemental Experimental Procedures, five figures, one data file, and six tables and can be found with this article online at http://dx.doi.org/10.1016/j.celrep.2013.10.017.

ACKNOWLEDGMENTS

We thank J. Pawlicki from the J.A.S. laboratory and C. Mader from the Koleske laboratory for experimental protocols and reagents. We are grateful to S. Reinhardt and E. Frankel for help creating DGCR8 mutants, B. Turk for providing ERK and MKK1 plasmids, A. Rongvaux for discussions and reagents for apoptosis assays, the laboratory of T. Walther for providing access to software for visualizing MS data, and the W.M. Keck Biotechnology Resource Laboratory for running samples. We thank K. Tycowski, A. Vilborg, J. Withers, J. Brown, and W. Moss for critical readings of the manuscript and A. Miccinello for editorial help. This work was supported by grant GM026154 from the NIH. J.A.S. is an investigator of the Howard Hughes Medical Institute. K.M.H. was an HHMI Fellow of the Damon Runyon Cancer Research Foundation.

Received: September 30, 2012
Revised: July 3, 2013
Accepted: October 9, 2013
Published: November 14, 2013

REFERENCES

Barad, O., Mann, M., Chapnik, E., Shenoy, A., Blelloch, R., Barkai, N., and Hornstein, E. (2012). Efficiency and specificity in microRNA biogenesis. Nat. Struct. Mol. Biol. *19*, 650–652.

Barr, I., Smith, A.T., Senturia, R., Chen, Y., Scheidemantle, B.D., Burstyn, J.N., and Guo, F. (2011). DiGeorge critical region 8 (DGCR8) is a double-cysteine-ligated heme protein. J. Biol. Chem. *286*, 16716–16725.

Chakravarthy, S., Sternberg, S.H., Kellenberger, C.A., and Doudna, J.A. (2010). Substrate-specific kinetics of Dicer-catalyzed RNA processing. J. Mol. Biol. *404*, 392–402.

Chapnik, E., Sasson, V., Blelloch, R., and Hornstein, E. (2012). Dgcr8 controls neural crest cells survival in cardiovascular development. Dev. Biol. *362*, 50–56.

Chen, Z., Wu, J., Yang, C., Fan, P., Balazs, L., Jiao, Y., Lu, M., Gu, W., Li, C., Pfeffer, L.M., et al. (2012). DiGeorge syndrome critical region 8 (DGCR8) protein-mediated microRNA biogenesis is essential for vascular smooth muscle cell development in mice. J. Biol. Chem. *287*, 19018–19028.

Chendrimada, T.P., Gregory, R.I., Kumaraswamy, E., Norman, J., Cooch, N., Nishikura, K., and Shiekhattar, R. (2005). TRBP recruits the Dicer complex to Ago2 for microRNA processing and gene silencing. Nature *436*, 740–744.

Cox, J., Neuhauser, N., Michalski, A., Scheltema, R.A., Olsen, J.V., and Mann, M. (2011). Andromeda: a peptide search engine integrated into the MaxQuant environment. J. Proteome Res. *10*, 1794–1805.

Denli, A.M., Tops, B.B., Plasterk, R.H., Ketting, R.F., and Hannon, G.J. (2004). Processing of primary microRNAs by the Microprocessor complex. Nature *432*, 231–235.

Dephoure, N., Zhou, C., Villén, J., Beausoleil, S.A., Bakalarski, C.E., Elledge, S.J., and Gygi, S.P. (2008). A quantitative atlas of mitotic phosphorylation. Proc. Natl. Acad. Sci. USA *105*, 10762–10767.

Diederichs, S., and Haber, D.A. (2007). Dual role for argonautes in microRNA processing and posttranscriptional regulation of microRNA expression. Cell *131*, 1097–1108.

Dillies, M.A., Rau, A., Aubert, J., Hennequet-Antier, C., Jeanmougin, M., Servant, N., Keime, C., Marot, G., Castel, D., Estelle, J., et al.; on behalf of The French StatOmique Consortium. (2012). A comprehensive evaluation of normalization methods for Illumina high-throughput RNA sequencing data analysis. Brief. Bioinform. Published online September 17, 2012. http://dx.doi.org/10.1093/bib/bbs046.

Dix, M.M., Simon, G.M., Wang, C., Okerberg, E., Patricelli, M.P., and Cravatt, B.F. (2012). Functional interplay between caspase cleavage and phosphorylation sculpts the apoptotic proteome. Cell *150*, 426–440.

Faller, M., Matsunaga, M., Yin, S., Loo, J.A., and Guo, F. (2007). Heme is involved in microRNA processing. Nat. Struct. Mol. Biol. *14*, 23–29.

Garai, A., Zeke, A., Gógl, G., Törő, I., Fördős, F., Blankenburg, H., Bárkai, T., Varga, J., Alexa, A., Emig, D., et al. (2012). Specificity of linear motifs that bind to a common mitogen-activated protein kinase docking groove. Sci. Signal. *5*, ra74.

Gong, M., Chen, Y., Senturia, R., Ulgherait, M., Faller, M., and Guo, F. (2012). Caspases cleave and inhibit the microRNA processing protein DiGeorge Critical Region 8. Protein Sci. *21*, 797–808.

Gregory, R.I., Yan, K.P., Amuthan, G., Chendrimada, T., Doratotaj, B., Cooch, N., and Shiekhattar, R. (2004). The Microprocessor complex mediates the genesis of microRNAs. Nature *432*, 235–240.

Guo, H., Ingolia, N.T., Weissman, J.S., and Bartel, D.P. (2010). Mammalian microRNAs predominantly act to decrease target mRNA levels. Nature *466*, 835–840.

Haase, A.D., Jaskiewicz, L., Zhang, H., Lainé, S., Sack, R., Gatignol, A., and Filipowicz, W. (2005). TRBP, a regulator of cellular PKR and HIV-1 virus expression, interacts with Dicer and functions in RNA silencing. EMBO Rep. *6*, 961–967.

Han, J., Lee, Y., Yeom, K.H., Kim, Y.K., Jin, H., and Kim, V.N. (2004). The Drosha-DGCR8 complex in primary microRNA processing. Genes Dev. *18*, 3016–3027.

Han, J., Pedersen, J.S., Kwon, S.C., Belair, C.D., Kim, Y.K., Yeom, K.H., Yang, W.Y., Haussler, D., Blelloch, R., and Kim, V.N. (2009). Posttranscriptional crossregulation between Drosha and DGCR8. Cell *136*, 75–84.

Holt, L.J., Tuch, B.B., Villén, J., Johnson, A.D., Gygi, S.P., and Morgan, D.O. (2009). Global analysis of Cdk1 substrate phosphorylation sites provides insights into evolution. Science *325*, 1682–1686.

Hunter, T. (2007). The age of crosstalk: phosphorylation, ubiquitination, and beyond. Mol. Cell *28*, 730–738.

Iwasaki, Y.W., Kiga, K., Kayo, H., Fukuda-Yuzawa, Y., Weise, J., Inada, T., Tomita, M., Ishihama, Y., and Fukao, T. (2013). Global microRNA elevation by inducible Exportin 5 regulates cell cycle entry. RNA *19*, 490–497.

Kadener, S., Rodriguez, J., Abruzzi, K.C., Khodor, Y.L., Sugino, K., Marr, M.T., 2nd, Nelson, S., and Rosbash, M. (2009). Genome-wide identification of targets of the drosha-pasha/DGCR8 complex. RNA *15*, 537–545.

Landthaler, M., Yalcin, A., and Tuschl, T. (2004). The human DiGeorge syndrome critical region gene 8 and Its D. melanogaster homolog are required for miRNA biogenesis. Curr. Biol. *14*, 2162–2167.

Lund, A.H. (2010). miR-10 in development and cancer. Cell Death Differ. *17*, 209–214.

Ma, L., Teruya-Feldstein, J., and Weinberg, R.A. (2007). Tumour invasion and metastasis initiated by microRNA-10b in breast cancer. Nature *449*, 682–688.

Macias, S., Plass, M., Stajuda, A., Michlewski, G., Eyras, E., and Cáceres, J.F. (2012). DGCR8 HITS-CLIP reveals novel functions for the Microprocessor. Nat. Struct. Mol. Biol. *19*, 760–766.

Nash, P., Tang, X., Orlicky, S., Chen, Q., Gertler, F.B., Mendenhall, M.D., Sicheri, F., Pawson, T., and Tyers, M. (2001). Multisite phosphorylation of a CDK inhibitor sets a threshold for the onset of DNA replication. Nature *414*, 514–521.

Ohashi, R., Nakagawa, T., Watanabe, S., Kanellis, J., Almirez, R.G., Schreiner, G.F., and Johnson, R.J. (2004). Inhibition of p38 mitogen-activated protein kinase augments progression of remnant kidney model by activating the ERK pathway. Am. J. Pathol. *164*, 477–485.

Olsen, J.V., Blagoev, B., Gnad, F., Macek, B., Kumar, C., Mortensen, P., and Mann, M. (2006). Global, in vivo, and site-specific phosphorylation dynamics in signaling networks. Cell *127*, 635–648.

Ørom, U.A., Nielsen, F.C., and Lund, A.H. (2008). MicroRNA-10a binds the 5′UTR of ribosomal protein mRNAs and enhances their translation. Mol. Cell *30*, 460–471.

Paroo, Z., Ye, X., Chen, S., and Liu, Q. (2009). Phosphorylation of the human microRNA-generating complex mediates MAPK/Erk signaling. Cell *139*, 112–122.

Pawlicki, J.M., and Steitz, J.A. (2008). Primary microRNA transcript retention at sites of transcription leads to enhanced microRNA production. J. Cell Biol. *182*, 61–76.

Pimienta, G., Ficarro, S.B., Gutierrez, G.J., Bhoumik, A., Peters, E.C., Ronai, Z., and Pascual, J. (2007). Autophosphorylation properties of inactive and active JNK2. Cell Cycle *6*, 1762–1771.

Pimienta, G., Herbert, K.M., and Regan, L. (2011). A compound that inhibits the HOP-Hsp90 complex formation and has unique killing effects in breast cancer cell lines. Mol. Pharm. *8*, 2252–2261.

Robinson, M.D., and Oshlack, A. (2010). A scaling normalization method for differential expression analysis of RNA-seq data. Genome Biol. *11*, R25.

Schofield, C.M., Hsu, R., Barker, A.J., Gertz, C.C., Blelloch, R., and Ullian, E.M. (2011). Monoallelic deletion of the microRNA biogenesis gene Dgcr8 produces

deficits in the development of excitatory synaptic transmission in the prefrontal cortex. Neural Dev. *6*, 11.

Sellier, C., Freyermuth, F., Tabet, R., Tran, T., He, F., Ruffenach, F., Alunni, V., Moine, H., Thibault, C., Page, A., et al. (2013). Sequestration of DROSHA and DGCR8 by expanded CGG RNA repeats alters microRNA processing in fragile X-associated tremor/ataxia syndrome. Cell Rep *3*, 869–880.

Senturia, R., Laganowsky, A., Barr, I., Scheidemantle, B.D., and Guo, F. (2012). Dimerization and heme binding are conserved in amphibian and starfish homologues of the microRNA processing protein DGCR8. PLoS ONE *7*, e39688.

Serber, Z., and Ferrell, J.E., Jr. (2007). Tuning bulk electrostatics to regulate protein function. Cell *128*, 441–444.

Shiohama, A., Sasaki, T., Noda, S., Minoshima, S., and Shimizu, N. (2007). Nucleolar localization of DGCR8 and identification of eleven DGCR8-associated proteins. Exp. Cell Res. *313*, 4196–4207.

Sohn, S.Y., Bae, W.J., Kim, J.J., Yeom, K.H., Kim, V.N., and Cho, Y. (2007). Crystal structure of human DGCR8 core. Nat. Struct. Mol. Biol. *14*, 847–853.

Stark, K.L., Xu, B., Bagchi, A., Lai, W.S., Liu, H., Hsu, R., Wan, X., Pavlidis, P., Mills, A.A., Karayiorgou, M., and Gogos, J.A. (2008). Altered brain microRNA biogenesis contributes to phenotypic deficits in a 22q11-deletion mouse model. Nat. Genet. *40*, 751–760.

Steiner, D.F., Thomas, M.F., Hu, J.K., Yang, Z., Babiarz, J.E., Allen, C.D., Matloubian, M., Blelloch, R., and Ansel, K.M. (2011). MicroRNA-29 regulates T-box transcription factors and interferon-γ production in helper T cells. Immunity *35*, 169–181.

Strickfaden, S.C., Winters, M.J., Ben-Ari, G., Lamson, R.E., Tyers, M., and Pryciak, P.M. (2007). A mechanism for cell-cycle regulation of MAP kinase signaling in a yeast differentiation pathway. Cell *128*, 519–531.

Tang, X., Zhang, Y., Tucker, L., and Ramratnam, B. (2010). Phosphorylation of the RNase III enzyme Drosha at Serine300 or Serine302 is required for its nuclear localization. Nucleic Acids Res. *38*, 6610–6619.

Tang, X., Li, M., Tucker, L., and Ramratnam, B. (2011). Glycogen synthase kinase 3 beta (GSK3β) phosphorylates the RNAase III enzyme Drosha at S300 and S302. PLoS ONE *6*, e20391.

Tang, X., Wen, S., Zheng, D., Tucker, L., Cao, L., Pantazatos, D., Moss, S.F., and Ramratnam, B. (2013). Acetylation of drosha on the N-terminus inhibits its degradation by ubiquitination. PLoS ONE *8*, e72503.

Tözsér, J., Bagossi, P., Zahuczky, G., Specht, S.I., Majerova, E., and Copeland, T.D. (2003). Effect of caspase cleavage-site phosphorylation on proteolysis. Biochem. J. *372*, 137–143.

Triboulet, R., Chang, H.M., Lapierre, R.J., and Gregory, R.I. (2009). Post-transcriptional control of DGCR8 expression by the Microprocessor. RNA *15*, 1005–1011.

Wada, T., Kikuchi, J., and Furukawa, Y. (2012). Histone deacetylase 1 enhances microRNA processing via deacetylation of DGCR8. EMBO Rep. *13*, 142–149.

Wang, Y., Medvid, R., Melton, C., Jaenisch, R., and Blelloch, R. (2007). DGCR8 is essential for microRNA biogenesis and silencing of embryonic stem cell self-renewal. Nat. Genet. *39*, 380–385.

Wostenberg, C., Quarles, K.A., and Showalter, S.A. (2010). Dynamic origins of differential RNA binding function in two dsRBDs from the miRNA “microprocessor” complex. Biochemistry *49*, 10728–10736.

Wu, J., Qian, J., Li, C., Kwok, L., Cheng, F., Liu, P., Perdomo, C., Kotton, D., Vaziri, C., Anderlind, C., et al. (2010). miR-129 regulates cell proliferation by downregulating Cdk6 expression. Cell Cycle *9*, 1809–1818.

Yeom, K.H., Lee, Y., Han, J., Suh, M.R., and Kim, V.N. (2006). Characterization of DGCR8/Pasha, the essential cofactor for Drosha in primary miRNA processing. Nucleic Acids Res. *34*, 4622–4629.

Yi, R., Doehle, B.P., Qin, Y., Macara, I.G., and Cullen, B.R. (2005). Overexpression of exportin 5 enhances RNA interference mediated by short hairpin RNAs and microRNAs. RNA *11*, 220–226.

Yi, R., Pasolli, H.A., Landthaler, M., Hafner, M., Ojo, T., Sheridan, R., Sander, C., O’Carroll, D., Stoffel, M., Tuschl, T., and Fuchs, E. (2009). DGCR8-dependent microRNA biogenesis is essential for skin development. Proc. Natl. Acad. Sci. USA *106*, 498–502.

Zheng, C., Xiang, J., Hunter, T., and Lin, A. (1999). The JNKK2-JNK1 fusion protein acts as a constitutively active c-Jun kinase that stimulates c-Jun transcription activity. J. Biol. Chem. *274*, 28966–28971.

Phosphorylation of DGCR8 Increases Its Intracellular Stability and Induces a Pro-growth miRNA Profile

Kristina M. Herbert,[1] Genaro Pimienta,[1] Suzanne J. DeGregorio,[1] Andrei Alexandrov,[1] and Joan A. Steitz[1,*]

[1] Department of Molecular Biophysics and Biochemistry, Howard Hughes Medical Institute, Yale University School of Medicine, New Haven, CT 06536, USA

*Correspondence: joan.steitz@yale.edu

SUPPLEMENTAL INFORMATION:

Figures S1- S5

Tables S1, S2, and S3

Captions for Tables S1-S6 and Data S1

Supplemental Experimental Procedures

Supplemental References

INVENTORY OF SUPPLEMENTAL INFORMATION

Figures:

Figure S1. Sequence Coverage; Related to Figure 1C. This figure displays the DGCR8 amino acid sequences that were covered in our mass spectrometry data.

Figure S2. DGCR8 Is Targeted by MAPKs; Related to Figure 2. This figure shows the identified MAPK docking motifs in DGCR8 (A), in vitro kinase assays for JNK /MAPK (B), confirmation that we were able to isolate activated ERK (C), and p-JNK immunoblot showing that JNK was not activated upon serum addition to HeLa cells.

Figure S3. DGCR8 Decay Is Responsible for Differences in DGCR8 Levels; Related to Figure 3. This figure shows decay data for DGCR8 mutants in HeLa strain 1.

Figure S4. Microprocessor Complexes Containing Phosphomimetic DGCR8 Do Not Exhibit Altered Specific Pri-miRNA Processing Activity *In Vitro*; Related to Figure 5. This figure shows in vitro processing assays for MC isolated from HEK 293T cells for several pri-miRNAs.

Figure S5. MiRNA Expression Profiles; Related to Figure 5. This figure presents controls for the RNA-seq data presented in Figure 5.

Tables:

Table S1. Phosphosites Mapped with Poor Statistics in DGCR8 Expressed in HEK293 Cells; Related to Table 1. This table presents data on additional phosphosites that were mapped but were not deemed statistically significant.

Table S2. Mutant and Mimetic DGCR8 Constructs; Related to Figure 1F. This table presents the sequences of all of the mutant DGCR8 constructs created for this manuscript.

Table S3. Potential Kinases; Related to Figure 2. This table presents kinases predicted to phosphorylate each of the identified phosphosites.

Table S4. 75 MiRNAs that Show a Greater than 2-fold Up-Regulation in the Mim23-Expressing Cells Relative to Both WT- and Mut23-Expressing Cells; Related to Figure 5.

Table S5. 7 MiRNAs that Show a Greater than 2-fold Up-Regulation in the Mim23-Expressing Cells Relative to Both WT- and Mut23-Expressing Cells; Related to Figure 5.

Table S6. 534 MiRNAs that Show Less than a 2-fold Change in the Mim23-Expressing Cells Relative to Either WT- and/or Mut23-Expressing Cells; Related to Figure 5.

Data Files:

Data S1. Spectral Evidence for All DGCR8 Phosphosites Identified; Related to Figure 1D.

Additional Information:

Supplemental Experimental Procedures

Supplemental References

SUPPLEMENTAL INFORMATION

Figure S1. Sequence Coverage; Related to Figure 1C. The DGCR8 amino acid sequence is shown in gray with the peptide sequences that were identified in each mass-spectrometry experiment shown in bold. Identified phosphosites from each experiment are highlighted in green.

Insect Cell Samples

TiO_2 Elute Sequence coverage = 49.2 %

```
        10         20         30         40         50         60         70         80         90        100
METDESPSPL PCGPAGEAVM ESRARPFQAL PREQSPPPPL QTSSGAEVMD VGSGGDGQSE LPAEDPFNFY GASLLSKGSF SKGRLLIDPN CSGHSPRTAR
HAPAVRKFSP DLKLLKDVKI SVSFTESCRS KDRKVLYTGA ERDVRAECGL LLSPVSGDVH ACPFGGSVGD GVGIGGESAD KKDEENELDQ EKRVEYAVLD
ELEDFTDNLE LDEEGAGGFT AKAIVQRDRV DEEALNFPYE DDFDNDVDAL LEEGLCAPKK RRTEEKYGGD SDHPSDGETS VQPMMTKIKT VLKSRGRPPT
EPLPDGWIMT FHNSGVPVYL HRESRVVTWS RPYFLGTGSI RKHDPPLSSI PCLHYKKMKD NEEREQSSDL TPSGDVSPVK PLSRSAELEF PLDEPDSMGA
DPGPPDEKDP LGAEAAPGAL GQVKAKVEVC KDESVDLEEF RSYLEKRFDF EQVTVKKFRT WAERRQFNRE MKRKQAESER PILPANQKLI TLSVQDAPTK
KEFVINPNGK SEVCILHEYM QRVLKVRPVY NFFECENPSE PFGASVTIDG VTYGSGTASS KKLAKNKAAR ATLEILIPDF VKQTSEEKPK DSEELEYFNH
ISIEDSRVYE LTSKAGLLSP YQILHECLKR NHGMGDTSIK FEVVPGKNQK SEYVMACGKH TVRGWCKNKR VGKQLASQKI LQLLHPHVKN WGSLLRMYGR
ESSKMVKQET SDKSVIELQQ YAKKNKPNLH ILSKLQEEMK RLAEEREETR KKPKMSIVAS AQPGGEPLCT VDV
```

IMAC Elute Sequence coverage = 18.1 %

```
        10         20         30         40         50         60         70         80         90        100
METDESPSPL PCGPAGEAVM ESRARPFQAL PREQSPPPPL QTSSGAEVMD VGSGGDGQSE LPAEDPFNFY GASLLSKGSF SKGRLLIDPN CSGHSPRTAR
HAPAVRKFSP DLKLLKDVKI SVSFTESCRS KDRKVLYTGA ERDVRAECGL LLSPVSGDVH ACPFGGSVGD GVGIGGESAD KKDEENELDQ EKRVEYAVLD
ELEDFTDNLE LDEEGAGGFT AKAIVQRDRV DEEALNFPYE DDFDNDVDAL LEEGLCAPKK RRTEEKYGGD SDHPSDGETS VQPMMTKIKT VLKSRGRPPT
EPLPDGWIMT FHNSGVPVYL HRESRVVTWS RPYFLGTGSI RKHDPPLSSI PCLHYKKMKD NEEREQSSDL TPSGDVSPVK PLSRSAELEF PLDEPDSMGA
DPGPPDEKDP LGAEAAPGAL GQVKAKVEVC KDESVDLEEF RSYLEKRFDF EQVTVKKFRT WAERRQFNRE MKRKQAESER PILPANQKLI TLSVQDAPTK
KEFVINPNGK SEVCILHEYM QRVLKVRPVY NFFECENPSE PFGASVTIDG VTYGSGTASS KKLAKNKAAR ATLEILIPDF VKQTSEEKPK DSEELEYFNH
ISIEDSRVYE LTSKAGLLSP YQILHECLKR NHGMGDTSIK FEVVPGKNQK SEYVMACGKH TVRGWCKNKR VGKQLASQKI LQLLHPHVKN WGSLLRMYGR
ESSKMVKQET SDKSVIELQQ YAKKNKPNLH ILSKLQEEMK RLAEEREETR KKPKMSIVAS AQPGGEPLCT VDV
```

IMAC FT Sequence coverage = 67.0 %

```
        10         20         30         40         50         60         70         80         90        100
METDESPSPL PCGPAGEAVM ESRARPFQAL PREQSPPPPL QTSSGAEVMD VGSGGDGQSE LPAEDPFNFY GASLLSKGSF SKGRLLIDPN CSGHSPRTAR
HAPAVRKFSP DLKLLKDVKI SVSFTESCRS KDRKVLYTGA ERDVRAECGL LLSPVSGDVH ACPFGGSVGD GVGIGGESAD KKDEENELDQ EKRVEYAVLD
ELEDFTDNLE LDEEGAGGFT AKAIVQRDRV DEEALNFPYE DDFDNDVDAL LEEGLCAPKK RRTEEKYGGD SDHPSDGETS VQPMMTKIKT VLKSRGRPPT
EPLPDGWIMT FHNSGVPVYL HRESRVVTWS RPYFLGTGSI RKHDPPLSSI PCLHYKKMKD NEEREQSSDL TPSGDVSPVK PLSRSAELEF PLDEPDSMGA
DPGPPDEKDP LGAEAAPGAL GQVKAKVEVC KDESVDLEEF RSYLEKRFDF EQVTVKKFRT WAERRQFNRE MKRKQAESER PILPANQKLI TLSVQDAPTK
KEFVINPNGK SEVCILHEYM QRVLKVRPVY NFFECENPSE PFGASVTIDG VTYGSGTASS KKLAKNKAAR ATLEILIPDF VKQTSEEKPK DSEELEYFNH
ISIEDSRVYE LTSKAGLLSP YQILHECLKR NHGMGDTSIK FEVVPGKNQK SEYVMACGKH TVRGWCKNKR VGKQLASQKI LQLLHPHVKN WGSLLRMYGR
ESSKMVKQET SDKSVIELQQ YAKKNKPNLH ILSKLQEEMK RLAEEREETR KKPKMSIVAS AQPGGEPLCT VDV
```

Total Sequence coverage = 73.5 %

```
        10         20         30         40         50         60         70         80         90        100
METDESPSPL PCGPAGEAVM ESRARPFQAL PREQSPPPPL QTSSGAEVMD VGSGGDGQSE LPAEDPFNFY GASLLSKGSF SKGRLLIDPN CSGHSPRTAR
HAPAVRKFSP DLKLLKDVKI SVSFTESCRS KDRKVLYTGA ERDVRAECGL LLSPVSGDVH ACPFGGSVGD GVGIGGESAD KKDEENELDQ EKRVEYAVLD
ELEDFTDNLE LDEEGAGGFT AKAIVQRDRV DEEALNFPYE DDFDNDVDAL LEEGLCAPKK RRTEEKYGGD SDHPSDGETS VQPMMTKIKT VLKSRGRPPT
EPLPDGWIMT FHNSGVPVYL HRESRVVTWS RPYFLGTGSI RKHDPPLSSI PCLHYKKMKD NEEREQSSDL TPSGDVSPVK PLSRSAELEF PLDEPDSMGA
DPGPPDEKDP LGAEAAPGAL GQVKAKVEVC KDESVDLEEF RSYLEKRFDF EQVTVKKFRT WAERRQFNRE MKRKQAESER PILPANQKLI TLSVQDAPTK
KEFVINPNGK SEVCILHEYM QRVLKVRPVY NFFECENPSE PFGASVTIDG VTYGSGTASS KKLAKNKAAR ATLEILIPDF VKQTSEEKPK DSEELEYFNH
ISIEDSRVYE LTSKAGLLSP YQILHECLKR NHGMGDTSIK FEVVPGKNQK SEYVMACGKH TVRGWCKNKR VGKQLASQKI LQLLHPHVKN WGSLLRMYGR
ESSKMVKQET SDKSVIELQQ YAKKNKPNLH ILSKLQEEMK RLAEEREETR KKPKMSIVAS AQPGGEPLCT VDV
```

Mammalian Cell Samples

Experiment 1 Sequence coverage = 52.9 %

```
        10         20         30         40         50         60         70         80         90        100
METDESPSPL PCGPAGEAVM ESRARPFQAL PREQSPPPPL QTSSGAEVMD VGSGGDGQSE LPAEDPFNFY GASLLSKGSF SKGRLLIDPN CSGHSPRTAR
HAPAVRKFSP DLKLLKDVKI SVSFTESCRS KDRKVLYTGA ERDVRAECGL LLSPVSGDVH ACPFGGSVGD GVGIGGESAD KKDEENELDQ EKRVEYAVLD
ELEDFTDNLE LDEEGAGGFT AKAIVQRDRV DEEALNFPYE DDFDNDVDAL LEEGLCAPKK RRTEEKYGGD SDHPSDGETS VQPMMTKIKT VLKSRGRPPT
EPLPDGWIMT FHNSGVPVYL HRESRVVTWS RPYFLGTGSI RKHDPPLSSI PCLHYKKMKD NEEREQSSDL TPSGDVSPVK PLSRSAELEF PLDEPDSMGA
DPGPPDEKDP LGAEAAPGAL GQVKAKVEVC KDESVDLEEF RSYLEKRFDF EQVTVKKFRT WAERRQFNRE MKRKQAESER PILPANQKLI TLSVQDAPTK
KEFVINPNGK SEVCILHEYM QRVLKVRPVY NFFECENPSE PFGASVTIDG VTYGSGTASS KKLAKNKAAR ATLEILIPDF VKQTSEEKPK DSEELEYFNH
ISIEDSRVYE LTSKAGLLSP YQILHECLKR NHGMGDTSIK FEVVPGKNQK SEYVMACGKH TVRGWCKNKR VGKQLASQKI LQLLHPHVKN WGSLLRMYGR
ESSKMVKQET SDKSVIELQQ YAKKNKPNLH ILSKLQEEMK RLAEEREETR KKPKMSIVAS AQPGGEPLCT VDV
```

Experiment 2 Sequence coverage = 60.0 %

```
        10         20         30         40         50         60         70         80         90        100
METDESPSPL PCGPAGEAVM ESRARPFQAL PREQSPPPPL QTSSGAEVMD VGSGGDGQSE LPAEDPFNFY GASLLSKGSF SKGRLLIDPN CSGHSPRTAR
HAPAVRKFSP DLKLLKDVKI SVSFTESCRS KDRKVLYTGA ERDVRAECGL LLSPVSGDVH ACPFGGSVGD GVGIGGESAD KKDEENELDQ EKRVEYAVLD
ELEDFTDNLE LDEEGAGGFT AKAIVQRDRV DEEALNFPYE DDFDNDVDAL LEEGLCAPKK RRTEEKYGGD SDHPSDGETS VQPMMTKIKT VLKSRGRPPT
EPLPDGWIMT FHNSGVPVYL HRESRVVTWS RPYFLGTGSI RKHDPPLSSI PCLHYKKMKD NEEREQSSDL TPSGDVSPVK PLSRSAELEF PLDEPDSMGA
DPGPPDEKDP LGAEAAPGAL GQVKAKVEVC KDESVDLEEF RSYLEKRFDF EQVTVKKFRT WAERRQFNRE MKRKQAESER PILPANQKLI TLSVQDAPTK
KEFVINPNGK SEVCILHEYM QRVLKVRPVY NFFECENPSE PFGASVTIDG VTYGSGTASS KKLAKNKAAR ATLEILIPDF VKQTSEEKPK DSEELEYFNH
ISIEDSRVYE LTSKAGLLSP YQILHECLKR NHGMGDTSIK FEVVPGKNQK SEYVMACGKH TVRGWCKNKR VGKQLASQKI LQLLHPHVKN WGSLLRMYGR
ESSKMVKQET SDKSVIELQQ YAKKNKPNLH ILSKLQEEMK RLAEEREETR KKPKMSIVAS AQPGGEPLCT VDV
```

Table S1. Phosphosites Mapped with Poor Statistics in DGCR8 Expressed in HEK293 Cells; Related to Table 1. The amino acid number and residue of each mapped phosphosite is given. The posterior error probability (PEP), MaxQuant score, as well as the number of phosphopeptides identified in each experimental replicate (Experiment 1 or 2) or condition (with or without calyculin A treatment) are shown.

	**HEK293 cells Experiment 1**					**HEK293 cells Experiment 2**				
	Coverage 52.9%		Number of peptides phosphorylated			Coverage 60.0%		Number of peptides phosphorylated		
aa sites	PEP	score	total	no treat-ment	calyculin	PEP	score	total	no treat-ment	calyculin
S127	NA	NA	0/0	0	0	6.38E-02	39.39	1/37	0	1
T138	1.47E+02	28.869	1/44	0	1	NA	NA	0/28	0	0
T454	5.16E-01	9.1985	1/54	0	1	NA	NA	0/74	0	0
S478	3.55E-01	25.94	1/39	0	1	NA	NA	0/33	0	0
T710	NA	NA	0/15	0	0	2.62E-01	22.777	1/2	0	1
S714	9.56E-02	58.918	3/59	0	3	NA	NA	0/19	0	0

Data S1. Spectral Evidence for All DGCR8 Phosphosites Identified; Related to Figure 1D. See supplemental pdf file.

Shown are representative fragmentation spectra of phosphopeptides for each of the 23 phosphosites investigated in this work (Figure 1E), as well as those for which the statistical significance was not as great (Supplementary Table 1). Labels indicate the phosphosite identified in each fragmentation pattern. Spectra are shown as displayed by the MaxQuant-Andromeda output. The header in each spectrum includes the different ProteinKnowledgeBase (UniProt-KB) entries of the protein identified (Protein), which in this case is always DGCR8. Next, the identification score is shown (Score), followed by the raw data file from which the shown spectrum was retrieved (Source), Scan number (Scannumber), and the instrument setting (Method). In our experiments, the Method corresponds to ion trap (IT) mass spectrometry (MS) followed by collision-induced dissociation (CID) tandem mass spectrometry (MS2; or 2). The "b" and "y" fragmentation ions are indicated in blue and red, respectively, along the peptide sequence assigned to the spectrum and in the fragmentation spectrum itself. Phosphorylated peptides have a superscript "ph" tag. The other modification shown is "ox", referring to methionine oxidation. The meaning of superscript labels in the spectra is as follows: -H20 (water loss); -NH3 (ammonium loss); * (oxidation); and 2+ or 3+ (charge). A large fraction of the phosphopeptides identified has more than 1 phosphorylated residue and/or are longer than 20 amino acids. MaxQuant-Andromeda, the search engine used in our studies, was developed to identify peptides with such characteristics (Cox et al., 2011).

Table S2. Mutant and Mimetic DGCR8 Constructs; Related to Figure 1F. DNA sequences and amino acids (aa) of the WT, Mutant (Mut23 and Mut14), and Mimetic (Mim23 and Mim11) DGCR8 constructs are provided for each identified phosphosite. • indicates that a specific aa residue is mutated to either prevent or mimic phosphorylation, while an empty cell/box indicates that the sequence is WT. Mutation sites for Mim11 and Mut14 were selected to be scattered uniformly along the protein primary sequence.

	WT		Mutant				Mimetic			
aa sites	DNA seq	aa	DNA seq	aa	Mut23	Mut14	DNA seq	aa	Mim23	Mim11
35	TCT	Ser	GTT	Val	•	•	GAT	Asp	•	•
42	ACG	Thr	GCG	Ala	•	•	GAC	Asp	•	
59	TCC	Ser	GTC	Val	•		GAC	Asp	•	
92	AGT	Ser	GAT	Val	•	•	GTT	Asp	•	
95	AGC	Ser	GTC	Val	•	•	GAC	Asp	•	•
109	TCC	Ser	GTC	Val	•	•	GAC	Asp	•	•
123	AGC	Ser	GTC	Val	•		GAC	Asp	•	
125	TGG	Thr	GCC	Ala	•		GAC	Asp	•	
153	AGC	Ser	GTC	Val	•	•	GAC	Asp	•	•
156	AGT	Ser	GAT	Val	•	•	GTT	Asp	•	•
267	TAT	Tyr	TTT	Phe	•		GAG	Glu	•	
271	AGC	Ser	GTC	Val	•		GAC	Asp	•	•
275	TCC	Ser	GTC	Val	•	•	GAC	Asp	•	•
279	ACA	Thr	GCA	Ala	•		GAC	Asp	•	•
280	TCA	Ser	GTT	Val	•		GAT	Asp	•	
371	ACC	Thr	GCC	Ala	•	•	GAC	Asp	•	
373	AGT	Ser	GAT	Val	•	•	GTT	Asp	•	•
377	TCC	Ser	GTC	Val	•	•	GAC	Asp	•	
383	AGC	Ser	GTC	Val	•	•	GAC	Asp	•	•
385	TCT	Ser	GTT	Val	•		GAT	Asp	•	•
397	TCT	Ser	GTT	Val	•	•	GAT	Asp	•	
434	TCC	Ser	GTC	Val	•	•	GAC	Asp	•	
493	TCA	Ser	GTA	Val	•		GAC	Asp	•	

Table S3. Potential Kinases; Related to Figure 2. Two programs that match input protein sequences with post-translational modification motifs were used to predict potential kinases for each identified phosphosite. PHOSIDA motif matcher (Gnad et al., 2011; Gnad et al., 2007) identifies a class of candidate kinases, while NetworKin beta (Linding et al., 2007) reveals the gene name of a putative kinase.

aa sites	PHOSIDA	NetworKin beta
35	CAMK2, PKD, CHK1/2, CHK1	CDK2, CDK3
42	no prediction	STK11, NEK2
59	no prediction	CSNK2A2,CK2A1
92	no prediction	PRKAA1, PRKAA2 NEK2,
95	CK1, CDK1	CDK2, CDK3
109	PKA, CAMK2	MAPK14, MAPK11, MAPK13, MAPK12, CAMK2G, CDK2, CDK3
123	CK2, GSK3	CSNK2A2,CK2A1
125	no prediction	TGFBR2, ACVR2B, PRKAA1, PRKAA2, ADRBK1, GRK1, GRK5, ADRBK2
153	NEK6	MAPK14, MAPK11, MAPK13, MAPK12, MAPK9, MAPK10, MAPK8, DMK, CDC42BPA
156	CK1	PRKAA1, PRKAA2, MOK, ICK, CSNK2A2,CK2A1
267	SRC	IGF1R, INSR, LYN, SRC, LCK, FYN, FGR, HCK, BLK, PTK6, YES1, MAP2K6, MAP2K4, MAP2K3
271	GSK3	CSNK2A2,CK2A1
275	CK1, CK2	CSNK2A2,CK2A1
279	no prediction	TGFBR2, ACVR2B, TLK1, PRKDC,
280	PLK1	TLK1
371	CK1	CDK2, CDK3
373	GSK3, NEK6	GSK3B, GSK3A
377	CK1, CDK2, CDK1	CDK2, CDK3, MAPK14, MAPK11, MAPK13, MAPK12, MAPK9, MAPK10, MAPK8,
383	no prediction	MAPKAPK5, MAPKAPK2, MAPKAPK3, MAP2K6, MAP2K4, MAP2K3, DMK, CDC42BPA, MOK, ICK, PAK2, PAK3, CAMK2G, CLK1, CLK2
385	NEK6	DMK, CDC42BPA
397	no prediction	CSNK2A2,CK2A1
434	PKD, PLK1, CHK1	CSNK2A2,CK2A1
493	no prediction	DMK, CDC42BPA

Figure S2. DGCR8 Is Targeted by MAPKs; Related to Figure 2. (A) MAPKs bind characteristic primary amino acid sequences within target proteins, known as D-sites (Garai et al., 2012). Shown are sequences within DGCR8 that match this D-site motif, where X and ϕ correspond to any amino acid and a hydrophobic amino acid, respectively. (B) FLAG-JNK1a1 was immunoprecipitated from HEK293T cells that had been transfected with WT or the APF mutant FLAG-JNK1a1, or FLAG-MKK7B2 fusions of WT or the APF mutant JNK1a1. Immunoblots of these immunoprecipitates were probed with anti-FLAG to confirm protein presence and anti-pJNK to confirm activation of the constitutively activated construct. (C) HA-ERK was immunoprecipitated from HEK293T cells that had been transfected with either GFP alone, as a negative control (-), or HA-ERK together with MAPKK1-K97M, MAPKK1-R4F, or GFP. Immunoblots of these immunoprecipitates were probed with anti-HA to confirm protein presence and anti-pERK to confirm activation of ERK in the presence of its constitutively activated upstream kinase. (D) Strain 1 HeLa Flp-In cells stably expressing WT-F-DGCR8 or an empty vector were serum starved overnight, then treated for 2 hr with either DMSO control, U0126 (MEK1/2 inhibitor), or SP600125 (JNK inhibitor). Cells were then metabolically labeled with $^{32}PO_4$ upon serum addition for 4 hr. Immunoblots of total cell lysates (input) were probed for p-JNK activity.

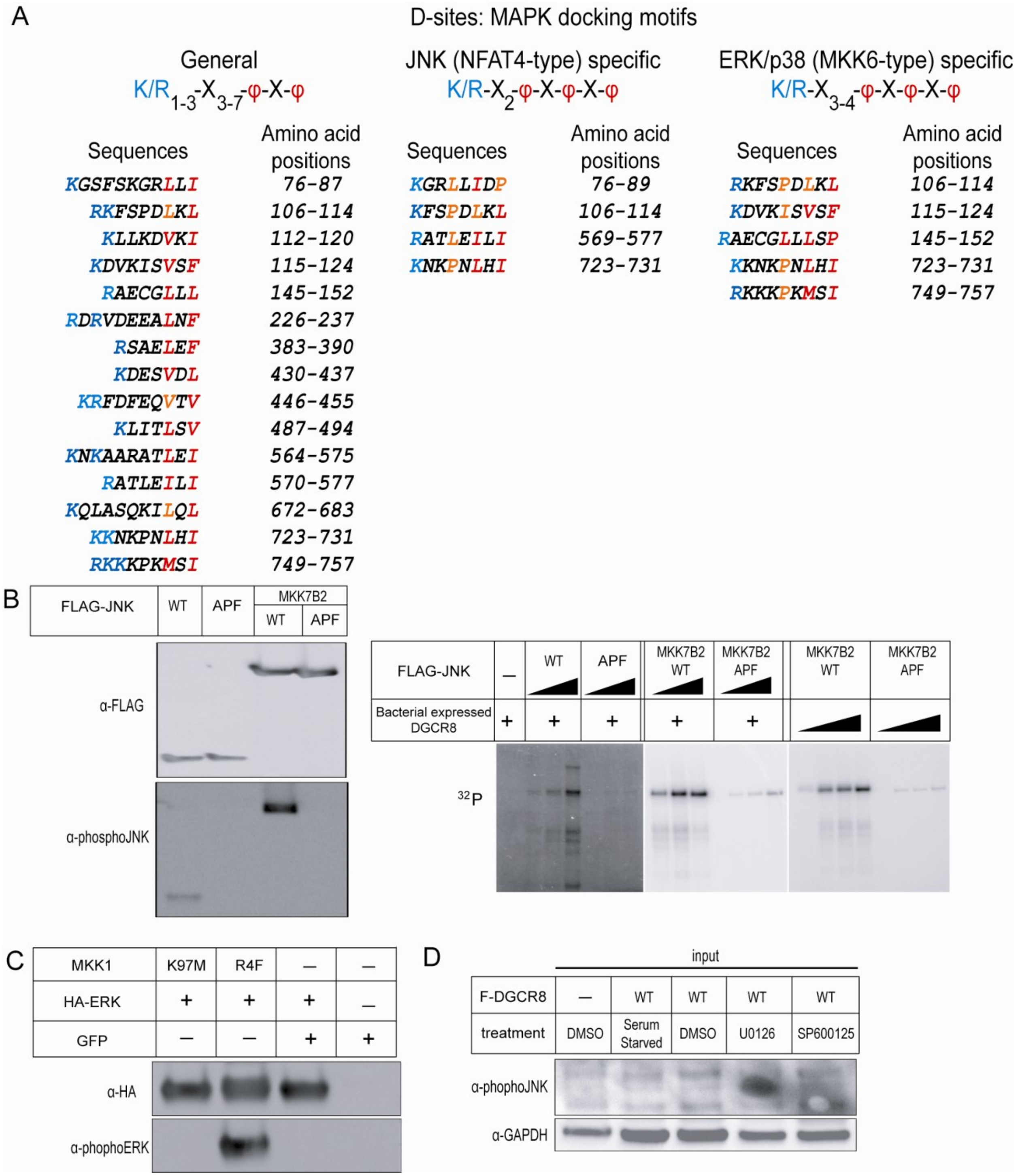
A
D-sites: MAPK docking motifs
General
K/R1-3-X3-7-φ-X-φ
Sequences
Amino acid positions
KGSFSKGRLLI 76-87
RKFSPDLKL 106-114
KLLKDVKI 112-120
KDVKISVSF 115-124
RAECGLLL 145-152
RDRVDEEALNF 226-237
RSAELEF 383-390
KDESVDL 430-437
KRFDFEQVTV 446-455
KLITLSV 487-494
KNKAARATLEI 564-575
RATLEILI 570-577
KQLASQKILQL 672-683
KKNKPNLHI 723-731
RKKKPKMSI 749-757
JNK (NFAT4-type) specific
K/R-X2-φ-X-φ-X-φ
Sequences
Amino acid positions
KGRLLIDP 76-89
KFSPDLKL 106-114
RATLEILI 569-577
KNKPNLHI 723-731
ERK/p38 (MKK6-type) specific
K/R-X3-4-φ-X-φ-X-φ
Sequences
Amino acid positions
RKFSPDLKL 106-114
KDVKISVSF 115-124
RAECGLLLSP 145-152
KKNKPNLHI 723-731
RKKKPKMSI 749-757
B
FLAG-JNK
WT
APF
MKK7B2
WT
APF
α-FLAG
α-phosphoJNK
FLAG-JNK
—
WT
APF
MKK7B2 WT
MKK7B2 APF
MKK7B2 WT
MKK7B2 APF
Bacterial expressed DGCR8
+
32P
C
MKK1
K97M
R4F
—
HA-ERK
+
GFP
α-HA
α-phophoERK
D
input
F-DGCR8
—
WT
treatment
DMSO
Serum Starved
DMSO
U0126
SP600125
α-phophoJNK
α-GAPDH

Figure S3. DGCR8 Decay Is Responsible for Differences in DGCR8 Levels; Related to Figure 3. Strain 1 isogenic HeLa Flp-In cells stably expressing WT-, Mut23-, or Mim23-F-DGCR8 were treated with 100 μg/mL cycloheximide. Cells were harvested at 0, 4, and 16 hr. Immunoblots were performed on total cell lysates to monitor DGCR8 decay.

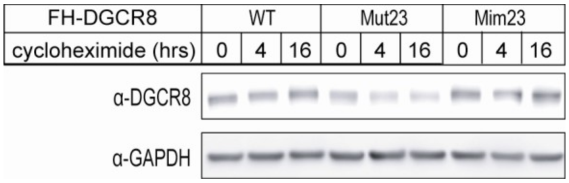

Figure S4. Microprocessor Complexes Containing Phosphomimetic DGCR8 Do Not Exhibit Altered Specific Pri-miRNA Processing Activity *In Vitro*; Related to Figure 5. (A) *In vitro* pri-miRNA-processing assays were performed by incubating various body-labeled pri-miRNAs and a short (35 nt) stable RNA, which functioned as a loading control (LC), with immunoprecipitated MCs from HEK293T cells that had been transiently transfected with GFP, Myc-Drosha, and either an empty vector or a vector expressing WT-, Mut23-, or Mim23-FH-DGCR8. The input RNAs are shown in the lanes labeled RNA. * mark the pre-miR species. Contrast has been adjusted separately on the ladder lanes. (B) Immunoblots of MCs isolated via anti-FLAG immunoprecipitation of lysates of HEK293T cells transiently transfected with vectors expressing GFP, Myc-Drosha, and either an empty vector, or a vector expressing WT-, Mut23-, or Mim23-FH-DGCR8. These MC immunoprecipitates were used for the processing assays shown in the top panels of A. (C) HEK293T cells were transiently transfected with vectors expressing GFP, Myc-Drosha, and either an empty vector, or a vector expressing WT-, Mut23-, Mim23-FH-DGCR8, or WT-SNAP-DGCR8. Immunoblots of anti-FLAG immunoprecipitated MCs were probed for p68 and p72 helicases as well as for GAPDH.

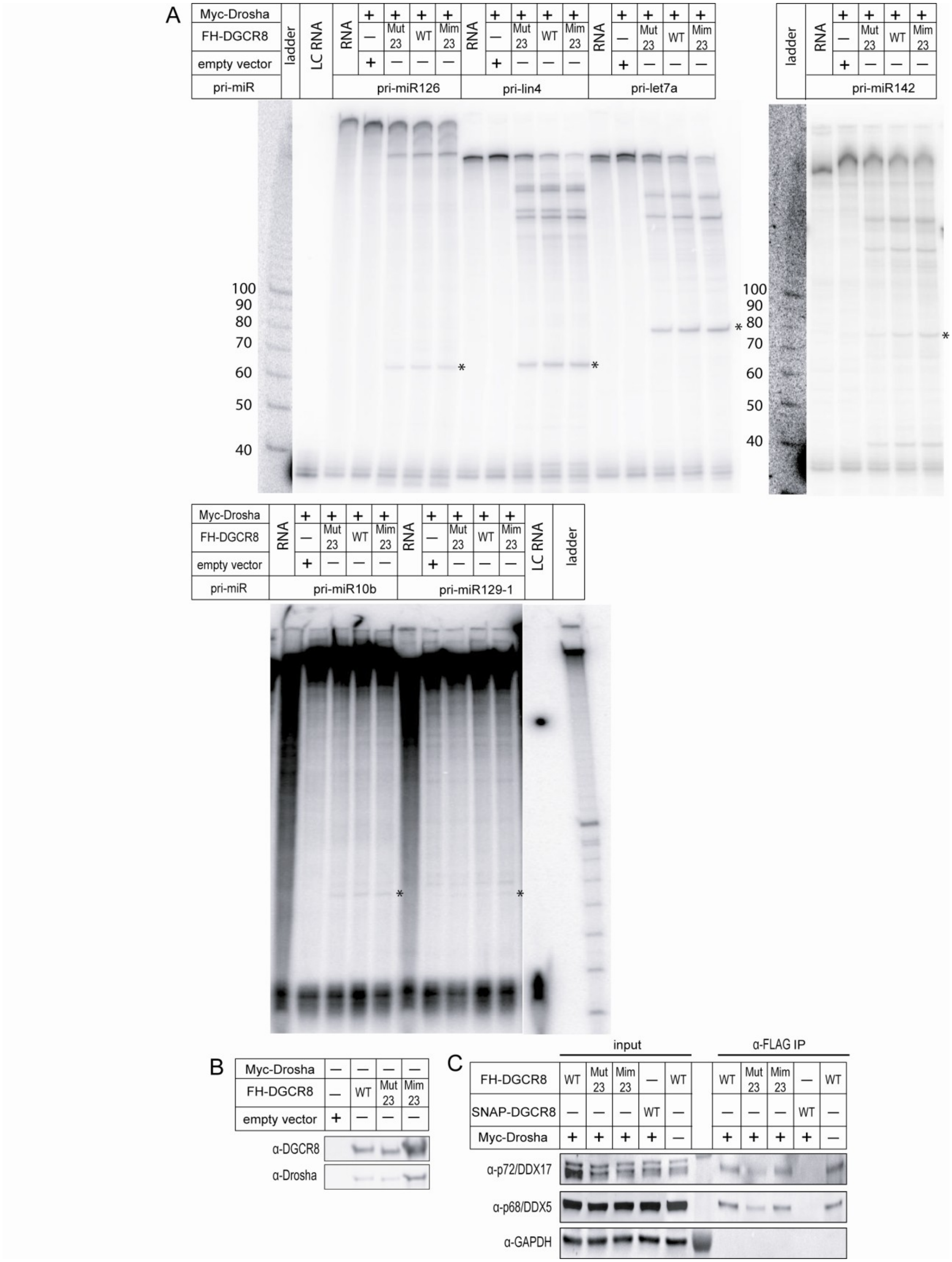
A
Myc-Drosha
FH-DGCR8
empty vector
pri-miR
ladder
LC RNA
RNA
Mut 23
WT
Mim 23
pri-miR126
pri-lin4
pri-let7a
pri-miR142
100
90
80
70
60
50
40
pri-miR10b
pri-miR129-1
B
α-DGCR8
α-Drosha
C
input
α-FLAG IP
SNAP-DGCR8
α-p72/DDX17
α-p68/DDX5
α-GAPDH

Figure S5. MiRNA Expression Profiles; Related to Figure 5. (A) Next-generation sequencing was used to profile levels of small RNAs from strain 2 isogenic HeLa Flp-In cells stably expressing Mim23-, WT-, or Mut23-F-DGCR8. Each dot represents, for an individual mature miRNA, the $\log_2$ relative expression in Mim23- over Mut23-F-DGCR8 cells versus the $\log_2$ relative expression in Mim23- over WT-F-DGCR8 cells. Dotted lines are shown at 1 and -1, corresponding to a 2-fold change up or down, respectively; thus, a miRNA with a greater than 2-fold up or down change in the Mim23 sample relative to both the Mut23 and WT sample will be in the upper right or lower left quadrant. Red, blue, and green dots represent separate biological replicates, while black shows the average value for each miRNA from all three replicates. Error bars are omitted for simplicity but are given in Tables S4-6. (B) Plots showing positive correlations between biological replicates of the next-generation sequencing $\log_2$ relative expression data for each mature miRNA. (C) TaqMan miRNA quantitative PCR was performed to assess expression (mean ± STD) of miR-181a (n = 3), and miR-93 (n = 2), miR-129 (n=3), miR-10b (n=4) relative to U6 in strain 2 HeLa cells stably expressing Mim23-, Mut23-, or WT-F-DGCR8.

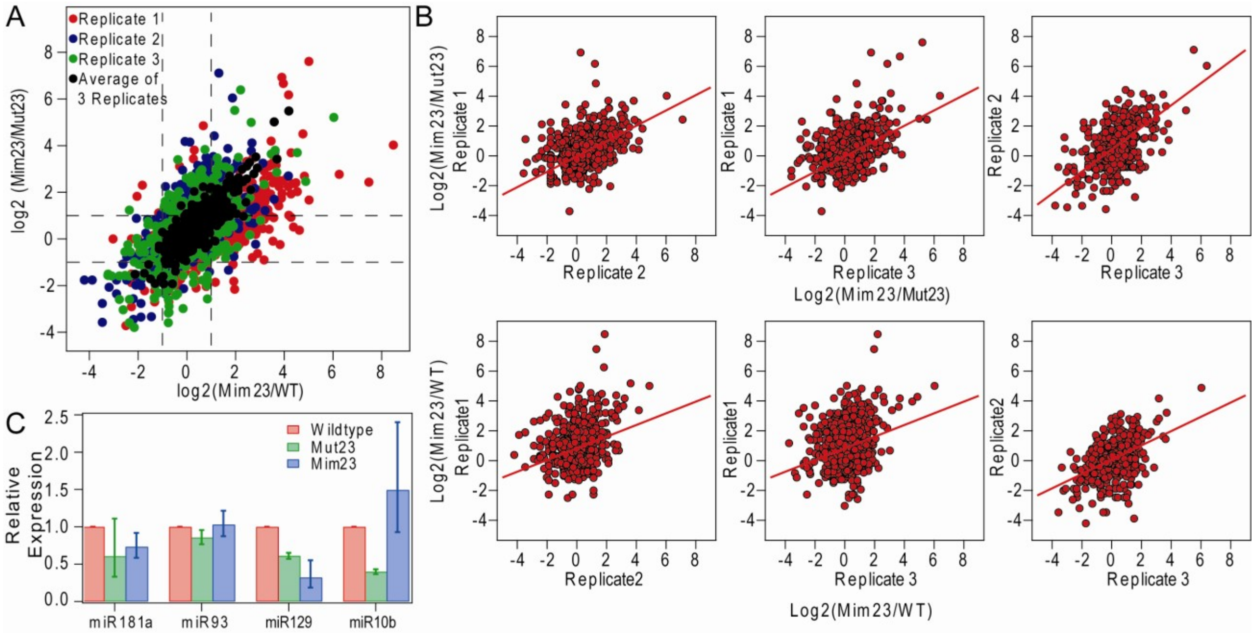

Table S4. 75 MiRNAs that Show a Greater than 2-fold Up-Regulation in the Mim23-Expressing Cells Relative to Both WT- and Mut23-Expressing Cells; Related to Figure 5. The table shows the miRNA id, the sum of reads from all samples analyzed that mapped to this miRNA, the precursor id corresponding to the mature miRNA, the average and standard error (n=3) of the $\log_2$ relative expression in Mim23- compared to Mut23-F-DGCR8-expressing cells, the average and standard error (n=3) of the $\log_2$ relative expression in Mim23- compared to WT-F-DGCR8-expressing cells.

Table S5. 7 MiRNAs that Show a Greater than 2-fold Up-Regulation in the Mim23-Expressing Cells Relative to Both WT- and Mut23-Expressing Cells; Related to Figure 5. The table shows the miRNA id, the sum of reads from all samples analyzed that mapped to this miRNA, the precursor id corresponding to the mature miRNA, the average and standard error (n=3) of the $\log_2$ relative expression in Mim23- compared to Mut23-F-DGCR8-expressing cells, the average and standard error (n=3) of the $\log_2$ relative expression in Mim23- compared to WT-F-DGCR8-expressing cells.

Table S6. 534 MiRNAs that Show Less than a 2-fold Change in the Mim23-Expressing Cells Relative to Either WT- and/or Mut23-Expressing Cells; Related to Figure 5. The table shows the miRNA id, the sum of reads from all samples analyzed that mapped to this miRNA, the precursor id corresponding to the mature miRNA, the average and standard error (n=3) of the $\log_2$ relative expression in Mim23- compared to Mut23-F-DGCR8- expressing cells, the average and standard error (n=3) of the $\log_2$ relative expression in Mim23- compared to WT-F-DGCR8-expressing cells.

SUPPLEMENTAL EXPERIMENTAL PROCEDURES

Plasmids

pFLAG/HA-DGCR8 (pFH-DGCR8) and pcDNA4/TO/cmycDrosha (Landthaler et al., 2004) were purchased (Addgene) and used to clone pCS3-MT-MycDrosha, all wildtype, mutant and mimetic FH-DGCR8 vectors, pSNAP-DGCR8 (all for transient transfections), pcDNA5/FRT-F-DGCR8 (for stable transfections), pET28a-DGCR8 (for bacterial expression), and pFast-Bac1-HisDGCR8 (for baculovirus expression). A Myc-Drosha PCR fragment was inserted into the XbaI site of the pCS3-MT plasmid to create pCS3-MT-MycDrosha. DGCR8 mutant and mimetic constructs were created with QuikChange multi- and single-site directed mutagenesis kits (Stratagene). To create pcDNA5/FRT-FLAG-DGCR8 plasmids, the HA tags in all versions of pFH-DGCR8 were deleted using site-directed mutagenesis and plasmids were digested with KpnI and NotI to obtain FLAG-DGCR8 inserts, which were subsequently ligated to the pCDNA5/FRT vector. DGCR8 PCR fragments were inserted into the NotI site of pSNAP-tag(m) vector (NEB) to create the pSNAP-DGCR8. pET28a-DGCR8 was cloned by inserting the DGCR8 PCR fragment between the EcoRI and HindIII sites of pET28a(+) (Novagen). His-tagged DGCR8 was excised from pET-28a-DGCR8 using the XbaI and HindIII sites and ligated into the same sites of pFast-Bac1 (Invitrogen) to give pFast-Bac1-HisDGCR8. pGFPmax was transfected for two reasons: (1) it allowed determination of transfection efficiency, and (2) it provided a loading control for the Northern blots. pcDNA3 was used as the empty vector control.

Mammalian Cell Assays

All mammalian cells (HEK293, HEK293T, HeLa) were cultured as in (Pawlicki and Steitz, 2008). Transfections were performed with TransIT-293 or HeLaMonster reagent (Mirus Bio) per manufacturer's instructions. Cells were harvested 30-48 hr later and frozen on dry ice for at least 30 min until further processing. Cells were lysed on ice for 45 min in lysis buffer [2% NP-40, 10% glycerol, 150 mM NaCl, 50 mM Tris-HCl pH 7.5, 5 mM EDTA, supplemented 1:100 with phosphatase inhibitor cocktails 1 and 2

(Sigma; Sigma later replaced cocktail 1 with 3) and Complete EDTA-free protease inhibitor tablets (Roche)] and spun 15 min at 15K g. Coomassie Plus (Bradford) Protein Assays (Thermo Scientific) were performed to ensure equal loading of lysates onto gels or onto resin for immunoprecipitation.

For metabolic labeling of cells, plates were washed 5 times with phosphate free DMEM (Gibco) and then incubated 3.5 hr in phosphate-free DMEM supplemented with $^{32}PO_4$ (PerkinElmer). Cells were treated with fresh 100 μg/mL cycloheximide (Sigma 100 mg/mL stock dissolved in EtOH), 20 μM U0126 (MEK1/2 inhibitor), 20 μM SP600125 (JNK inhibitor) (LC Labs), or 100 nM Calyculin A (Cell Signaling, LC labs, and Sigma) for ~20 min.

Stable HeLa Flp-In cell lines were created using a Flipase (Flp)/Flp recognition target site-directed recombination system (Invitrogen). A parent cell line was made by infecting HeLa(JW36) cells at low MOI (less than 5%) with a pTYF-based lentiviral construct containing (in succession): EGFP (expressed from EF-1α promoter and followed by the BGH polyadenylation site), the SV40 promoter upstream of an ATG-containing FRT site, and puromycin N-acetyl-transferase (expressed from the PGK promoter). Single EGFP-expressing colonies were selected for two weeks in media containing 0.2 μg/ml puromycin; this parent line yielded the strain 1 HeLa cells. The strain 2 parent line was made similarly, but did not express EGFP and was selected for Zeocin resistance. Isogenic cell lines expressing WT-, Mut23-, and Mim23-F-DGCR8 were produced from each parent Flp-In HeLa line by cotransfecting them with pcDNA5/FRT-FLAG-DGCR8 vectors and pOG44. Stable clones were selected using 200 μg/mL hygromycin (EMD-Millipore). For proliferation assays, strain 2 isogenic HeLa Flp-In cells stably expressing WT-, Mut23-, or Mim23-F-DGCR8 were plated at 200 cells per well in a 96-well plate. After settling overnight, cells were serum starved 24 hr and after serum addition cell proliferation was measured every 24 hr for 5 days using Cell Titer Glo reagent (Promega). Luminescence signals were recorded on a GloMax-Multi+ Plate reader (Promega). For *in vitro* scratch assays, strain 2 isogenic HeLa Flp-In cells stably expressing

WT-, Mut23-, or Mim23-F-DGCR8 were plated at 500,000 cells per 10-cm plate. After adhering overnight, cells were serum starved overnight and then a 200 μL pipette was used to create a scratch before the readdition of serum. Cells were photographed every 12 hr.

Immunochemistry

Equivalent amounts of each lysate were incubated for 2 hr at 4°C with pre-washed anti-FLAG M2 affinity resin (Sigma). Bound-resin was washed 4 times with wash buffer [10% glycerol, 250 mM NaCl, 50 mM Tris-HCl pH 7.5, and 2 mM EDTA supplemented 1:100 with phosphatase inhibitor cocktails 1 and 2 (Sigma) and Complete EDTA-free protease inhibitor tablets (Roche)]. Proteins for processing assays were eluted in wash buffer plus 50 μg/mL 3x FLAG peptide (Sigma). Immunoprecipitations for assessing co-purifying factors were washed with 500 mM NaCl wash buffer to reduce background and then diluted directly into SDS-PAGE loading buffer. Immunoprecipitates of metabolically-labeled cells were washed with 500 mM NaCl wash. Phosphatase treatment was performed by resuspending the washed MC-containing resin in 1 X λ phosphatase buffer, 1 mM $MnCl_2$, and 800 Units of λ Phosphatase (NEB). Samples were incubated at 30°C for 10 min and then diluted directly into SDS-PAGE loading buffer.

Immunoblots were performed according to (Pimienta et al., 2011) using 7.5–15 μg from each lysate. Blots were developed using ECL reagents (Thermo Scientific Femto Maximum Sensitivity Substrate or PerkinElmer Western Lightning ECL), images acquired on the Syngene G:Box Chemi XT^4 System, and quantitated using the GeneSys software. Immunofluorescence was performed according to (Pawlicki and Steitz, 2008) with primary antibodies diluted 1:100 and images acquired on a Leica TCS SP5 confocal microscope. The following antibodies were used: anti-DGCR8 (ProteinTech), anti-FLAG (Sigma F3165 for blots and F1804 for fluorescence), anti-Drosha, anti-GAPDH, anti-Dicer, anti-phospho-(Ser/Thr) MAPK/CDK substrate antibody sampler kit, MAPK antibody sampler kit, phospho-MAPK antibody sampler kit (Cell Signaling), anti-DDX5, anti-DDX17, anti-TRBP (Abcam), anti-Ago2 (Millipore), goat-anti-

mouse-Alexa488, goat-anti-rabbit-Alexa594 (Invitrogen), goat anti-mouse-HRP, and goat-anti-rabbit-HRP (Thermo Scientific).

Northern Blots

Northern blots were performed according to (Pawlicki and Steitz, 2008) using 7.5 μg RNA from each sample. Membranes were hybridized in ExpressHyb Solution (Clontech) overnight at 42°C to oligonucleotide probes end-labeled with T4 polynucleotide kinase in the presence of [γ-^{32}P]ATP. Probe sequences are shown below.

	DNA sequence (5′ to 3′)
GFP probe	CGTACTTCTCGATGCGGGTGTTGG
Myc probe	CAAGTCCTCTTCAGAAATGAGCTTTTGCTC
FLAG probe	CTTGTCATCGTCGTCCTTCTAGTCCAT

In Vitro Processing Assays

In vitro processing assays were performed according to (Pawlicki and Steitz, 2008) with MCs purified by anti-FLAG immunoprecipitation. Pri-miRNA encoding plasmids were cloned as in (Pawlicki and Steitz, 2008). Pri-miR16-2 *in vitro* transcription templates were generated by PCR on pcDNA3-pri-miR-15b~16-2 using a primer that adds a T7 RNA polymerase promoter. Other *in vitro* transcription templates were created by PCR with CMV and SP6 primers. *In vitro* transcription templates for generating the loading control were created by digesting pcDNA3 with BamHI and fill-in with T4 DNA polymerase. Approximately 1.25×10^5 cpm (~1 fmol) of *in vitro* transcribed pri-miRNA substrate and 1.5×10^4 cpm of the loading control were used per processing reaction. Reactions proceeded for 45 min at 37°C.

MiRNA Profiling

RNA from 10 cm plates of isogenic Flp-In HeLa cells expressing Mim23-, Mut23-, or WT-F-DGCR8 was prepared by Trizol (Invitrogen) extraction and subsequent Qiagen RNeasy cleanup procedure with on-column DNase treatment. RNA from 3 biological replicates was submitted to the Keck Yale Center for Genome Analysis for TruSeq small RNA library preparation and Illumina HiSeq 2000 sequencing. Data were analyzed using miRDeep2 (Friedlander et al., 2012). Reads were mapped to the human genome, Hg19. The quantifier module normalizes reads per mature miRNA to the total number of mature miRNA reads in each sample. For TaqMan quantitative PCR analysis of miRNA expression, cDNA was created and qPCRs were done using the TaqMan MicroRNA Reverse Transcription Kit, TaqMan Universal PCR Master Mix, No AmpErase UNG and individual miRNA assays [Applied Biosystems: hsa-miR-21 (ID: 000397), hsa-miR-181a (ID: 000480), hsa-let-7c (ID: 000379), hsa-miR-93 (ID: 000432), RNU6B (ID: 001093)] according to the manufacturer's instructions.

Search for MAPK Docking Motifs

A custom Perl script was written to search for instances of D-sites, as defined by (Garai et al., 2012), in a given protein amino acid sequence. The regular expressions [KR]{1,3}.{3,7}[ILVM].[ILVF] (General), [RK][P]..[LIV].[LIVMPF] (pepJIP type), [RK]..[LIVMP].[LIV].[LIVMPF] (pepNFAT4 type), [RK].{3,4}[LIVMP].[LIV].[LIVMPFA] (pepMKK6 type), [LI]..[RK][RK].{5}[LIVMP].[LIV].[LIVMPFA] (pepHePTP type), and [LIVMPFA].[LIV].{1,2}[LIVMP].{4,6}[LI]..[RK][RK] (RSK/MAPKAP type) were used.

In vitro Kinase Assays

HA-ERK2 (pcDNA3-HA-ERK2 WT) and MKK1 plasmids (pMCL-HA-MAPKK1-R4F [delta(31-51)/S218E/S222D] and pMCL-HA-MAPKK1-8E (K97M)) were the kind gift of B. Turk. FLAG-JNK plasmids (pCDNA3 Flag Jnk1a1, pCDNA3 Flag Jnk1a1(apf), pCDNA3 Flag MKK7B2Jnk1a1, pCDNA3 Flag MKK7B2Jnk1a1(apf)) were purchased from Addgene. Kinases were immunoprecipitated from HEK293T cells as described above, except that two additional washes were done in kinase buffer (50 mM Tris-HCl,

pH 7.5, 25mM $MgCl_2$, 150mM NaCl, 10% Glycerol). FLAG-JNKs were either eluted using a 3X FLAG-peptide in kinase buffer for the fixed substrate concentration experiments or the resin was resuspended in an equal volume of kinase buffer for the fixed kinase concentration experiments. HA-ERK constructs were immunoprecipitated as above except that lysates were incubated for 2 hr with HA.11 Clone 16B12 Monoclonal Antibody ascites (Covance) and then 25uL of a 50% slurry of protein A sepharose CL-48 (GE Healthcare) for another hr at 4°C. After washing, the sepharose was resuspended in an equal volume of Kinase buffer. Immunoprecipitated kinases were incubated with bacterially expressed DGCR8 in Kinase buffer plus 1mM DTT, 20 μM ATP, and 10 μCi [γ-^{32}P]ATP for 30 min at 30°C, after which reactions were quenched by adding NuPAGe loading dye (Invitrogen) and electrophoresed through gradient Bis-Tris polyacrylamide gels (Invitrogen).

Analysis

^{32}P-containing gels or membranes were imaged using a Storm PhosphorImager (Molecular Dynamics). Data were plotted and fit in Igor (Wavemetrics).

Proteomics Sample Preparation

Bacmids were prepared from pFast-Bac1-HisDGCR8 in DH10BAC *E. coli*, according to the manufacturer's protocol (Invitrogen). Sf9 cells were grown at 27°C in Grace's media (Gibco) supplemented with 2 mM L-Glut, 1 X penicillin streptomycin solution (Sigma Aldrich), 0.25 μg/ml Amphotericin B solution (Sigma), and 10% fetal bovine serum (BD Biosciences) and transfected with bacmids using Cellfectin (Invitrogen). After 3 days, viral stocks were isolated and amplified. Hi-5 cells were grown at 27°C in shaker flasks in SfII900 medium (Gibco) supplemented as above. One hundred twenty-five mLs of Hi-5 cells at 2 X 10^6 cells/mL were infected with 2.5 mLs of P2 viral stock and grown at 27°C for 48 hr, harvested and frozen. Cells were lysed in 50 mL Buffer A (50 mM NaH_2PO_4 pH 8.0, 300 mM NaCl, and 20 mM imidazole) supplemented with 1% NP-40 and Complete EDTA-free protease inhibitor tablets (Roche) on ice for 30

min, and then spun at 30K relative centrifugal force for 30 min. Lysates were applied to a 5 mL HisTrap (GE) column, washed with 4 column volumes Buffer A and eluted with a 1 hr linear gradient of Buffer A to Buffer B (Buffer A plus 250 mM Imidazole). Fractions containing DGCR8 were pooled and concentrated using a 50 kDa cut-off Amicon filter (Millipore).

Affinity-purified DGCR8 samples were separated by SDS-PAGE. Several Coomassie-stained DGCR8 bands were cut and in-gel digested with trypsin (Shevchenko et al., 2006). For samples prepared from mammalian cells, peptides were subsequently captured and eluted step-wise from a strong cation exchange matrix (SCX). The SCX PolySULFOETHYLA (PolyLC) matrix was equilibrated with loading buffer (50mM sodium citrate, pH 2.5, and 2.5% acetonitrile). Bound peptides were eluted in each step with 2 washes of loading buffer adjusted with 25% ammonium hydroxide to six different pH values (3, 4, 5, 6, 7, and 10). The SCX peptide fractions were desalted by C18-based solid phase extraction (C18 zip-tip, Agilent) and dried by vacuum centrifugation.

Phosphopeptides were captured batch-wise with TiO_2 either directly from the in-gel trypsin digestion or following SCX pH fractionation. After 3 washes with loading buffer (90% acetonitrile, 5% TFA, 5% water), the captured phosphopeptides, were eluted twice with 25% ammonium hydroxide and immediately mixed with Tris base solution pH 8.5. Samples were desalted and dried as described above. Peptides from the insect samples that did not bind TiO_2 (supernatant) were further processed for any missed phosphopeptides by capturing them batch-wise on PhosphoSelect IMAC (Sigma-Aldrich) beads in loading buffer (30% acetic acid, 30% acetonitrile, 30% water). Captured peptides were eluted, desalted, and dried using the TiO_2 strategy described above. For peptide and phosphosite identification, each peptide fraction was further separated by reversed-phase (RP) liquid chromatography (LC) coupled online to a tandem mass spectrometer. This was performed at the Yale University Keck Proteomics Facility, using LTQ Orbitrap Elite coupled to a Waters nanoACQUITY Ultra High Performance Liquid

Chromatography (UPLC) system that uses a Waters Symmetry C18 180 µm x 20 mm trap column and a prepacked 1.7 µm, 75 µm x 250 mm nanoACQUITY UPLC column (35°C) for peptide separation.

SUPPLEMENTAL REFERENCES

Cox, J., Neuhauser, N., Michalski, A., Scheltema, R.A., Olsen, J.V., and Mann, M. (2011). Andromeda: a peptide search engine integrated into the MaxQuant environment. J Proteome Res *10*, 1794-1805.

Friedlander, M.R., Mackowiak, S.D., Li, N., Chen, W., and Rajewsky, N. (2012). miRDeep2 accurately identifies known and hundreds of novel microRNA genes in seven animal clades. Nucleic Acids Res *40*, 37-52.

Garai, A., Zeke, A., Gogl, G., Toro, I., Fordos, F., Blankenburg, H., Barkai, T., Varga, J., Alexa, A., Emig, D., *et al.* (2012). Specificity of linear motifs that bind to a common mitogen-activated protein kinase docking groove. Sci Signal *5*, ra74.

Gnad, F., Gunawardena, J., and Mann, M. (2011). PHOSIDA 2011: the posttranslational modification database. Nucleic Acids Res *39*, D253-260.

Gnad, F., Ren, S., Cox, J., Olsen, J.V., Macek, B., Oroshi, M., and Mann, M. (2007). PHOSIDA (phosphorylation site database): management, structural and evolutionary investigation, and prediction of phosphosites. Genome Biol *8*, R250.

Landthaler, M., Yalcin, A., and Tuschl, T. (2004). The human DiGeorge syndrome critical region gene 8 and its D. melanogaster homolog are required for miRNA biogenesis. Curr Biol *14*, 2162-2167.

Linding, R., Jensen, L.J., Ostheimer, G.J., van Vugt, M.A., Jorgensen, C., Miron, I.M., Diella, F., Colwill, K., Taylor, L., Elder, K., *et al.* (2007). Systematic discovery of in vivo phosphorylation networks. Cell *129*, 1415-1426.

Pawlicki, J.M., and Steitz, J.A. (2008). Primary microRNA transcript retention at sites of transcription leads to enhanced microRNA production. J Cell Biol *182*, 61-76.

Pimienta, G., Herbert, K.M., and Regan, L. (2011). A compound that inhibits the HOP-Hsp90 complex formation and has unique killing effects in breast cancer cell lines. Mol Pharm *8*, 2252-2261.

Shevchenko, A., Tomas, H., Havlis, J., Olsen, J.V., and Mann, M. (2006). In-gel digestion for mass spectrometric characterization of proteins and proteomes. Nat Protoc *1*, 2856-2860.
